# The divergent effects of astrocyte ceruloplasmin on learning and memory function in young and old mice

**DOI:** 10.1038/s41419-022-05459-4

**Published:** 2022-11-28

**Authors:** Zhong-Da Li, Haiyan Li, Shaomeng Kang, Yan-Ge Cui, Huiwen Zheng, Peina Wang, Kang Han, Peng Yu, Yan-Zhong Chang

**Affiliations:** 1 Ministry of Education Key Laboratory of Molecular and Cellular Biology, The Key Laboratory of Animal Physiology, Biochemistry and Molecular Biology of Hebei Province, College of Life Sciences, Hebei Normal University, No. 20 Nan’erhuan Eastern Road, Shijiazhuang, 050024 Hebei Province China; 2 College of Basic Medicine, Chengde Medical University, Chengde, Hebei Province China

**Keywords:** Cognitive ageing, Alzheimer's disease

## Abstract

Ceruloplasmin (CP) plays an important role in maintaining iron homeostasis. *Cp* gene knockout (*Cp*^*-/-*^) mice develop a neurodegenerative disease with aging and show iron accumulation in the brain. However, iron deficiency has also been observed in 3 M *Cp*^*-/-*^ mice. The use of systemic *Cp* gene knockout is insufficient to reveal specific functions for CP in the central nervous system. Considering recent discoveries that astrocytes synthetize the majority of brain CP, we generated astrocyte conditional *Cp* knockout (*Cp*^*Gfap*^*cKO*) mice, and found that iron contents decreased in the cerebral cortex and hippocampus of young (6 M) and old (18 M) *Cp*^*Gfap*^*cKO* mice. Further experiments revealed that 6 M *Cp*^*Gfap*^*cKO* mice exhibited impaired learning and memory function, while 18 M *Cp*^*Gfap*^*cKO* mice exhibited improved learning and memory function. Our study demonstrates that astrocytic *Cp* deletion blocks brain iron influx through the blood-brain-barrier, with concomitantly increased iron levels in brain microvascular endothelial cells, resulting in brain iron deficiency and down-regulation of ferritin levels in neurons, astrocytes, microglia and oligodendrocytes. At the young age, the synapse density, synapse-related protein levels, 5-hydroxytryptamine and norepinephrine, hippocampal neurogenesis and myelin formation were all decreased in *Cp*^*Gfap*^*cKO* mice. These changes affected learning and memory impairment in young *Cp*^*Gfap*^*cKO* mice. In old *Cp*^*Gfap*^*cKO* mice, iron accumulation with aging was attenuated, and was accompanied by the alleviation of the ROS-MAPK-apoptosis pathway, Tau phosphorylation and β-amyloid aggregation, thus delaying age-related memory decline. Overall, our results demonstrate that astrocytic *Cp* deletion has divergent effects on learning and memory function via different regulatory mechanisms induced by decreased iron contents in the brain of mice, which may present strategies for the prevention and treatment of dementia.

## Introduction

Ceruloplasmin (CP), first described by Holmberg and Laurell in 1948, is a ferroxidase [1]. CP is mainly synthesized by the liver and plays critical roles in the maintenance of iron homeostasis [2]. Previous studies have shown that the central nervous system (CNS) itself can express CP, which is predominantly produced in astrocytes [2–5]. Astrocyte CP, which is involved in the regulation of brain iron metabolism, can be soluble or attached to the cell membrane surface via a glycosylphosphatidylinositol (GPI) anchor [4–6].

Mutation or knockout of the *CP* gene results in aceruloplasminemia, which is characterized by iron overload in the brain, leading to neurodegenerative diseases (NDs) with aging in humans and mice [3, 7, 8]. Iron chelation or peripheral infusion of CP can attenuate the neurodegeneration and nigral iron elevation to different extents at different ages [9–11]. On the other hand, *Cp* deletion enhances nigral iron accumulation and exacerbates the related neurotoxicity in the substantia nigra of the MPTP-induced Parkinson’s disease (PD) mouse model [9, 11]. Likewise, *Cp* deficiency in Alzheimer’s disease (AD) mice facilitated iron overload, β-amyloid (Aβ) deposition in the hippocampus and memory impairment [12]. However, other studies have reported that *Cp* knockout mice at show reduced iron levels in cerebral cortex, striatum and hippocampus at a young age [13, 14]. Iron deficiency resulting from *Cp* knockout may decrease brain-derived neurotrophic factor expression in neurons, resulting in vulnerability to ischemic injury and an anxiety phenotype [13, 14]. Therefore, it is unclear whether CP facilitates iron uptake or iron release from the brain, and whether or not *Cp* knockout disrupts normal neural function. We believe this unresolved question is attributed to the limitations of a global *Cp* knockout mouse model.

To examine the specific role of CP in the brain, we generated astrocyte-specific *Cp* knockout (*Cp*^*Gfap*^*cKO*) mice by crossing *Cp*^*flox/flox*^ mice with *Gfap-cre* mice. To our surprise, in young (6 months old [6 M]) *Cp*^*Gfap*^*cKO* mice, learning and memory functions were impaired, whereas old (18 months old [18 M]) *Cp*^*Gfap*^*cKO* mice exhibited improved learning and memory, both of which are the result of iron depletion in the cerebral cortex or hippocampus, but affecting different regulatory pathways. Our results confirm that astrocyte-derived CP can facilitate iron uptake into brain parenchymal tissue from the circulation, and that specific knockout of *Cp* in astrocytes can inhibit iron uptake into the brain through the blood-brain-barrier (BBB). Decreased iron levels impaired learning and memory function via iron deficiency-induced synaptic formation, accompanied by a decline in myelination at a young age. However, decreased iron levels alleviated iron accumulation, reactive oxygen species (ROS) levels and age-related apoptosis in the *Cp*^*Gfap*^*cKO* mice.

## Results

### Conditional astrocytic *Cp* knockout impaired learning and memory functions but not anxiety-like behavior in 6-month-old mice

To identify the genotype of *Cp*^*Gfap*^*cKO* and *Cp*^*flox/flox*^ mice, we first confirmed the conditional knockout of *Cp* gene with PCR (Fig S1), we also found that both CP protein and *Cp* mRNA were decreased in the cerebral cortex and hippocampus of the *Cp*^*Gfap*^*cKO* mice with western blot and qPCR analysis (Fig S2A-D). We isolated astrocytes from neural tissue from adult mice using Magnetic Activated Cell Sorting (MACS) technology, and then we extracted RNA from the cells. We found that the copy number of *Cp* mRNA was also significantly down-regulated in *Cp*^*Gfap*^*cKO* mice compared to *Cp*^*flox/flox*^ mice (Fig S2E, F). To further verify that CP was absent in astrocytes in *Cp*^*Gfap*^*cKO* mice, we performed immunofluorescence staining and found that the astrocytes marker, glial fibrillary acidic protein (GFAP) and CP exhibited significantly less colocalization in the cerebral cortex (Fig S2G) and hippocampus (Fig S2H) of *Cp*^*Gfap*^*cKO* mice compared to *Cp*^*flox/flox*^ mice, suggesting the successful establishment of a model of conditional knockout of *Cp* in astrocytes.

**Fig. 1:**
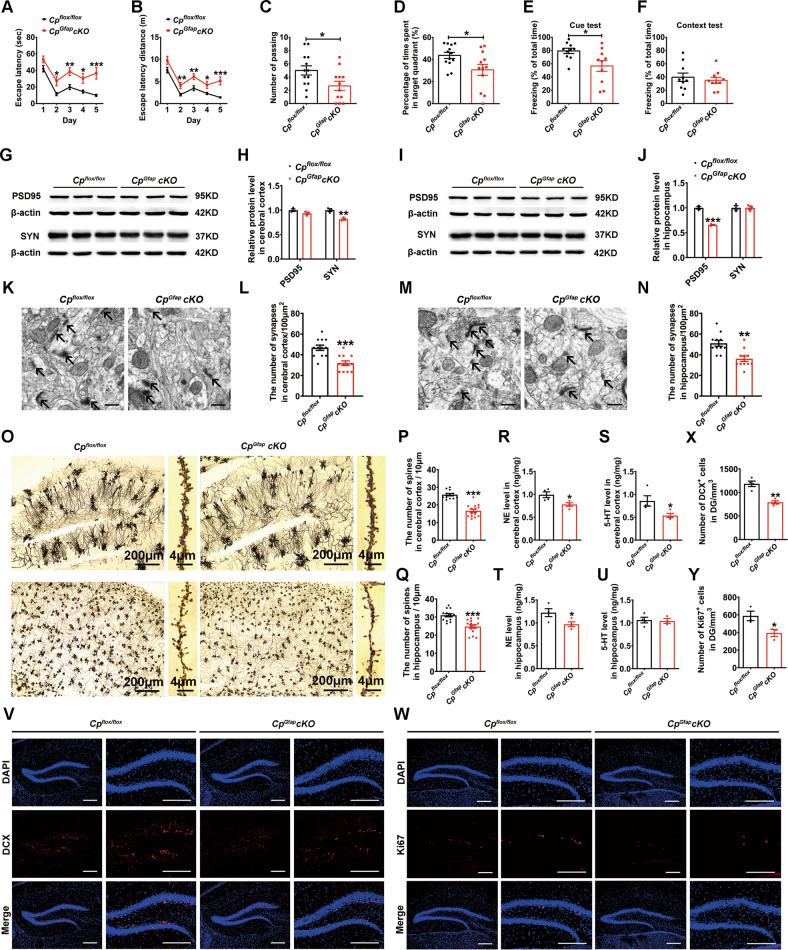
Conditional knockout of *Cp* in murine astrocytes impaired cognitive function and synaptic plasticity in the cerebral cortex and hippocampus. **A-D** MWM test of the *Cp*^*flox/flox*^ and *Cp*^*Gfap*^*cKO* mice. The latency time (**A**) and distance (**B**) to find the platform were recorded during the training trials from the 1^st^ day to the 5^th^ day. The platform was visible above the water during the 1^st^ and 2^nd^ day while hidden during the 3^rd^ to 5^th^ day. After training trails, the number of times the mice passed the hidden platform area (**C**) and the percentage of target quadrant passing time were recorded (**D**) on the 6^th^ day when the platform was removed and the mice were allowed to search for the area. (*n* = 13 for *Cp*^*flox/flox*^ group, *n* = 12 for *Cp*^*Gfap*^*cKO* group). **E-F** Conditional Fear Memory was tested using cued (**E**) and contextual (**F**) fear conditioning, and the percentage of freezing time was recorded after training (*n* = 10). **G**-**J** SYN and PSD95 protein levels in the cerebral cortex (**G**) and hippocampus (**I**), as detected by western blot analysis. β-actin was used as an internal reference. Densitometric analysis of the quantity of SYN and PSD-95 levels in the cerebral cortex (**H**) and hippocampus (**J**) are shown in the chart. (*n* = 3). **K**-**N** Synaptic density was assessed in the cerebral cortex (**K**) and hippocampus (**M**) by TEM; quantification of the number of synapses per 100 μm^2^ from the cerebral cortex (L) and hippocampus (**N**) are shown in the chart (*n* = 12 fields from 3 mice in cerebral cortex per group; *n* = 11 fields from 3 mice in hippocampus per group) Scale bar: 500 nm. **O-Q:** Golgi staining of the structural dendrites of the hippocampus and cerebral cortex (**O**) and the number of spines in the cerebral cortex (**P**) and hippocampus (**Q**) (*n* = 12 fields from 4 mice for *Cp*^*flox/flox*^ group and *n* = 14 fields from 4 mice for *Cp*^*Gfap*^*cKO* group in cerebral cortex; *n* = 13 fields from 4 mice for *Cp*^*flox/flox*^ group and *n* = 15 fields from 4 mice for *Cp*^*Gfap*^*cKO* group in hippocampus) were quantified after Golgi staining. **R-U:** The relative 5-HT and NE concentrations in the cerebral cortex (**R**-**S**) and hippocampus (**T**-**U**) were measured by HPLC in *Cp*^*flox/flox*^ and *Cp*^*Gfap*^*cKO* mice. The data are normalized to the levels in the control group (*n* = 4 per group in **R**-**T**; *n* = 4 for the *Cp*^*flox/flox*^ group and *n* = 3 for the *Cp*^*Gfap*^*cKO* group in the chart (**U**). **V**-**Y:** Immunofluorescence staining of DCX (**V**) and Ki67 (**W**) in the DG of the hippocampus in *Cp*^*flox/flox*^ and *Cp*^*Gfap*^*cKO* mice. Quantification of the number of the DCX- and Ki67-positive cells is presented in charts **X** (*n* = 4) and **Y** (*n* = 3); DAPI was used for nuclear staining. Scale bar: 200 μm. All the data are presented as the mean ± SEM; *p < 0.05; **p < 0.01; ***p < 0.001 vs. *Cp*^*flox/flox*^.

To assess spatial learning and memory function, we used the Morris water maze (MWM) with young (6 M) *Cp*^*Gfap*^*cKO* mice and control (*Cp*^*flox/flox*^) mice. The *Cp*^*Gfap*^*cKO* mice spent significantly more time and covered a greater distance to find the hidden platform, during the five consecutive days of training (Fig. 1A, B). After the 5-day training trials, the platform in the water was removed, and the number of passes across the position of the original platform was evaluated. In the *Cp*^*Gfap*^*cKO* mice, the number of passes and the percentage of target quadrant passing time were significantly decreased compared to the control mice (Fig. 1C, D). In the test of fear memory conditioning, the *Cp*^*Gfap*^*cKO* mice exhibited decreased freezing times during the cue test (Fig. 1E), in contrast to the context test in which no significant difference was observed (Fig. 1F). To examine the anxiety-like behavior in the animals, we applied open field and elevated plus maze (EPM) tests [15, 16]. However, there were no significant differences in the percentage of time and distance of center exploration in the open field test (Fig S3A, B) or in the open arm of the EPM test (Fig S3C, D). These results indicate that *Cp* deficiency in astrocytes may impair learning and memory but not stimulate anxiety-like behavior in mice.

### Astrocytic *Cp* conditional knockout altered synaptic formation and adult hippocampal neurogenesis

To study the mechanism of the above effect on learning and memory, we turned our attention to synaptic density and plasticity. Synaptophysin (SYN) is a presynaptic protein widely localized to synaptic vesicles [17]. Postsynaptic density protein 95 (PSD95) is another major synaptic protein, whose levels are reduced in neurodegenerative disorders [18]. Western blot analysis showed that the SYN levels were observably decreased in the cerebral cortex, but the levels of PSD95 were unaffected (Fig. 1G, H). However, in the hippocampus, the level of PSD95 was significantly down-regulated, while the SYN remained unchanged (Fig. 1I, J).

To further understand the changes to synapses, we assessed synaptic morphology by transmission electron microscopy (TEM). The number of synapses in both the cerebral cortex and hippocampus of *Cp*^*Gfap*^*cKO* mice was lower than that of the control groups (Fig. 1K-N). Golgi staining showed that the spine densities were also significantly decreased in the hippocampus and cerebral cortex of *Cp*^*Gfap*^*cKO* mice (Fig. 1O-Q).

5-Hydroxytryptamine (5-HT) and norepinephrine (NE), members of the monoamine neurotransmitter family, are associated with learning, memory function and anxiety-like behavior [19, 20]. We used high-performance liquid chromatography (HPLC) to measure the levels of NE and 5-HT in the cerebral cortex and hippocampus. In *Cp*^*Gfap*^*cKO* mice, both the NE and 5-HT levels were diminished in the cerebral cortex (Fig. 1R, S), but in the hippocampus only the levels of NE decreased, while the 5-HT levels were comparable to those in the *Cp*^*flox/flox*^ group (Fig. 1T, U). These results suggest that astrocytic *Cp*-deficiency limits the production of the monoamine neurotransmitter family.

Adult hippocampal neurogenesis may play an important role in learning and memory regulation [21, 22]. We therefore examined the density of DCX^+^ immature neurons in the dentate gyrus (DG) of the hippocampus. We found a decreased number of DCX^+^ (immature neuron marker) and Ki67^+^ (neural proliferating marker) cells in the DG of *Cp*^*Gfap*^*cKO* mice (Fig. 1V-Y), suggesting some level of neurogenesis deficiency under *Cp* depletion.

### Astrocytic *Cp* conditional knockout impaired myelin sheath development

**Fig. 2:**
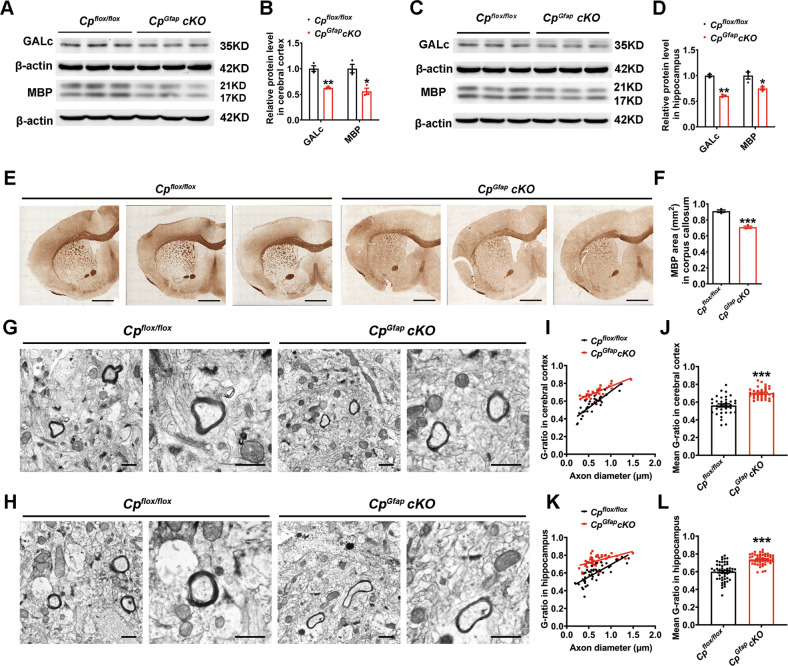
Impairment of myelin formation in the brain of *Cp*^*Gfap*^*cKO* mice. **A-D** Western blot analysis of GALc and MBP expression levels in the cerebral cortex (**A**) and hippocampus (**C**) of *Cp*^*flox/flox*^ and *Cp*^*Gfap*^*cKO* mice. Quantification of the expression of GALc and MBP in the cerebral cortex (**B**) and hippocampus (**D**) was performed. (*n* = 3). **E** Immunohistochemistry images of MBP immunostaining in coronal brain sections from *Cp*^*flox/flox*^ and *Cp*^*Gfap*^*cKO* mice. Scale bar: 1 mm. **F:** Quantification of MBP area (mm^2^) in corpus callosum (*n* = 3). **G-L** The myelin was examined in the cerebral cortex (**G**) and hippocampus (**H**) by TEM, **I** and **J** show the corresponding G-ratio in the cerebral cortex (*n* = 34 from 3 mice); **K** and **L** show the G-ratio in the hippocampus (*n* = 50 from 3 mice for the *Cp*^*flox/flox*^ group and *n* = 48 from 3 mice for the *Cp*^*Gfap*^*cKO* group). Scale bar: 1 μm. All the data are presented as the mean ± SEM; *p < 0.05; **p < 0.01; ***p < 0.001 vs. *Cp*^*flox/flox*^.

Previous studies have demonstrated that myelin impairment is associated with aggravated anxiogenic-related behavior and declined memory function [23–26]. We therefore examined the levels of the myelin-related protein, MBP (myelin basic protein), and GALc (galactosylceramidase), in both the cerebral cortex and hippocampus. The knockout mice exhibited a down-regulation of MBP and GALc expression in the cerebral cortex (Fig. 2A, B) and hippocampus (Fig. 2C, D). Immunohistochemistry staining also demonstrated a decrease of MBP expression in the corpus callosum of *Cp*^*Gfap*^*cKO* mice (Fig. 2E, F). In addition, the TEM data reveal an increased myelin G-ratio (ratio between the inner and outer diameter of the myelin sheath) in the *Cp*^*Gfap*^*cKO* mice, with a reduced number of large-diameter axons in the cerebral cortex (Fig. 2G, I, J) and hippocampus (Fig. 2H, K, L). These results suggest that the behavioral abnormalities conferred by astrocytic CP deficiency may be due to decreases in synaptic function and myelin sheath integrity.

### Astrocytic *Cp* conditional knockout decreased iron contents in the cerebral cortex and hippocampus

**Fig. 3:**
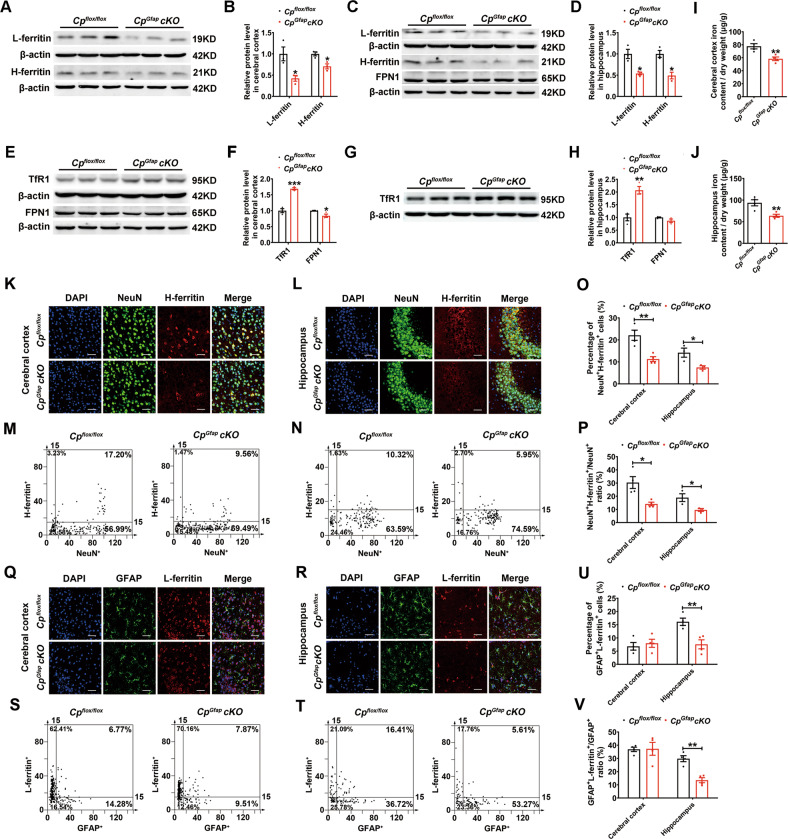
Astrocytic *Cp* knockout decreased ferritin expression and iron contents in the cerebral cortex and hippocampus. **A**-**D** Western blot detection of L-ferritin and H-ferritin levels in the cerebral cortex (**A**) and hippocampus (**C**). Quantification of the immunostained bands (L-ferritin and H-ferritin) are presented in **B** and **D**, respectively. (*n* = 3). **E**-**H:** Western blot analysis of FPN1 and TfR1 levels in the cerebral cortex (**E**) and hippocampus (**C**, **G)**. **F** and **H** are the quantification of the light density of TfR1- and FPN1-immunostained bands. (*n* = 3). **I**, **J:** Total iron content was measured by ICP-MS in the cerebral cortex (**I**) and hippocampus (**J**). (*n* = 4). **K**-**P:** Double immunofluorescence staining of H-ferritin and NeuN in the cerebral cortex (**K**) and hippocampus (**L**) of *Cp*^*flox/flox*^ and *Cp*^*Gfap*^*cKO* mice. The intensity of NeuN^+^ and H-ferritin^+^ cells in the cerebral cortex (**M**) and hippocampus (**N**) is shown in scatter diagrams. Quantification of the percentage of the NeuN^+^H-ferritin^+^ cells (**O**) and the ratio of NeuN^+^H-ferritin^+^ / NeuN^+^ (**P**) are presented on the histogram plots. (*n* = 4 in the cerebral cortex and *n* = 3 in the hippocampus). **Q**-**V:** Double immunofluorescence labeling of L-ferritin and GFAP in the cerebral cortex (**Q**) and hippocampus (**R**) of *Cp*^*flox/flox*^ and *Cp*^*Gfap*^*cKO* mice. The intensity of GFAP^+^ and L-ferritin^+^ cells in the cerebral cortex (**S**) and hippocampus (**T**) is shown in scatter diagrams. Quantification of the percentage of the GFAP^+^L-ferritin^+^ cells (**U**) and the ratio of GFAP^+^L-ferritin^+^ / GFAP^+^ (**V**) are presented on the histogram plots. (*n* = 4). All data are shown as the mean ± SEM; *p < 0.05, **p < 0.01, ***p < 0.001 vs. *Cp*^*flox/flox*^.

The H-ferritin and L-ferritin levels were decreased in both the cerebral cortex and the hippocampus of the *Cp*^*Gfap*^*cKO* mice (Fig. 3A-D). Additionally, there was marked up-regulation of TfR1 (Transferrin receptor 1) protein levels in the cerebral cortex and the hippocampus, while FPN1 (Ferroportin 1) protein was significantly down-regulated in the cerebral cortex, but not in the hippocampus, in *Cp*^*Gfap*^*cKO* mice (Fig. 3C, E-H).

We used synchrotron radiation micro-X-ray fluorescence (μ-XRF) technology [27], and observed that the iron contents were decreased in both the cerebral cortex and hippocampus in the *Cp*^*Gfap*^*cKO* group (Fig S4A). We also used Perl’s iron staining (Fig S4B) and inductively coupled plasma-mass spectrometry (ICP-MS; Fig. 3I, J), and found that the iron contents were markedly decreased in the cerebral cortex and hippocampus regions in the *Cp*^*Gfap*^*cKO* group. These data are consistent with the observed changes in ferritin levels. Moreover, the distribution and contents of copper were also detected with μ-XRF and ICP-MS, and data showed that copper levels were not changed in cerebral cortex and hippocampus in *Cp*^*Gfap*^*cKO* mice compared with those of *Cp*^*flox/flox*^ mice (Fig S4C-E).

To clarify the variations in iron distribution between neurons and glia, we assessed the levels of ferritin in the cells of the cortical and hippocampal regions. Compared to the control group, the *Cp*^*Gfap*^*cKO* exhibited a significantly lower percentage of NeuN^+^ H-ferritin^+^ cells and a lower ratio of NeuN^+^ H-ferritin^+^ / NeuN^+^ both in the cerebral cortex and hippocampus (Fig. 3K-P). To further understand the changes in brain iron homeostasis in *Cp*^*Gfap*^*cKO* mice, we evaluated the L-ferritin levels in three types of glia: astrocytes, microglia and oligodendrocytes. First, we immunostained GFAP (astrocytes marker) and L-ferritin in the cerebral cortex and hippocampus, and observed that the percentage of GFAP^+^L-ferritin^+^ cells and the ratio of GFAP^+^L-ferritin^+^ / GFAP^+^ were decreased in the hippocampus of *Cp*^*Gfap*^*cKO* mice compared to *Cp*^*flox/flox*^ mice (Fig. 3R, T-V), while the proportions of these cells did not change in the cerebral cortex (Fig. 3Q, S, U, V). Next, we immunostained Iba-1 (microglia marker) and L-ferritin, and found that in both cortical and hippocampal areas, the percentage of Iba-1^+^L-ferritin^+^ cells and the ratio of Iba-1^+^L-ferritin^+^ / Iba-1^+^ were down-regulated in *Cp*^*Gfap*^*cKO* mice compared to the *Cp*^*flox/flox*^ mice (Fig S5A-F). Finally, we immunostained CC1 (oligodendrocyte marker) and L-ferritin in the cerebral cortex and hippocampus, and the percentage of the CC1^+^L-ferritin^+^ cells and the ratio of CC1^+^L-ferritin^+^ / CC1^+^ were decreased in *Cp*^*Gfap*^*cKO* mice (Fig S5G-L). Taken together, these results indicate that astrocyte-conditional knockout of *Cp* mice exhibit an overall iron-deficient phenotype in the cerebral cortex and hippocampus.

### Astrocytic CP deficiency promoted iron accumulation in brain microvascular endothelial cells (BMVEC)

**Fig. 4:**
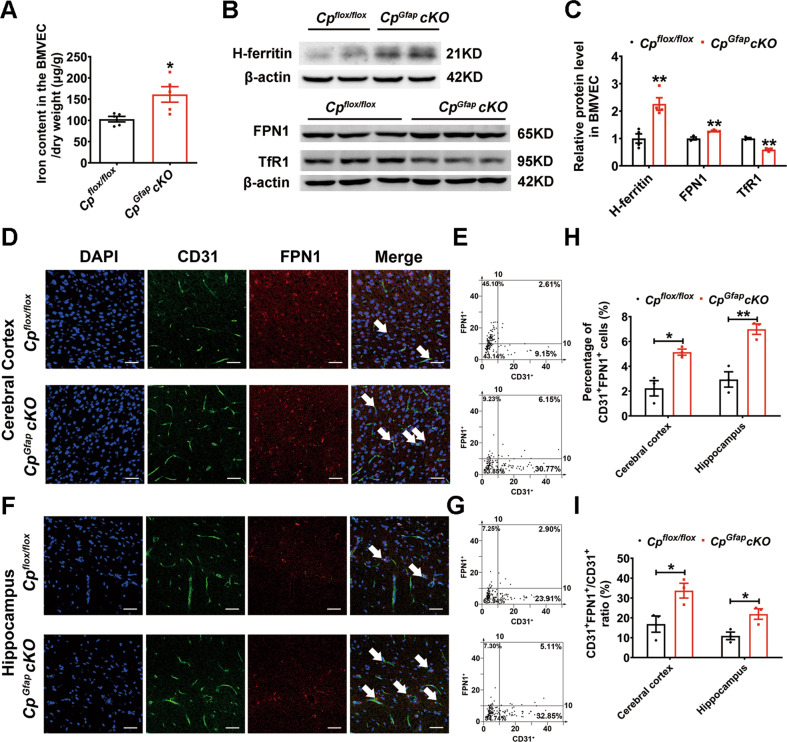
Iron accumulation in BMVECs in *Cp*^*Gfap*^*cKO* mice. **A** Total iron content was detected by ICP-MS in BMVECs from *Cp*^*flox/flox*^ and *Cp*^*Gfap*^*cKO* mice. (*n* = 5). **B:** Western blot analysis of H-ferritin, FPN1 and TfR1 expression in BMVECs from *Cp*^*flox/flox*^ and *Cp*^*Gfap*^*cKO* mice. **C**. Quantification of H-ferritin, FPN1 and TfR1 protein levels in BMVECs was normalized to β-actin, which was relative to that of *Cp*^*flox/flox*^ mice. (*n* = 4 for H-ferritin; *n* = 3 for FPN1 and TfR1). **D-I:** Double immunofluorescence labeling of FPN1 and CD31 in the cerebral cortex (**D**) and hippocampus (**F**) in *Cp*^*flox/flox*^ and *Cp*^*Gfap*^*cKO* mice. The intensity of CD31^+^ and FPN1^+^ cells in the cerebral cortex (**E**) and hippocampus (**G**) is shown in scatter diagrams. Quantification of the percentage of the CD31^+^FPN1^+^ cells (**H**) and the ratio of CD31^+^FPN1^+^ / CD31^+^ (**I**) are shown. (*n* = 3). DAPI was used for nuclear staining. Scale bar: 50 μm. All data are shown as the mean ± SEM; *p < 0.05, **p < 0.01 vs. *Cp*^*flox/flox*^.

Brain microvascular endothelial cells (BMVECs) participate in iron uptake from the peripheral blood into brain tissue [4, 6]. We measured the levels of iron and iron-related proteins in BMVECs. The ICP-MS results revealed an accumulation of iron in BMVECs in the *Cp*^*Gfap*^*cKO* group (Fig. 4A). H-ferritin and FPN1 protein expression levels in BMVECs were remarkably increased in the *Cp*^*Gfap*^*cKO* group, while the TfR1 levels were significantly decreased (Fig. 4B, C). We also used immunofluorescence to label CD31 (BMVEC marker) and FPN1, and discovered that the percentage of CD31^+^FPN1^+^ cells and the ratio of CD31^+^FPN1^+^ / CD31^+^ were increased in both the cerebral cortex and hippocampus in *Cp*^*Gfap*^*cKO* mice (Fig. 4D-I), which is consistent with the western blot analysis data. These results suggest that the disruption of CP expression in astrocytes leads to an accumulation of iron in BMVECs by means of blocking iron flux from BMVECs into the CNS via FPN1.

### Astrocytic CP deficiency promoted Hephaestin up-regulation in neurons

**Fig. 5:**
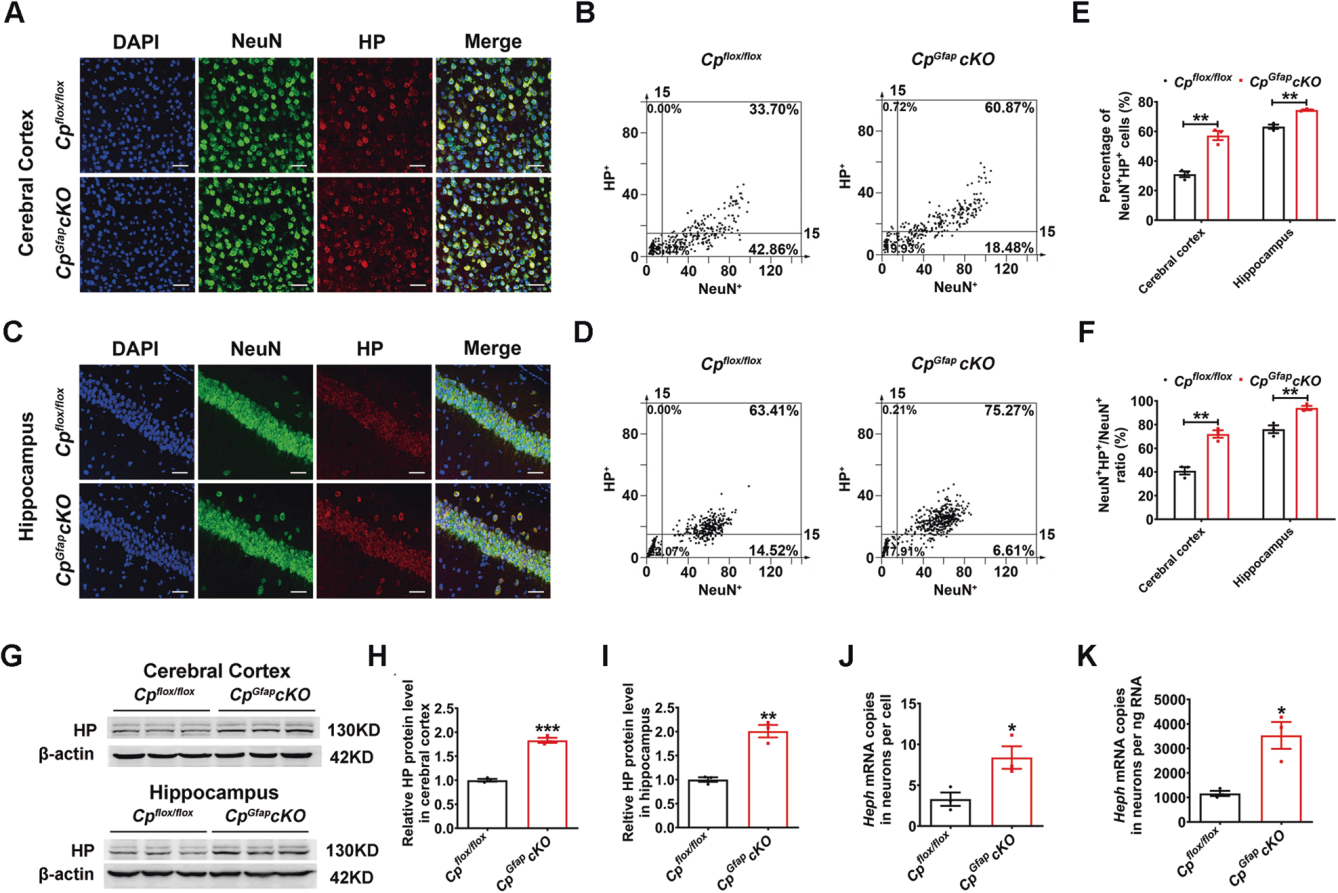
The expression of hephaestin increased in neurons of *Cp*^*Gfap*^*cKO* mice. **A**-**F** Double immunofluorescence labeling of HP and NeuN in the cerebral cortex (**A**) and hippocampus (**C**) in *Cp*^*flox/flox*^ and *Cp*^*Gfap*^*cKO* mice. The intensity of NeuN^+^ and HP^+^ cells in the cerebral cortex (**B**) and hippocampus (**D**) is shown in scatter diagrams. Quantification of the percentage of the NeuN^+^HP^+^ cells (**E**) and the ratio of NeuN^+^HP^+^ / NeuN^+^ (**F**) are shown in the bar graphs. (*n* = 3). DAPI was used for nuclear staining. Scale bar: 50 μm. **G-I:** Western blot detection of HP levels in the cerebral cortex and hippocampus (**G**). Quantification of the amount of HP in the cerebral cortex (**H**) and hippocampus (**I**). (*n* = 3). Neurons were isolated using a Neuron Isolation Kit and *Heph* mRNA levels were detected by qPCR, **J** and **K** show the quantified amounts of *Heph* mRNA per cell and per ng RNA, respectively. (*n* = 3). All data are shown as the mean ± SEM; *p < 0.05; **p < 0.01; ***p < 0.001 vs. *Cp*^*flox/flox*^.

Hephaestin (HP) is another multi-copper ferroxidase involved in iron export from cells, and a homologue of CP [6]. By the double immunostaining of NeuN/HP, we observed that the percentage of the NeuN^+^HP^+^ cells and the ratio of NeuN^+^HP^+^ / NeuN^+^ were significantly increased in both the cerebral cortex and hippocampus in the CP-deficient astrocyte group (Fig. 5A-F). To assess the total expression of HP in the cerebral cortex and hippocampus, we used western blot analysis and found that the expression of HP was increased in these regions (Fig. 5G-I). To confirm the up-regulation of neuronal HP, we also isolated neurons by MACS technology, and extracted the total RNA from the cells. We found that the expression of *Heph* mRNA was significantly increased in the neurons from *Cp*^*Gfap*^*cKO* mice compared to *Cp*^*flox/flox*^ mice (Fig. 5J, K). Meanwhile, we also detected CP in neurons, but found that neuronal CP expression was not altered between the *Cp*^*Gfap*^*cKO* and *Cp*^*flox/flox*^ mice (Fig S6). These results indicate that *Cp* deletion in astrocytes increases HP expression in neurons, which may promote iron efflux from neurons and lead to further iron deficiency in the cells.

### Astrocytic CP deficiency in aged mice improved learning and memory function via the recovery of cortical and hippocampal iron to young levels

**Fig. 6:**
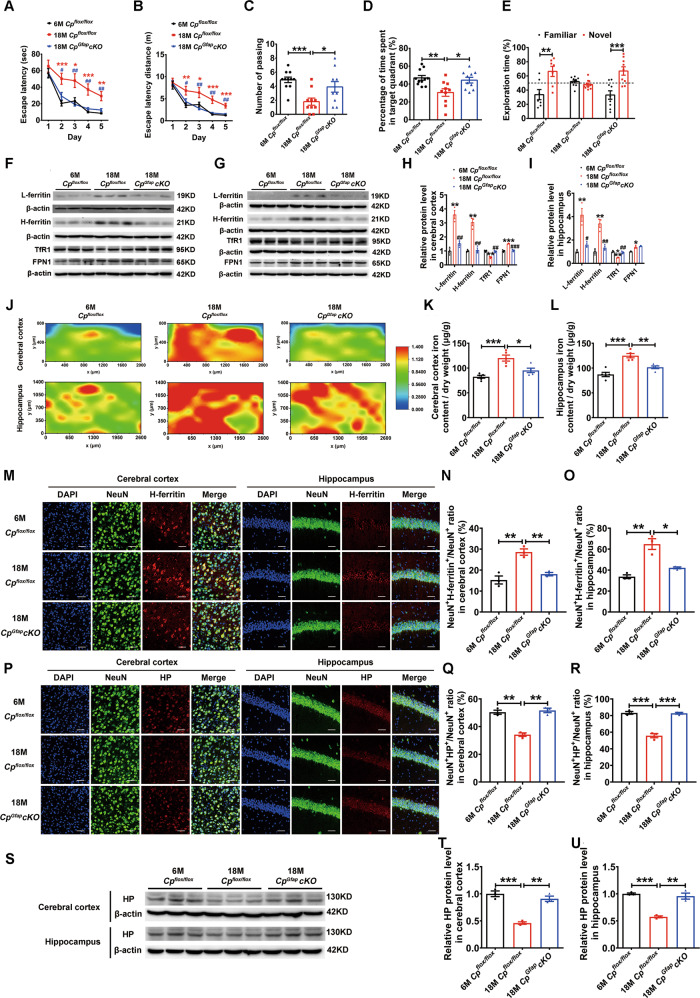
Astrocytic *Cp* knockout in aged mice improved spatial learning and memory function with decreased iron content in the cerebral cortex and hippocampus. **A-D** MWM test of 6-month-old (6 M, young) *Cp*^*flox/flox*^, 18-month-old (18 M, old) mice and 18 M *Cp*^*Gfap*^*cKO* mice. Latency time (**A**) and distance (**B**) to the platform were recorded during the training trials from the 1^st^ day to the 5^th^ day. After the training trails, the number of times the mice passed the hidden platform area (**C**) and the percentage of time spent in the target quadrant were recorded (**D**). (*n* = 11 for the 6 M *Cp*^*flox/flox*^ group, *n* = 10 for the 18 M *Cp*^*flox/flox*^ and 18 M *Cp*^*Gfap*^*cKO* groups, mean ± SEM, *p < 0.05, **p < 0.01, ***p < 0.001, 6 M *Cp*^*flox/flox*^ vs. 18 M *Cp*^*flox/flox*^; ^*#*^*p* < 0.05, ^##^p < 0.01, 18 M *Cp*^*Gfap*^*cKO* vs. 18 M *Cp*^*flox/flox*^). **E:** The time spent in the familiar and novel positions and object exploration during the NOR test (*n* = 7 for the 6 M *Cp*^*flox/flox*^ group, *n* = 10 for the 18 M *Cp*^*flox/flox*^ group and *n* = 9 for the 18 M *Cp*^*Gfap*^*cKO* group, **p < 0.01, ***p < 0.001, mean ± SEM). **F**-**I:** Western blot analysis of L-ferritin, H-ferritin, TfR1 and FPN1 levels in the cerebral cortex (**F**) and hippocampus (**G**). Quantification of the density of immunostained bands (L-ferritin, H-ferritin, TfR1 and FPN1) is presented in **H** and **I**, respectively. (*n* = 3, *p < 0.05, **p < 0.01, ***p < 0.001, 6 M *Cp*^*flox/flox*^ vs. 18 M *Cp*^*flox/flox*^; ^#^*p* < 0.05, ^##^*p* < 0.01, ^###^*p* < 0.001, 18 M *Cp*^*Gfap*^*cKO* vs. 18 M *Cp*^*flox/flox*^). **J:** The distribution and iron status in the cerebral cortex and hippocampus of 6 M *Cp*^*flox/flox*^, 18 M *Cp*^*flox/flox*^ and 18 M *Cp*^*Gfap*^*cKO* mice were detected by μ-XRF. The six images show a heatmap of the iron distribution. **K**-**L** Total iron content in the cerebral cortex (**K**) and hippocampus (**L**) were as measured by ICP-MS in the three groups (*n* = 5 per group; mean ± SEM; *p < 0.05, **p < 0.01, ***p < 0.001). **M**-**O:** Double immunofluorescence staining of H-ferritin and NeuN in the cerebral cortex and hippocampus of the 6 M *Cp*^*flox/flox*^, 18 M *Cp*^*flox/flox*^ and 18 M *Cp*^*Gfap*^*cKO* mice. Quantification of the ratio of NeuN^+^H-ferritin^+^ / NeuN^+^ in the cerebral cortex (**N**) and hippocampus (**O**) is presented in histogram plots. (mean ± SEM, *n* = 3). DAPI was used for nuclear staining. Scale bar: 50 μm. **P**-**R** Double immunofluorescence staining of HP and NeuN in the cerebral cortex and hippocampus. The ratio of NeuN^+^HP^+^ / NeuN^+^ in cerebral cortex (**Q**) and hippocampus (**R**) is quantified in histogram plots. (mean ± SEM, *n* = 3). DAPI was used for nuclear staining. Scale bar: 50 μm. **S**-**U** Western blot analysis of HP levels in the cerebral cortex and hippocampus. Quantification of the density of immunostained bands is presented in **T** and **U**. (mean ± SEM, *n* = 3, **p < 0.01, ***p < 0.001, 6 M *Cp*^*flox/flox*^).

We used the MWM to test the spatial learning and memory function of 18 M *Cp*^*flox/flox*^ and *Cp*^*Gfap*^*cKO* mice. Interestingly, it took less time for the old 18 M *Cp*^*Gfap*^*cKO* mice to arrive at the hidden platform in the 2^nd^, 3^rd^, 4^th^ and 5^th^ day of training compared to the 18 M *Cp*^*flox/flox*^ mice (Fig. 6A). Additionally, the escape latency distance to the platform was markedly decreased in the 18 M *Cp*^*Gfap*^*cKO* mice over the same four days (Fig. 6B), while the probe test revealed an increased number of passes across the platform location (Fig. 6C) and also an increase in the percent of target quadrant passing time in the aged *Cp*^*Gfap*^*cKO* mice compared to the same aged control mice (Fig. 6D). These results are all contrary to those acquired in young *Cp*^*flox/flox*^ and *Cp*^*Gfap*^*cKO* mice. To explore how these changes in old mice compare to young controls, we performed the experiment again using young 6 M *Cp*^*flox/flox*^ mice, old 18 M *Cp*^*flox/flox*^ mice and old 18 M *Cp*^*Gfap*^*cKO* mice. We found that the spatial learning and memory function declined in the 18 M *Cp*^*flox/flox*^ mice, compared with the 6 M *Cp*^*flox/flox*^ mice; however, the 18 M *Cp*^*Gfap*^*cKO* mice exhibited an improved learning ability that was similar to that of the young *Cp*^*flox/flox*^ mice (Fig. 6A-D). We also evaluated the recognition ability between the three groups of mice using the new object recognition test (NOR), and found that the control 6 M *Cp*^*flox/flox*^ mice spent more time in the area close to the novel object (Fig. 6E) than the familiar object, aged *Cp*^*flox/flox*^ mice spent a similar time in the familiar and novel object areas, while 18 M *Cp*^*Gfap*^*cKO* mice spent more time in the novel object area (Fig. 6E) than the familiar object area, thus following the same pattern as the 6 M *Cp*^*flox/flox*^ mice as in the other learning and memory tests. The results so far appear to indicate that astrocytic CP deficiency can improve learning and memory function in old mice.

To determine whether the improvement of cognitive function was related to the iron levels in the old *Cp*^*Gfap*^*cKO* mice, we evaluated the iron in the cerebral cortex and hippocampus and found that both L-ferritin and H-ferritin expression were increased significantly in the 18 M *Cp*^*flox/flox*^ mice compared with that in the 6 M *Cp*^*flox/flox*^ mice, while the expression of these proteins was decreased in the 18 M *Cp*^*Gfap*^*cKO* mice compared to the 18 M *Cp*^*flox/flox*^ mice. The level of TfR1 protein was markedly decreased with aging, whereas increased in 18 M *Cp*^*Gfap*^*cKO* compared to 18 M *Cp*^*flox/flox*^ mice. FPN1 was increased with aging both in the cerebral cortex and hippocampus, but decreased in the cerebral cortex and unchanged in the hippocampus of 18 M *Cp*^*Gfap*^*cKO* mice compared to the same age control mice (Fig. 6F-I). Again, we used μ-XRF analysis to investigate the iron levels in the cerebral cortex and hippocampus in 6 M *Cp*^*flox/flox*^ mice, 18 M *Cp*^*flox/flox*^ mice and 18 M *Cp*^*Gfap*^*cKO* mice. The cerebral cortex and hippocampus exhibited an increased iron signal with aging in *Cp*^*flox/flox*^ mice, while in the 18 M *Cp*^*Gfap*^*cKO* group, the iron signals were diminished to levels compared with the 18 M *Cp*^*flox/flox*^ mice (Fig. 6J). Moreover, we used ICP-MS to assess the total iron contents in the cerebral cortex and hippocampus. Consistent with the results of the ferritin analysis, the 18 M *Cp*^*flox/flox*^ mice exhibited high level of iron vs. 6 M *Cp*^*flox/flox*^ mice, while the 18 M *Cp*^*Gfap*^*cKO* mice had decreased iron levels compared with the 18 M CP^*flox/flox*^ mice (Fig. 6K, L). To rigorously explore the iron levels in the brain, we evaluated the levels of H-ferritin in the neurons of the cerebral cortex and hippocampus. The ratio of NeuN^+^ H-ferritin^+^ / NeuN^+^ in both the cerebral cortex and hippocampus were up-regulated with aging, but down-regulated in 18 M *Cp*^*Gfap*^*cKO* compared with the 18 M *Cp*^*flox/flox*^ mice (Fig. 6M-O). We were then curious about whether HP protein was increased in 18 M *Cp*^*Gfap*^*cKO* mice as it was in the 6 M *Cp*^*Gfap*^*cKO* mice, so we employed double immunofluorescence staining of NeuN/HP. The ratio of NeuN^+^HP^+^ / NeuN^+^ were markedly decreased with aging in *Cp*^*flox/flox*^ mice, but increased in both the cerebral cortex and hippocampus of the 18 M *Cp*^*Gfap*^*cKO* mice compared with the 18 M *Cp*^*flox/flox*^ mice (Fig. 6P-R). The expression of HP both in the cerebral cortex and hippocampus was detected by western blot analysis, which showed that HP was decreased in 18 M *Cp*^*flox/flox*^ mice vs. 6 M *Cp*^*flox/flox*^ mice but increased in 18 M *Cp*^*Gfap*^*cKO* mice vs. 18 M *Cp*^*flox/flox*^ mice in both regions (Fig. 6S-U). These results suggest that astrocytic CP deficiency can also decrease iron levels in the cerebral cortex and hippocampus, with HP expression increased at old age, attenuating age-dependent brain iron accumulation.

### CP deficiency in astrocytes of old mice prevents age-related apoptosis and ROS accumulation in the cerebral cortex and hippocampus

**Fig. 7:**
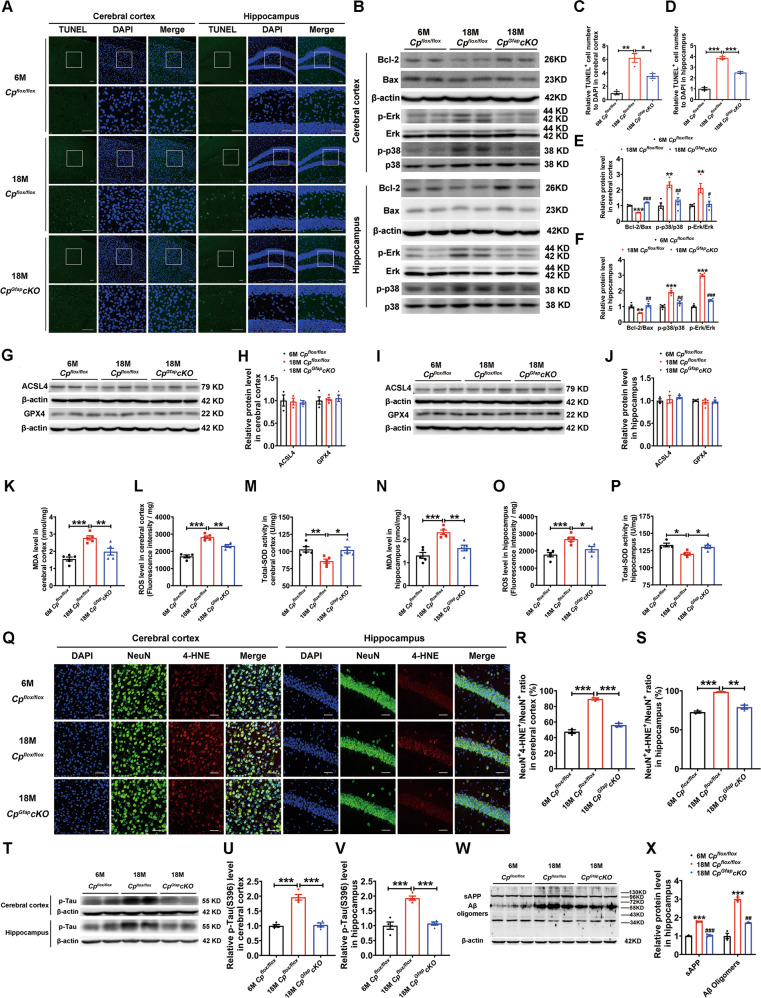
Astrocytic *Cp* deficiency in aged mice alleviated age-dependent apoptosis in both the cerebral cortex and hippocampus with suppression of ROS. **A**, **C**, **D** TUNEL staining in the cerebral cortex and hippocampus. The number of apoptotic cells was quantified in the cerebral cortex (**C**) and hippocampus (**D**). (*n* = 3, mean ± SEM, *p < 0.05, **p < 0.01, ***p < 0.001). Scale bar: 50 μm, DAPI was used for nuclear staining. **B**, **E**, **F:** Western blot analysis of the Bcl-2, Bax, phosphorylated Erk and phosphorylated p38 levels. The ratio of Bcl-2/Bax, p-Erk/Erk and p-p38/p38 was calculated in the cerebral cortex (**E**) and hippocampus (**F**) (*n* = 3, **p < 0.01, ***p < 0.001, 6 M *Cp*^*flox/flox*^ vs. 18 M *Cp*^*flox/flox*^; ^#^*p* < 0.05, ^##^*p* < 0.01, ^###^*p* < 0.001, 18 M *Cp*^*Gfap*^*cKO* vs. 18 M *Cp*^*flox/flox*^). **G**-**J:** ACSL4 and GPX4 (ferroptosis-related protein) were assessed by western blot analysis in the cerebral cortex (**G**) and hippocampus (**I**). The expression levels of ACSL4 and GPX4 were quantified (**H**, **J**). (*n* = 3 for ACSL4 and *n* = 4 for GPX4). **K**-**P:** The levels of MDA, ROS and total SOD were assessed in the cerebral cortex (**K**, **L**, **M**) and hippocampus (**N**, **O**, **P**). (*n* = 4 to 5; mean ± SEM; *p < 0.05, **p < 0.01, ***p < 0,001). **Q**-**S:** Double immunofluorescence staining of 4-HNE and NeuN in the cerebral cortex and hippocampus. The ratio of NeuN^+^4-HNE^+^ / NeuN^+^ in the cerebral cortex (**R**) and hippocampus (**S**) is quantified in histogram plots. (mean ± SEM, *n* = 3). DAPI was used for nuclear staining. Scale bar: 50 μm. **T**-**V:** The S396 phosphorylation status of Tau in the cortex and hippocampus of the indicated groups was detected by western blot analysis. The data are quantified in panels **U** and **V**. (*n* = 4; ***p < 0.001). **W**-**X:** Soluble APP (sAPP) and Aβ oligomer levels were evaluated by western blot analysis in the hippocampus (**W**). Quantification of the sAPP and Aβ oligomer (**X**) levels in the hippocampus from the image in panel **W**. (*n* = 3 per group; ***p < 0.001, 6 M *Cp*^*flox/flox*^ vs. 18 M *Cp*^*flox/flox*^; ^#*#*^*p* < 0.01, ^###^*p* < 0.001, 18 M *Cp*^*Gfap*^*cKO* vs. 18 M *Cp*^*flox/flox*^).

To investigate the mechanism of the recovery in learning and memory deficits in aging *Cp*^*Gfap*^*cKO* mice, we examined apoptosis in the cerebral cortex and hippocampus using terminal deoxynucleotidyltransferase-mediated dUTP nick-end labeling (TUNEL) staining. As expected, the amounts of apoptotic cells in these brain regions increased markedly with aging, while the number of apoptotic cells was significantly lower in the 18 M *Cp*^*Gfap*^*cKO* mice compared with the 18 M *Cp*^*flox/flox*^ mice (Fig. 7A, C, D). Bcl-2 (anti-apoptosis) and Bax (pro-apoptosis) proteins play important roles in regulating apoptosis. From western blot analysis, we found that the ratio of Bcl-2 to Bax was decreased in both the cerebral cortex and hippocampus with aging, but increased in the 18 M *Cp*^*Gfap*^*cKO* mice compared with the 18 M *Cp*^*flox/flox*^ mice (Fig. 7B, E, F). Since the activation of the mitogen-activated protein kinase (MAPK) pathway has been associated with oxidative stress-induced cell death [12], and phosphorylation of p38 and Erk are two classical markers of activated MAPK pathway, we examined p38 and Erk phosphorylation status. With aging, the levels of p38 and Erk phosphorylation in the cerebral cortex and hippocampus increased, while *Cp*^*Gfap*^*cKO* interfered with this process (Fig. 7B, E, F). In addition, recent studies have suggested that a process known as ferroptosis plays an important role in iron-induced cell death in an apoptosis-independent manner [28, 29]. We therefore examined the protein levels of ACSL4 and GPX4, the key regulators of ferroptosis. However, we did not observe any apparent changes in the expression of these two proteins between the 6 M *Cp*^*flox/flox*^, 18 M *Cp*^*flox/flox*^ and 18 M *Cp*^*Gfap*^*cKO* mice in either the cerebral cortex or the hippocampus (Fig. 7G-J). Taken together, our results suggest that the Erk/p38 MAPK signaling pathway mainly participates in the protective effects of *CP* knockout in astrocytes during aging.

To further explore the mechanisms responsible for the recovery of learning and memory function under decreased hippocampal and cortical iron in the older mice, we performed western blot and immunofluorescence staining analysis to quantify ROS levels, as ROS are often part and parcel of iron-induced apoptosis [12, 30]. We found that the levels of malondialdehyde (MDA; marker of oxidative stress) and ROS in the cerebral cortex and hippocampus were decreased after *Cp* knockout in astrocytes of aged *Cp*^*flox/flox*^ mice (Fig. 7K, L, N, O), while the amount of total superoxide dismutase (T-SOD) was increased (Fig. 7M, P). 4-Hydroxynonenal (4-HNE) is one of the products of the peroxidation of polyunsaturated fatty acids by ROS attack [31]. Using immunofluorescence staining, we observed a marked increase in the ratio of NeuN^+^4-HNE^+^ / NeuN^+^ with aging (Fig. 7Q-S). As with the results of the MDA and ROS experiments, disruption of CP in astrocytes decreased the ratio of NeuN^+^4-HNE^+^ / NeuN^+^ compared with that in the 18 M *Cp*^*flox/flox*^ mice (Fig. 7Q-S).

AD is the most common neurodegenerative disease, whose top risk factor is advancing age [32]. With normal aging in mice, the levels of S396 phosphorylation of Tau, a hallmark of AD, were increased in the cerebral cortex and hippocampus, while this did not occur to such an extent in the 18 M *Cp*^*Gfap*^*cKO* mice, where the level was nearly equal to that in the 6 M control group (Fig. 7T-V). We also investigated Aβ oligomers and soluble APP, two additional hallmark features of AD [33], using the anti-amyloid oligomers antibody in the hippocampus of old and young mice. The levels of Aβ oligomers and soluble APP were elevated significantly with aging but decreased when there was a lack of *Cp* in astrocytes during old age (Fig. 7W-X). Taken together, our results suggest that the iron-ROS-Erk/ p38/MAPK-apoptosis pathway mainly participates in the protective effects of *Cp* knockout in astrocytes during aging and that CP deficiency in astrocytes may delay or prevent the pathogenesis of neurodegenerative disease like AD.

## Discussion

The brain comprises neurons and glial cells. The most abundant type of glial cell is the astrocyte, which is important in maintaining the structure and function of the BBB, which also includes BMVECs and pericytes [34]. Astrocytes are likely responsible for the transport of iron from BMVECs into the neural network. Our previous report shows that neurons and other cells in the CNS only send signals of iron deprivation to the BMVECs. In response, the BMVECs release stored iron, which can then be absorbed and delivered by astrocytes [6].

BBB is the gateway for iron entry into the brain from the blood. Astrocytes surrounding the BBB are the main cells responsible for CP synthesis, and astrocyte-secreted soluble CP and astrocyte GPI-CP are thought to facilitate the export activity of BMVEC FPN1 by catalyzing the oxidation of ferrous iron (Fe^2+^) to ferric iron (Fe^3+^) [6]. The Fe^3+^ is then captured by transferrin in the interstitial fluid and distributed to glial cells and neurons in the CNS [4]. However, to the best of our knowledge, the specific consequences of astrocyte CP deficiency, with respect to the regulation of brain iron metabolism and function, has not been explored. As expected, the astrocytic conditional knockout of *Cp* revealed that astrocytic CP plays critical roles in iron transport from the blood into the brain via the BBB, since the brain iron contents were decreased in *Cp*^*Gfap*^*cKO* mice with iron retention in BMVECs. Thus, a deletion of astrocyte *Cp* blocked the iron export via FPN1 in BMVECs into the hippocampus and cerebral cortex. Our further experimental data demonstrated that iron levels were decreased in neurons and glial cells. HP is predominantly expressed in neurons [35, 36], where it participates in iron export; and overexpression of HP can increase iron efflux, leading to lower neuronal iron levels [36], while HP deletion in neurons led to neuronal iron accumulation [35]. Our results show an increased level of HP which further diminished the iron concentration in neurons, when astrocytes have disrupted *Cp*. Thus, the superposition decreased iron in the brain with enhanced of iron release from neurons via HP exacerbated the decrease of iron in neurons of *Cp*^*Gfap*^*cKO* mice.

Iron is essential for cell metabolism and the synthesis of neurotransmitter and DNA, which are important for myelination, cell differentiation and neurogenesis. Long-term iron deficiency during development likely causes abnormal recognition memory processing and spatial learning [37]. Therefore, iron is critical for normal functioning of the brain. In young *Cp*^*Gfap*^*cKO* mice with iron deficiency in the cerebral cortex and hippocampus, the learning and memory ability was impaired, while, strikingly, the cognitive function improved in aged *Cp*^*Gfap*^*cKO* mice upon brain iron depletion in these regions.

The formation of learning and memory requires a complete synaptic framework and active circuit plasticity [38]; learning and forgetting are associated with synaptic formation and elimination on axonal-dendritic overlaps [39]. Recent studies have shown that conditional knockout of *Cp* in oligodendrocytes induces iron overload, brain oxidative stress, myelin disruption and neurodegeneration in aged mice [40]. In our study, we examined the expression of SYN and PSD95, as well as synaptic structure. In young *Cp*^*Gfap*^*cKO* mice, we found that the level of SYN, the number of spines of dendrites and the density of synapses were all decreased in the cerebral cortex. We also found that, in the hippocampus, the level of PSD95, the number of spines of dendrites and the density of synapsis were similarly decreased. These results suggest that synaptic plasticity declined after deletion of *Cp* in astrocytes, likely causing cognitive dysfunction. Considering that hippocampal neurogenesis dysfunction is correlated with learning and memory impairment [22], we assessed the number of DCX-positive immature neurons and Ki67-positive neuronal precursors in the DG, and found they were all decreased in young *Cp*^*Gfap*^*cKO* mice. In addition, iron deficiency in the brain has been shown to associate with the delay of myelin development and behavioral impairment [41–43]; impairment of myelin development in the CNS can result in memory dysfunction [26]. The young *Cp*^*Gfap*^*cKO* mice also exhibited decreased MBP expression and a thinner axonal myelin sheath compared to control mice. In *Cp*^*Gfap*^*cKO* mice, the iron was deficient in neurons and oligodendrocytes in the cerebral cortex and hippocampus, which may lead to impaired development of synapses and myelination. It is also reported that copper deficiency was correlated with myelin alterations [44, 45], our results didn’t find changes of copper contents in cerebral cortex and hippocampus of young *Cp*^*Gfap*^*cKO* mice. Therefore, conditional knockout of *Cp* in astrocytes induced memory dysfunction was not related to copper alterations.

Iron is a co-factor for many enzymes and participates in the synthesis of proteins in the CNS. Tyrosine hydroxylase is the rate-limiting enzyme in dopamine [46] and NE [47] synthesis. Tryptophan hydroxylase, also contains an iron co-factor, which is required for 5-HT synthesis [48]. Our results demonstrate that NE and 5-HT levels were decreased in the brain in 6 M *Cp*^*Gfap*^*cKO* mice, suggesting that astrocytic *Cp* deletion-induced iron deficiency may interfere with neurotransmitter production in the brain, which might be another important cause of cognitive function decline.

We were surprised to find that learning and memory were improved under astrocyte CP deficiency in 18 M mice compared to 18 M *Cp*^*flox/flox*^ mice. Previous studies have shown that CP plays a key role in the prevention of neurodegenerative disease and free radical damage [2, 10, 12, 49]. But the question of how *Cp* conditional knockout in astrocytes have a beneficial effect on the learning and memory abilities of old mice remains unclear.

It is known that the total iron content of the brain increases during aging [50], and it is likely that excess iron may contribute to the pathology of AD. Previous studies have shown that iron accumulation is an important factor in neurodegenerative disease [4, 46]. It has been suggested that global *Cp* deletion may increase brain iron and contribute to neurodegenerative diseases such as AD and PD [11, 12, 51]. With aging, the iron status can be dangerously elevated in the CNS of aceruloplasminemic patients and *Cp*^-/-^ mice, leading to behavioral dysfunction, such as late cognitive impairment and cerebellar ataxia [3, 12, 52, 53]. However, we observed that the iron levels were decreased in the cerebral cortex and hippocampus of aged (18 M) *Cp*^*Gfap*^*cKO* mice compared to 18 M *Cp*^*flox/flox*^ mice, with the expression of HP increased. Iron accumulation with aging and neurodegenerative damage have been tightly associated with ROS generation and subsequent cell death [50]. Recent studies have rigorously reported that excess ROS induced by iron and Aβ can activate the MAPK pathway, leading to apoptosis [12, 30, 54]. These hallmarks of AD become increasingly apparent with aging [55]. Iron has also been identified as an exacerbating factor in Aβ deposition and Tau hyperphosphorylation, both of which are hallmark contributors to AD pathogenesis [50]. Mitochondrial ferritin overexpression in SH-SY5Y cells abrogates the neurotoxicity induced by Aβ via sequestration of iron into the mitochondrial storage protein [30]. Moreover, intranasal administration of deferoxamine has also been shown to attenuate the development of AD in APP/PS1 mice [56]. To investigate whether the improved cognitive function is related to ROS, apoptosis and/or the phosphorylation of tau protein, we examined their levels in old *Cp*^*Gfap*^*cKO* mice, and found a significant decrease in apoptosis accompanied with decreased oxidative stress, increased Bcl-2/Bax ratio, decreased tau phosphorylation and diminished activity of the Erk/p38 MAPK pathway, all of which are linked to a delay in the pathogenesis of AD.

Based on our working model in which astrocytic CP is required for iron translocation across the BBB, the deletion of *Cp* is expected to limit the amount of iron in the brain, which is contrary to the results of iron deposition in the brain of global *Cp* knockout mice [3, 11, 12, 40]. In order to exclude whether peripheral CP and iron levels have an effect on the decrease of brain iron in *Cp*^*Gfap*^*cKO* mice, we examined the CP protein levels and iron contents in the liver and blood in the *Cp*^*Gfap*^*cKO* mice and controls (Fig S7). The astrocytic *Cp* knockout did not affect the iron contents in peripheral tissues, supporting our conclusion that the above changes in cognitive function are due to disrupted CP activity in the brain, rather than any peripheral consequences.

Although it is not possible to draw a definitive conclusion about the mechanisms underlying why global CP deficiency causes brain iron overload, while the opposite occurs in astrocyte-specific CP deficiency, we speculate that iron accumulation in the brain of global *Cp*^*-/-*^ mice is possibly due to the following reasons: 1) liver iron deposition causes the release of inflammatory cytokines [57], and the latter increases the permeability of the BBB, which enhances iron entry into the brain; 2) aceruloplasminemia patients usually have very high levels of ferritin in the blood [58–61]—the increased ferritin can bind TfR1 of BMVECs and facilitate iron uptake into the brain [62, 63]; 3) it has been reported that CP can be expressed in neurons [64, 65]. Neuronal CP may play a role in neural iron efflux, while the expression of CP in glial cells also regulate iron metabolism in the brain. In the global *Cp*^*-/-*^ mice, the deficiency of neuronal CP may block neural iron export to induce brain iron overload.

**Fig. 8:**
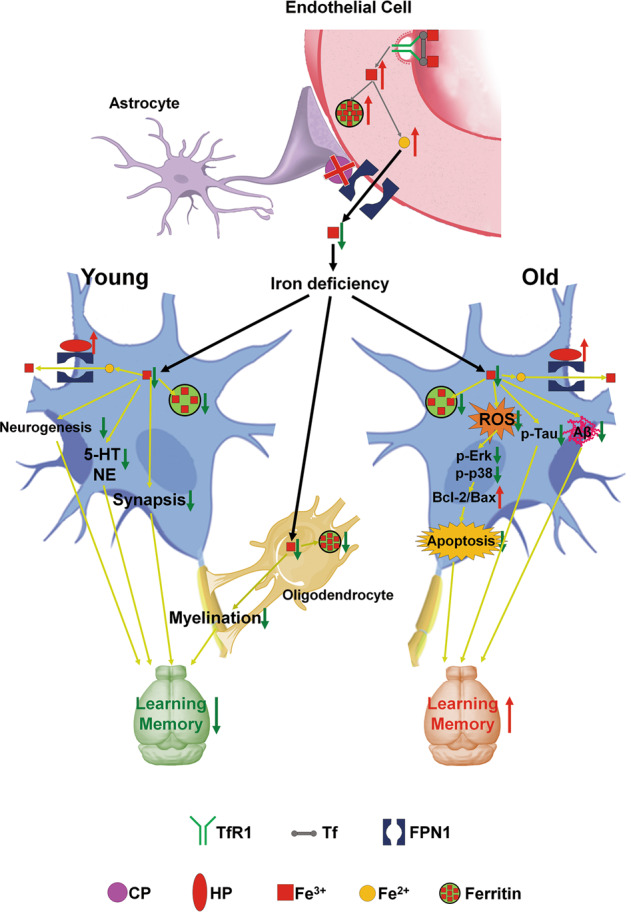
A schematic diagram of the proposed divergent effects of astrocyte-specific *Cp* knockout on learning and memory function. Astrocyte-specific *Cp* knockout blocks iron influx FPN1-CP pathway into the brain, leading to iron deficiency, decreased ferritin in neurons and oligodendrocytes, and increased iron levels in BMVECs of *Cp*^*Gfap*^*cKO* mice. Moreover, HP expression of neurons was up-regulated, enhancing the iron export from neurons and the iron deficiency of neuron. In young (6 M) *Cp*^*Gfap*^*cKO* mice, Iron deficiency can lead to abnormal adult hippocampal neurogenesis, synaptic formation, myelination and decreased 5-HT and NE synthesis. Finally, learning and memory dysfunction come into being. While in old (18 M) *Cp*^*Gfap*^*cKO* mice, iron accumulation with aging is attenuated. Thus, the level of oxidative stress is decreased, the MAPK (Erk/p38 phosphorylation, p-p38 and p-Erk) signaling activation is altered and the level of apoptosis is diminished. Besides, AD-like pathologies such as Aβ aggregation and Tau phosphorylation are also decreased, which may result from down-regulated iron contents. The decreased oxidative stress-induced apoptosis and hallmarks of AD may make the abilities of learning and memory improved at the old age.

In conclusion, deficiency of astrocyte CP led to a blockage of iron export via FPN1 from BMVECs into the cerebral cortex and hippocampus, with the iron remaining within in the endothelial cells, coupled with an increased expression of HP in neurons, ultimately causing cortical and hippocampal iron depletion. At a young age, the decreased iron contents may interfere with the synaptic formation, hippocampal neurogenesis, myelination and the synthesis of neurotransmitters, thus delaying learning and memory development. However, at old age, the decreased brain iron status prevents toxic iron accumulation and oxidative stress, which can alleviate MAPK-dependent apoptosis to improve spatial learning and memory (Fig. 8). Overall, our results indicate that CP in astrocytes is functions in iron influx into the CNS, rather than iron efflux, and that the consequences of CP disruption are widely divergent between young and old animals in regulating cognitive function of the brain. Our results provide potential new strategies of CP supplementation for the treatment of learning and memory impairment in the young and suppression of CP to delay senescence in learning and memory in the old.

## Materials and methods

### Animals

*Cp*^*flox/flox*^ mice on C57BL/6 genetic background were obtained by a genetic targeting strategy by the Beijing Vitalstar Biotechnology Company; the *Loxp* sites were inserted around exon 2 by homologous recombination (Fig S1); *Gfap-cre* mice on C57BL/6 genetic background were purchased from the Jackson Laboratory; *Cp*^*Gfap*^*cKO* mice were generated by crossing *Gfap-cre* mice with *Cp*^*flox/flox*^ mice. After knockout of exon 2 of the *Cp* gene by *Gfap-cre*, a frame shift mutation and premature stop codon leads to ceruloplasmin absence in astrocytes. All experiments were approved by the Animal Care and Use Committee of the Hebei Science and Technical Bureau, China, and by the Animal Ethics Committee of Hebei Normal University. All mice were housed at an ambient temperature of 21 ± 2 °C, and given free access to food and water under a 12 h light/dark cycle. 6 M or 18 M male mice were randomly assigned for the following experiments. The following PCR primer sequences were used for genotyping *Gfap-cre* (Forward-oIMR1084: 5’-GCGGTCTGGCAGTAAAAACTATC-3’, Reverse-*Cre*-R: 5’-CCTTCCAGGGCGCGAGTT GATAGCT-3’) and *Cp*^*flox/flox*^ (*Cp*-seq-forward: 5’-CAAAAAAGCCACTCAACAGCACC-3’, *Cp*-seq-reverse: 5’- CAGCTTGCAGTCCATTTAGTC-3’). The results and genotyping information can be found in Fig S1.

After neurobehavioral test as described as the following parts, mice were anaesthetized with 0.8% pentobarbital sodium, and then they received perfusion with pre-cooled saline to remove the blood until the fluid from the auricular sinus was clear. After that, sample preparation was performed according to the experimental requirements.

### Morris water maze test (MWM)

Spatial learning and memory function were assessed using the MWM, as described previously [66, 67]. The experimental apparatus was composed of a circular bucket (diameter = 120 cm, height = 50 cm) that was divided into four quadrants, filled with water, and maintained at 22 ± 2 °C. The mice were transferred to the behavior testing room to adapt for at least 30 min. The MWM test included five consecutive days of training trials, followed by one day of probe testing. The platform (diameter: 9.0 cm) was visible over the water surface by 1 cm during the training trials on the 1^st^ and 2^nd^ day, while the platform was hidden under the water surface by 0.5–1.0 cm on the 3^rd^ to 5^th^ day; mice were allowed to find the platform located in the first quadrant. The escape latency time was recorded for up to 90 s. The test was conducted four times a day for five consecutive days, with a rest period of at least 30 min between two adjacent tests. The position of the escape platform was constant. On the sixth day, the platform was removed and the mice were allowed to swim freely. The swimming locus of each mouse was recorded for 90 s with a camera connected to a computer. The number of times a mouse passed the platform location and the percentage of target quadrant passing time were recorded by the computer and used to evaluate the learning and memory ability of each *Cp*^*Gfap*^*cKO or Cp*^*flox/flox*^ mice.

### Contextual fear conditioning test

This test was performed following as previously described, with modifications [68]. Briefly, the test was conducted over 2 d. During the first day, the mice were placed in the test chamber and allowed to habituate for 3 min. Next, the mice were given three cycles of cued aversive stimuli (18 s, 5, 000 Hz, 75 dB)–shock (2 s, 0.5 mA) pairings, divided by an interval of 40 s (3 min total). The mice were then returned to their cages, 30 s after the last cycle. During the second day, the mice were tested and recorded in the training context for 3 min (context test) without presentation of the cued aversive stimulus. Two hours later, the mice were tested in a novel context, where the walls in the chamber were covered by inserts having different texture and color. The mice were placed in the chamber and habituated for 3 min, and then they received a continuous cue stimulus (75 dB noise) for 3 min (cued test). The behavior was recorded by a digital camera above the fear conditioning chamber and the percent freezing time was scored.

### Novel objects recognition (NOR)

The plastic chamber used in the open field test was also used in these tests. The mice were first accustomed to the apparatus for 5 min. Next, two objects were positioned 5 cm away from the two side walls. The mice were then placed in the apparatus and allowed to explore the two objects for 10 min. 24 h later, the mice were put in the same apparatus facing two objects, but one of the objects was replaced with a new object. The time spent exploring the novel object and familiar object was measured for 10 min.

### Inductively coupled plasma mass spectrometry (ICP-MS)

The total iron contents in the cerebral cortex, hippocampus and BMVECs were measured by ICP-MS as described previously [69]. Tissues were dried overnight at 105 °C, then 69% HNO_3_ was used for suspending the dried tissues at room temperature overnight. The tissues were then heated at 90 °C for 20 min and hydrogen peroxide (30%; Merck; equivalent volume of HNO_3_) was added for a further incubation at 70 °C for 15 min. The tissues were diluted in deionized water (≥18.2 MΩ) and detected by an ICP-MS spectrometer (Agilent 7700; Agilent Technologies, Santa Clara, CA, USA). The standards and digested samples were assessed at least in triplicate by ICP-MS.

### Western blot

For western blot analysis, the mice were anaesthetized and perfused with cold normal saline to remove the blood until the fluid from the auricular sinus was clear. After perfusion, the cerebral cortex and hippocampus were removed, and tissue protein extraction was performed using RIPA buffer (50.0 mM Tris-HCl pH 7.4, 150.0 mM NaCl, 1% NP40, 0.1% SDS) containing protease inhibitor cocktail tablets (68298, Roche Applied Science, Mannheim, Germany). The lysates were centrifuged at 12,000 *g* for 20 min and the supernatant was collected. Protein expression was assessed by western blot analysis as previously described [54, 70]. The primary antibodies used were the following: rabbit anti-mouse CP (1:5, 000; 21131-1-AP, Proteintech, USA), rabbit anti-mouse HP (1:10, 000; HEPH11-S, ADI, San Antonio, Texas, USA), rabbit anti-mouse SYN (1:400,000; ab32127, Abcam, SF, CA, USA), rabbit anti-mouse PSD95 (1:5, 000; GB11277, Servicebio, Wuhan, China), rabbit anti-mouse L-ferritin (1:5, 000; ab109373, Abcam), rabbit anti-mouse H-ferritin (1:10, 000; ab183781, Abcam), rabbit anti-mouse FPN1 (1:5, 000; MTP11-S, ADI), mouse anti-mouse TfR1 (1:3, 000; 13-6800, Invitrogen, Carlsbad, CA, USA), mouse anti-mouse β-actin (1:5, 000; CW0096, Cwbio Biotechnology limited company, Beijing, China), rabbit anti-mouse MBP (1: 10, 000; 10458-1-AP, Proteintech) and rabbit anti-mouse GALc (1:3, 000; 11991-1-AP, Proteintech), rabbit anti-mouse Bcl-2 (1:5, 000; GTX100064, GeneTex, SA, TX, USA), rabbit anti-mouse Bax (1:5, 000; #2772, Cell Signaling Technology, St. Louis, MA, USA), rabbit anti-mouse Phospho-p38 (p-p38) (1:1, 000) and p38 (1:1, 000; #4511 and #8690, Cell Signaling Technology), rabbit anti-mouse Phospho-Erk (1:5, 000) and Erk (1:5, 000; #4370 and #4695, Cell Signaling Technology), rabbit anti-mouse ACSL4 (1:5, 000; ab155282, Abcam), rabbit anti-mouse GPX4 (1:5, 000; ab125066, Abcam), rabbit anti-mouse p-Tau (S396) (1:100, 000; ab109390, Abcam) and rabbit anti-mouse A11 (1:5, 000; 200-401-E88, Rockland Immunochemicals, Inc., Philadelphia, PA, USA). After washing with TBS-T (pH 7.6, 137.0 mM NaCl, 0.05% Tween-20), the blots were incubated with an anti-rabbit secondary antibody- (1:5, 000; SA00001-2, Proteintech), or anti-mouse secondary antibody-conjugated horseradish peroxide (1:5, 000; SA00001-1, Proteintech) for 1.5 h at room temperature.

### Quantitative real-time PCR

Total RNA from cerebral cortex and hippocampus was extracted by TRIzol Reagent (Invitrogen) and complementary DNA (cDNA) was synthesized with a PrimeScript™ RT reagent Kit (RR037A, Beijing, China, Takara Biomedical Technology Co., Ltd.). Quantitative real-time PCR was performed using UltraSYBR Mixture (CW0957L, Cwbio Biotechnology limited company) and a Bio-Rad CFX 96 detection system (Bio-Rad). The mRNA levels were normalized to *β-actin* mRNA. The PCR primers used are as follows: *Cp*: Forward 5’- ACTGAAGCAGTCTGGGACTATGCT-3’, Reverse 5’- AGCCAGGCTGGTTTGTCTATAGTC -3’; *β-actin*: Forward 5’- AGGCCCAGAGCAAGAGAGGTA -3’, Reverse 5’- TCTCCATGTCGTCC CAGTTG -3’.

### Synchrotron radiation micro X-ray fluorescence (μ-XRF)

Mice were perfused with saline followed by 4% paraformaldehyde (PFA) in 0.1 M phosphate buffer (PB). The brain was removed, postfixed for 1.5 to 4 h and then soaked overnight in 30% sucrose until dropping to the bottom of the vessel. Serial coronal sections were cut at 50 μm intervals in a freezing microtome. Slices from the same coordinates were mounted onto Mylar film (polycarbonate). The brain sections were dried and put in a vacuum desiccator before analysis by μ-XRF. The experiment was performed on the 4W1B station at the Beijing Synchrotron Radiation Facility (BSRF). The continuous synchrotron X-rays were monochromatized by a Si (111) double crystal. The sample was placed at a 45° angle to the incident X-ray beam, and X-ray fluorescence was detected using a 50-mm^2^ silicon drift detector (Vortex, USA) oriented at a 90° angle to the incident beam. A light microscope was attached to a computer for sample viewing. A monochromatic SR with photon energy of 15 keV was used to excite the samples. The XRF signals were collected for up to 20 s (live time) at each point. In order to correct the effect of the SR beam flux variation on the signal intensity, the Fe peak areas were normalized to the current intensity (*I*_*0*_) in an ionization chamber, which was placed in front of the samples. The results were analyzed and mapped using Origin 8.0 software (Northampton, MA, USA).

### Immunfluorescence

The brains were removed, postfixed in 4% PFA and cryoprotected in 30% sucrose. Coronal sections were cut at a 15.0 μm thickness in a freezing microtome and antigen retrieval was performed in 10.0 mM citrate buffer (pH 6.0) in a microwave oven at 96–98 °C for 10 min. The samples were blocked with normal goat serum, rabbit serum or donkey serum, according to the primary antibodies, and incubated overnight at 4 °C with a primary antibody: anti-GFAP monoclonal antibody (1:500; MAB360, Millipore, Temecula, CA, USA), anti-CP antibody (1:500; 21131-1-AP, Proteintech), anti-HP (1:1, 000; HEPH11-S, ADI), anti-NeuN antibody (1:200; ab104224, Abcam), anti-Iba1 antibody (1:200; MABN92, Millipore), anti-CC1 antibody (1:300; ab16794, Abcam), anti-L-ferritin antibody (1:600; ab109373, Abcam), anti-H-ferritin antibody (1:600; ab183781, Abcam), anti-MBP antibody (1:500; 10458-1-AP, Proteintech), anti-DCX antibody (1:800, #4604, Cell Signaling Technology), anti-Ki67 antibody (1:500, ab15580, Abcam) and anti-4-HNE antibody (1:500; HNE11-S, ADI). The following secondary antibodies were used and the incubations were at 37 °C for 50 min: Dylight 488 goat anti-mouse IgG (1:200; RS23210, Immunoway, TX, USA) or Alexa Fluor 549 goat anti rabbit IgG (1:200; RS23320, Immunoway). Sections were washed with 0.01 M PBS (pH 7.2–7.4) after each step. Finally, the sections were photographed with an Olympus FV3000 microscope.

### Golgi staining

Golgi staining was examined by using the Frozen Section Neuron Golgi Staining Kit (Genmed Co., Shanghai, China). According to the manufacturer’s instructions, mouse brains were immersed in a solution consisting of Solutions A and B (1:1) for 14 d in the dark. Next, the brains were transferred to Solution C for at least 48 h at 4°C in the dark. The brains were then sectioned in a microtome (VT1000S, Leica, Wetzlar, Germany) to a thickness of 100 μm. Solutions D and E were used for staining the sections. Finally, the images of dendritic segments were scanned using an Axio Imager Z2 (Zeiss) attached to a tissue cytometer TissueFAXS (TissueGnostics, Vienna, Austria).

### Transmission electron microscopy (TEM)

Male mice (6 M) were perfused with saline, followed by fixation with 2.5% glutaraldehyde and 2% PFA in 0.1 M PB (pH 7.4) overnight at 4 °C, post-fixed with 1% osmic acid in 0.1 M PB (pH 7.4) for 2 h, dehydrated in acetone and embedded in araldite. Ultrathin sections (50 nm) were then obtained with an ultramicrotome (Ultracut R, Leica, Wetzlar, Germany), stained with uranyl acetate and lead citrate, and images were acquired using a transmission electron microscope (H-7650, Hitachi Ltd., Tokyo, Japan). The numbers of synapses in the cerebral cortex and hippocampus were counted and the G-ratio (inner diameter/outer diameter) of myelin were also calculated.

### TUNEL staining

Apoptosis in the cerebral cortex and hippocampus was determined using TUNEL method following the manufacturer’s protocol (TUNEL FITC Apoptosis Detection Kit [A111; Vazyme Biotech Co., Nanjing, China]). Nuclei were stained with DAPI. The number of TUNEL-DAPI-positive neurons was quantified as described previously [9]. The counting area was situated in the same position for the three groups.

### Isolation of BMVEC

After anesthetization with 0.8% pentobarbital sodium, the mice were perfused with pre-cooled saline and the cerebral cortex and hippocampus were immediately removed. The cerebral cortex and hippocampus were weighed and homogenized in cold HBSS (10.0 mM HEPES, 141.0 mM NaCl, 4.0 mM KCl, 1.0 mM MgSO_4_•7H_2_O, 1.0 mM NaH_2_PO_4_•2H_2_O, 2.5 mM CaCl_2_, 10.0 mM glucose and 1.0 mM sodium pyruvate) with Dextran-T70 (32%, Sigma) at a ratio of 1:3:4 (Brain tissues: Buffer: Dextran-T70). The homogenate was centrifuged at 7,450 *g* at 4 °C for 5 min. The white pellets were separated from the supernatant and homogenized again in 16% Dextran-T70 and centrifuged at 7,450 *g* at 4 °C for 5 min. The pellets were washed in the same buffer and centrifuged at 3, 500 *g*, at 4 °C for 5 min. The supernatants were removed and the BMVEC- containing pellet was retained for western blot analysis and ICP-MS.

### High performance liquid chromatography (HPLC)

The tissues were extracted and homogenized in ice-cold 0.1 M perchloric acid (v/w = 30:1) and immediately centrifuged at 15, 000 *g* for 15 min at 4 °C. The supernatant was collected and prepared for HPLC analysis or stored at -80 °C for later HPLC analysis with fluorometric detection. The column used was a REPROSIL-PUR 120 C18 5 μm (4.6 × 250.0 mm) from ThermoFisher. The mobile phase consisted of 0.05 M citric acid, 0.05 M sodium acetate and 0.5 mM sodium EDTA, 13% methanol, pH 3.1; the flow rate was 0.4 ml/min. The excitation wavelength used was 280 nm and the emission wavelength was 330 nm; the sample injection volume was 20 μL. The equipment included a Waters 717 Plus Autosampler System, a Waters 1525 Binary HPLC Pump and a Waters 2475 Multi λ Fluorescence Detector.

### Magnetic activated cell sorting (MACS^®^) separation

Astrocyte and neuron isolation was performed following the protocols of the Adult Brain Dissociation Kit (mouse and rat; 130-107-677, Miltenyi Biotec, Bergisch Gladbach, Germany). Briefly, adult mouse brains were dissociated by means of enzyme digestion and debris removal. Next, the astrocytes were further separated by using an Anti-ACSA-2 MicroBead Kit (Mouse; 130-097-678, Miltenyi Biotec, Bergisch Gladbach, Germany) and the neurons were further separated using a Neuron Isolation Kit (Mouse; 130-115-390, Miltenyi Biotec, Bergisch Gladbach, Germany). The number of cells was determined.

### Absolutely quantitative real-time PCR

The total RNA was extracted from the cells via a TaKaRa MiniBEST Universal RNA Extraction Kit (9767, Beijing, China, Takara Biomedical Technology Co., Ltd.). As a template, total RNA was used to produce cDNA using a PrimeScript^TM^ RT reagent Kit with gDNA Eraser (RR047A, Beijing, China, Takara Biomedical Technology Co., Ltd.). The cDNA was applied in real-time quantitative PCR by Premix Ex Taq™ (Probe qPCR; RR390A, Beijing, China, Takara Biomedical Technology Co., Ltd.). The number of mRNA copies was calculated by the Cq value between samples and a standard substance. The following PCR primers were used *Cp*: (Forward 5’-ACTGAAGCAGTCTGGGACTATGCT-3’, Taqman Probe: 5’-AAAGGTG CCATCTGTGTACTCAAAATAAAGGGCCTTCT-3’, Reverse 5’-AGCCAGGCTGGTTTGTCT ATAGTC -3’), *Heph* (Forward 5’-AGAGGATGTCGAGGGCTTCC-3’, Taqman Probe: 5’-ACC TGCCCAGGCTGGACATGTGCAA-3’, Reverse 5’-GTGCCAGGCCACAGTATCAC-3’).

### Measurement of MDA, ROS and SOD Levels

The levels of cortical or hippocampal MDA, ROS and total SOD (T-SOD) were measured as previously described [71]; the kits used were as follows: MDA assay kit (TBA method; A003-1, Nanjing Jiancheng Bioengineering Institute, Nanjing, China), ROS Assay Kit (S0033S, Beyotime Biotechnolgy, Shanghai, China), T-SOD assay kit (Hydroxylamine method; A001-1, Nanjing Jiancheng Bioengineering Institute). All experimental operations were performed according to the manufacturer’s instructions.

### Statistical analysis

Statistical analyses were performed using GraphPad Prism 9.0 Software. All the data are presented as the mean ± SEM. The statistical analysis of each group was assessed using a two-tailed Student’s t-test. The difference between two groups was determined by Student’s t-test. The data were considered statistically significant when the *p* value was less than 0.05.

## Supplementary information

Supplementary file (Fig S1-S7)

Original Data File

The reproducibility checklist

## Data Availability

Additional data can be found in the Supplementary materials. The remaining datasets and material generated in the study are available from the corresponding authors upon reasonable request.
