# Supplementary material for: The divergent effects of astrocyte ceruloplasmin on learning and memory function in young and old mice
Source: Cell Death Dis. 2022 Nov 28;13(11):1006. doi: 10.1038/s41419-022-05459-4 (PMC9705310; doi:10.1038/s41419-022-05459-4)
Supplement: Supplementary file 1 — Supplementary file (Fig S1-S7) [file 41419_2022_5459_MOESM1_ESM.docx]

**Supplementary Materials and Methods:**

**Elevated Plus Maze (EPM) Test**

The EPM apparatus comprised two closed arms (70 cm × 10 cm) and two open arms (70 cm × 10 cm). A central square area was connected to these arms at a height of 50 cm from the ground. At the beginning of the EPM test, the mice were placed on the central platform facing the open arms. The behavior of each mouse was measured for 5 min and recorded using a camera connected to a computer with Smart 3.0 software. The percentage of time and distance spent in the open arms was determined.

**Open Field Test**

The open field test was performed following a previously described procedure ^1^. Each mouse was placed in an open field plastic chamber (50 cm × 50 cm × 40 cm) and the behavior was recorded for 10 min using a camera and Smart 3.0 software as above. The percentage of time and distance passing the central area (30 cm × 30 cm) was recorded.

**Perl’s Staining**

In situ iron was detected with Perl’s staining as previously described ^2^. Briefly, the brain sections were incubated with 0.3% H_2_O_2_ in methanol for 30 min, and then incubated in freshly prepared Perl’s solution with equal amounts of aqueous potassium ferrocyanide (2%) and hydrochloric acid (2%) for 8 h at room temperature. Afterwards, the slides were washed with ddH_2_O and the staining was enhanced with DAB for 5 min. The slides were visualized by light microscopy (Axio Imager 2, Carl Zeiss).

**References**

1 . Leng L, Zhuang K, Liu Z, Huang C, Gao Y, Chen G, et al. Menin deficiency Leads to depressive-like behaviors in mice by modulating astrocyte-mediated neuroinflammation. *Neuron* 2018; 100: 1–13.

2 . Xu Y, Zhang Y, Zhang JH, Han K, Zhang X, Bai X, et al. Astrocyte hepcidin ameliorates neuronal loss through attenuating brain iron deposition and oxidative stress in APP/PS1 mice. *Free Radic Biol Med* 2020; 158: 84–95.

**
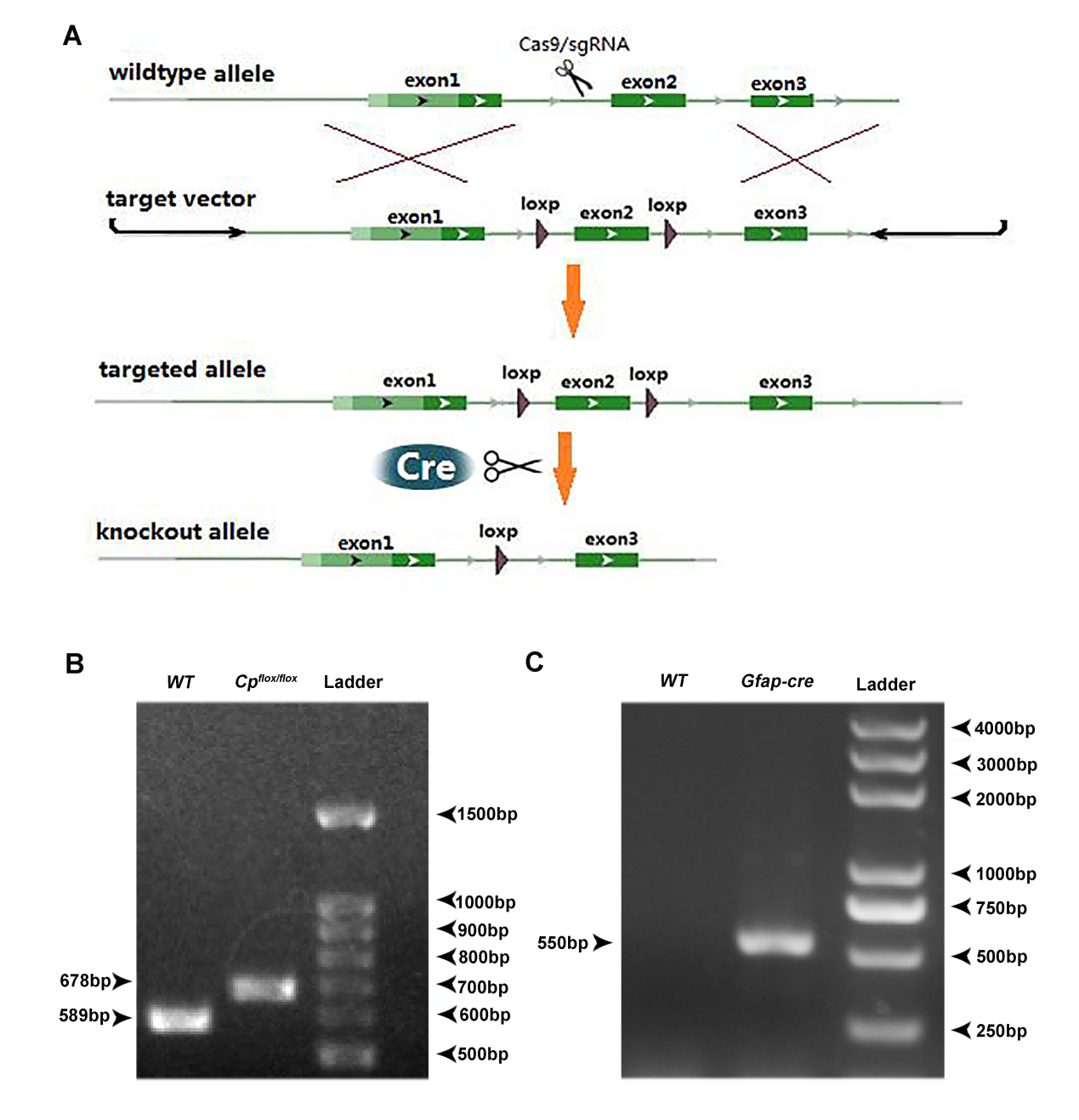
Supplementary Figures and Figure Legends:**

**Supplemental Figure 1. Generation and genotyping of astrocyte CP conditional knockout (*Cp^Gfap^cKO*) mice.**

**A:** Schematic illustration of the *Cre-loxp* system-induced conditional knockout of the *Cp* gene. Two *Loxp* sequences were inserted into both ends of exon 2, and Cre recombinase was able to recognize the loxp sites and delete exon 2 of the gene, resulting in CP ablation from astrocytes. **B:** Identification of the flox sequences inserted into the *Cp* gene (*Cp^flox/flox^*). PCR products of 589 bp represent wild-type (*WT*) mice and 678 bp products represent the *Cp^flox/flox^* genotype, as decribed in the Materials and Methods. **C:** Identification of the *Gfap-cre* genotype. PCR products with 550 bp indicate *Gfap-cre* transgenic mice. PCR products of both 678 bp and 550 bp identify the genotype of *Cp^Gfap^cKO* mice.


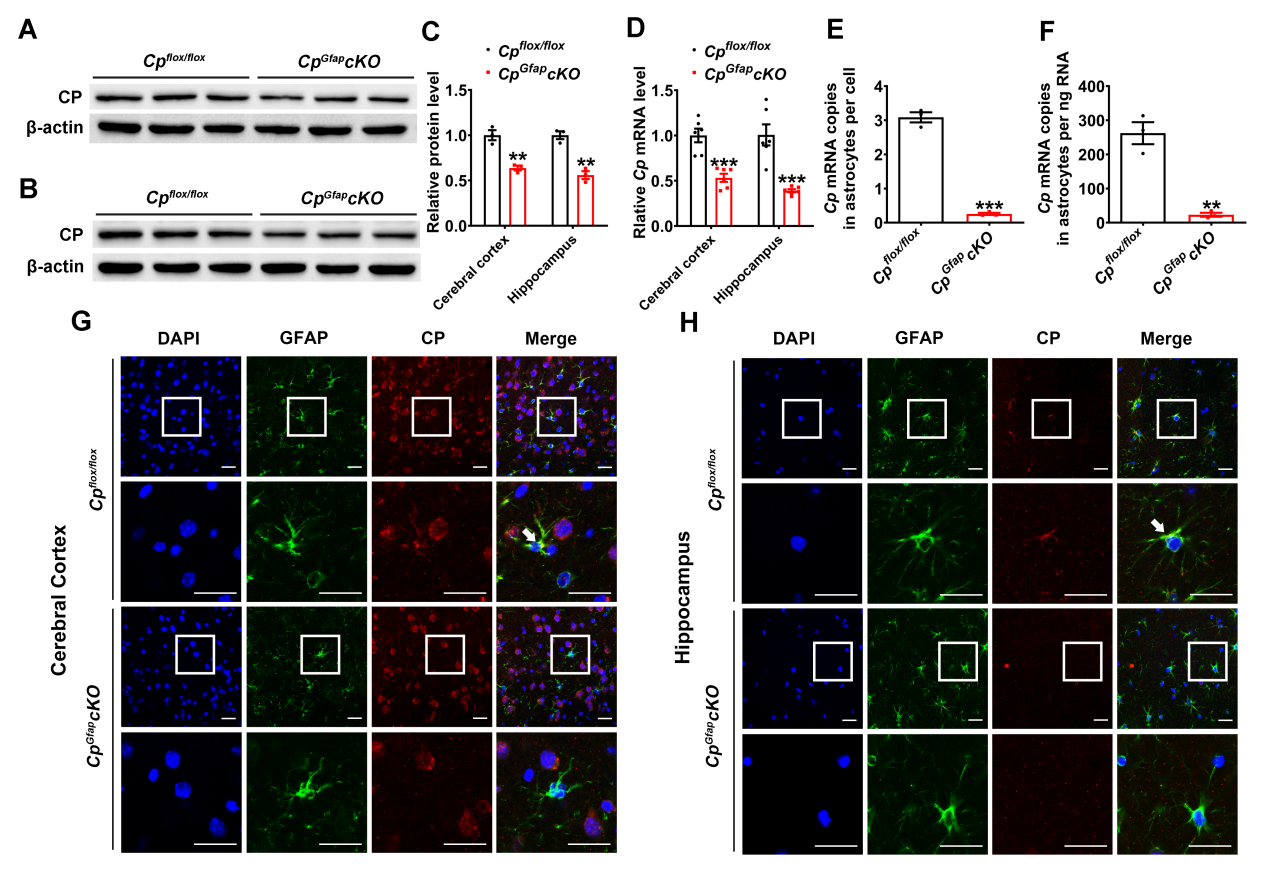


**Supplemental Figure 2. Identification of CP deficiency in astrocytes of *Cp* conditional knockout mice.**

**A** and **B** show the CP protein levels in both the cerebral cortex and hippocampus of *Cp^flox/flox^* and *Cp^Gfap^cKO* mice, as detected by western blot analysis, and the corresponding quantification of the immunostained bands is shown in **C**. (n = 3). **D:** *Cp* mRNA levels in the cerebral cortex and hippocampus of *Cp^flox/flox^* and *Cp^Gfap^cKO* mice, as measured by qPCR. (n = 6). **E** and **F** indicate the *Cp* mRNA levels in isolated astrocytes using an Anti-ACSA-2 MicroBeads Kit. The number of copies of *Cp* mRNA in one cell **(E)** and in 1 ng RNA **(F)** were quantified. (n = 3). **G** and **H** show the images from double immunofluorescence staining of CP (red) and GFAP (green, astrocyte marker) in the cerebral cortex **(G)** and hippocampus **(H)** of *Cp^flox/flox^* and *Cp^Gfap^cKO* mice. DAPI was used for nuclear staining. Scale bar: 25 μm. All data are presented as the mean ± SEM; **p < 0.01, ***p < 0.001 vs. *Cp^flox/flox^*.


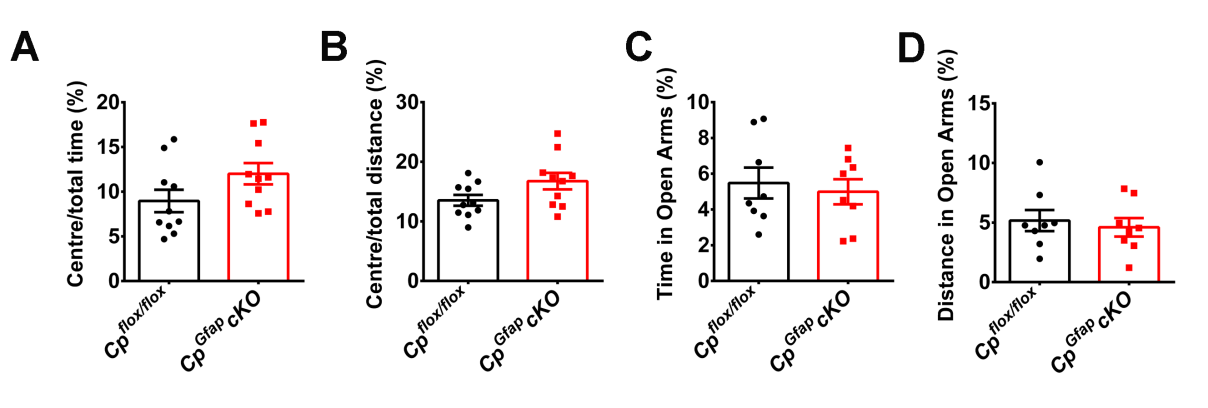


**Supplemental Figure 3. Anxiety-like behaviors were not affected by astrocytic CP conditional knockout.** Anxiety was assessed in the Open Field test; the percentage of time **(A)** and distance **(B)** spent in the center area were statistically analyzed. (n = 10; mean ± SEM; Student’s t-test). Anxiety-like behavior was also evaluated in an Elevated Plus Maze; the percentage of time **(C)** and distance **(D)** traveled in the open arm (n = 8, mean ± SEM, Student’s t-test) were recorded and are shown in the bar graphs.


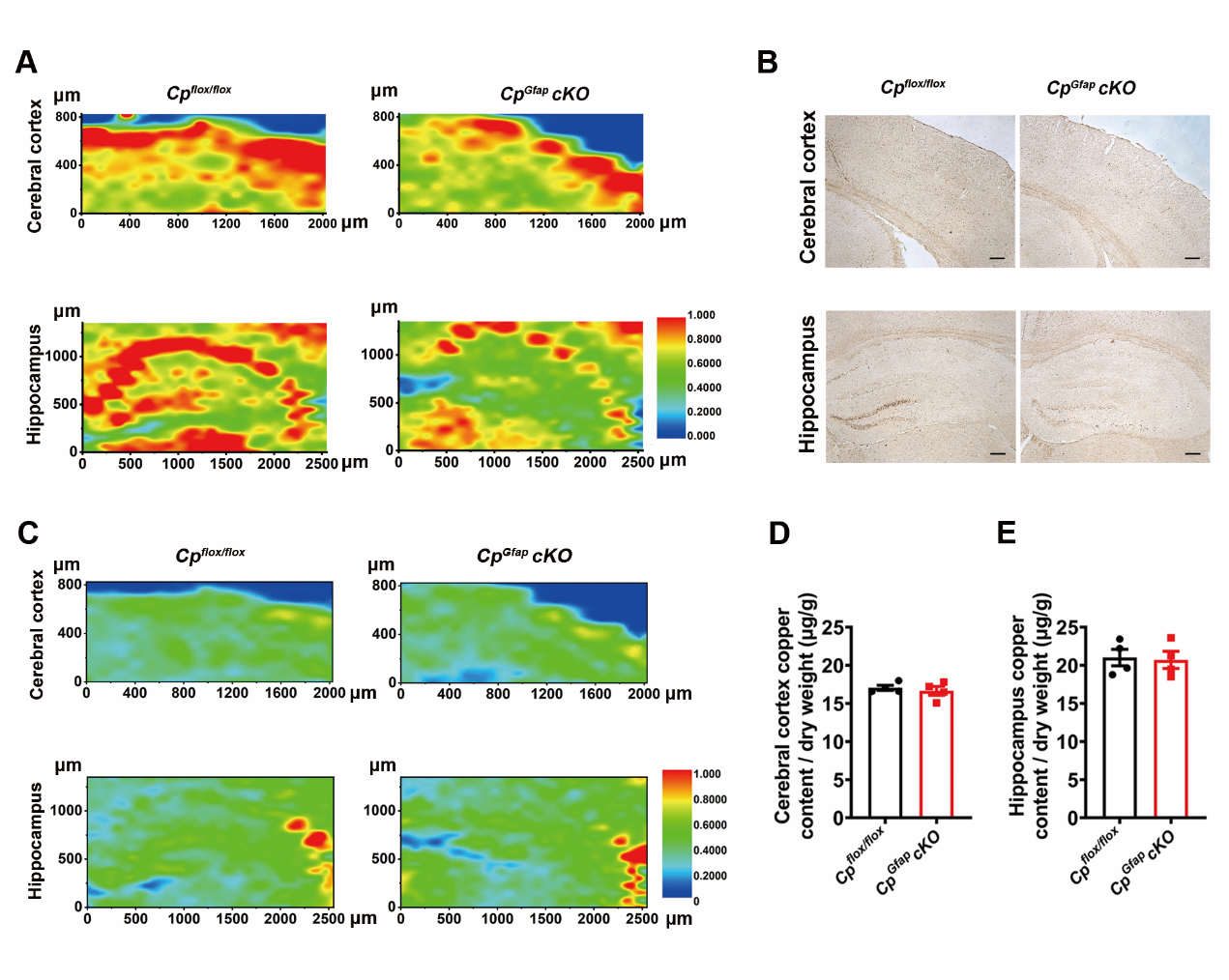


**Supplemental Figure 4. Effects of astrocytic *Cp* knockout on contents of iron and copper in the cerebral cortex and hippocampus. A:** Contents and distribution of iron was changed in the cerebral cortex and hippocampus of *Cp^flox/flox^* and *Cp^Gfap^cKO* mice detected by μ-XRF. Four representative images show the distribution and relative contents of iron in the brain sections. Different colors show different levels of iron in the brain. Red indicates a high level of iron while blue indicates low levels. **B:** Perl’s staining of iron in the cerebral cortex and hippocampus of *Cp^flox/flox^* and *Cp^Gfap^cKO* mice; Scale bar: 200 μm. **C:** Distribution of copper was detected by μ-XRF in the cerebral cortex and hippocampus of *Cp^flox/flox^* and *Cp^Gfap^cKO* mice. Red indicates a high level of copper while blue indicates low levels. **D** and **E** respectively show the copper contents in cerebral cortex and hippocampus detected with ICP-MS (n = 4).


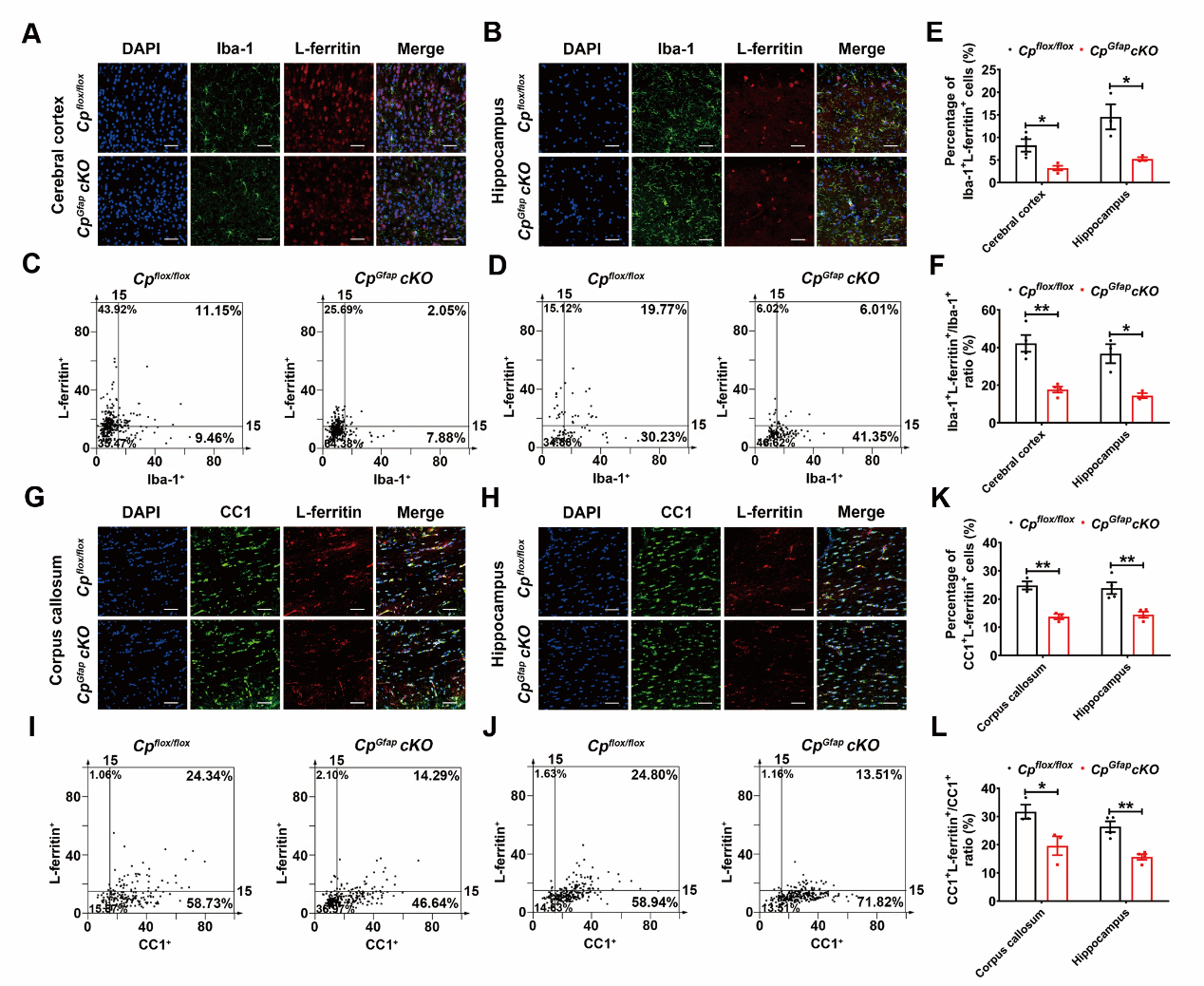


**Supplemental Figure 5. Ferritin in microglia and oligodendrocytes in *Cp^Gfap^cKO* mice.**

**A-F:** Double immunofluorescence labeling of L-ferritin and Iba-1 in the cerebral cortex **(A)** and hippocampus **(B)** of *Cp^flox/flox^* and *Cp^Gfap^cKO* mice. The intensity of Iba-1^+^ and L-ferritin^+^ cells in the cerebral cortex **(C)** and hippocampus **(D)** is shown in scatter diagrams. Quantification of the percentage of the Iba-1^+^L-ferritin^+^ cells **(E)** and the ratio of Iba-1^+^L-ferritin^+^ / Iba-1^+^ **(F)** are presented in the histogram plots. (n = 4 in cerebral cortex and n = 3 in hippocampus). **G-L:** Double immunofluorescence labeling of L-ferritin and CC1 in the corpus callosum **(G)** and hippocampus **(H)** of *Cp^flox/flox^* and *Cp^Gfap^cKO* mice. The intensity of CC1^+^ and L-ferritin^+^ cells in the corpus callosum **(I)** and hippocampus **(J)** is shown in scatter diagrams. Quantification of the percentage of the CC1^+^L-ferritin^+^ cells **(K)** and the ratio of CC1^+^L-ferritin^+^ / CC1^+^ **(L)** are presented in the histogram plots. (n = 3 in corpus callosum and n = 4 in hippocampus). DAPI was used for nuclear staining. Scale bar: 50 μm.­­ All data are presented as the mean ± SEM; *p < 0.05, **p < 0.01 vs. *Cp^flox/flox^*.


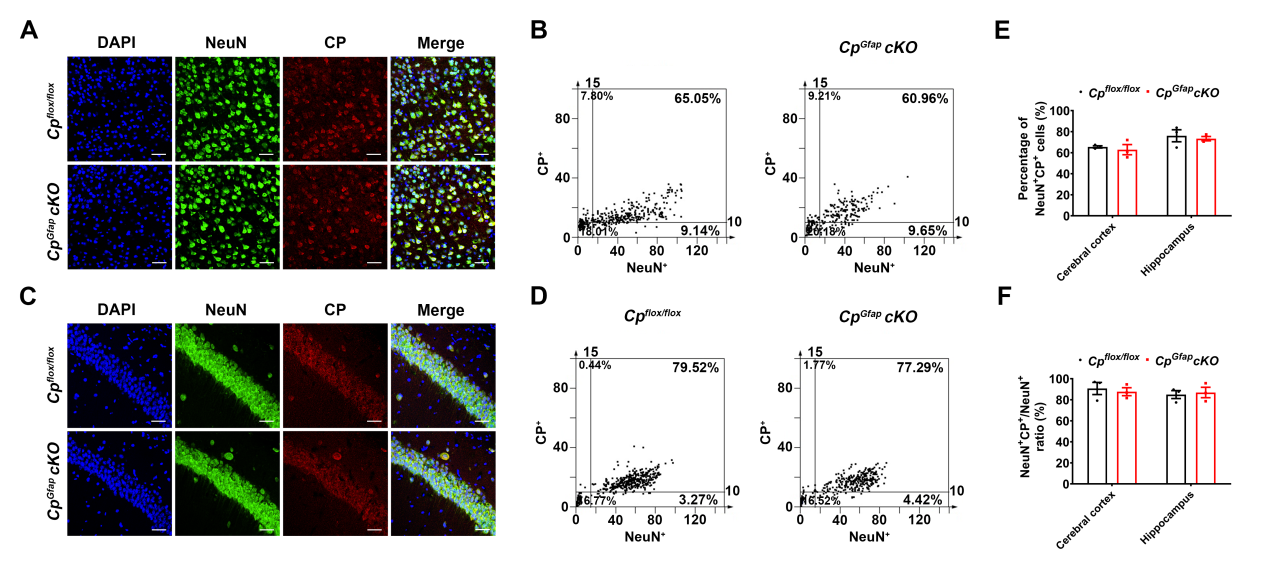


**Supplemental Figure 6 The levels of CP in neurons of *Cp^Gfap^cKO* mice.**

**A, C** Double immunofluorescence labeling of CP and NeuN in the cerebral cortex **(A)** and hippocampus **(C)** in *Cp^flox/flox^* and *Cp^Gfap^cKO* mice.

**B, D** The intensity of NeuN^+^ and CP^+^ cells in the cerebral cortex **(B)** and hippocampus **(D)** is shown in scatter diagrams.

**E, F** Quantification of the percentage of NeuN^+^CP^+^ cells **(E)** and the ratio of NeuN^+^CP^+^ / NeuN^+^ **(F)** are shown. (n = 3 fields from 3 mice per group; mean ± SEM; Student’s *t*-test). DAPI was used for nuclear staining. Scale bar: 50 μm.


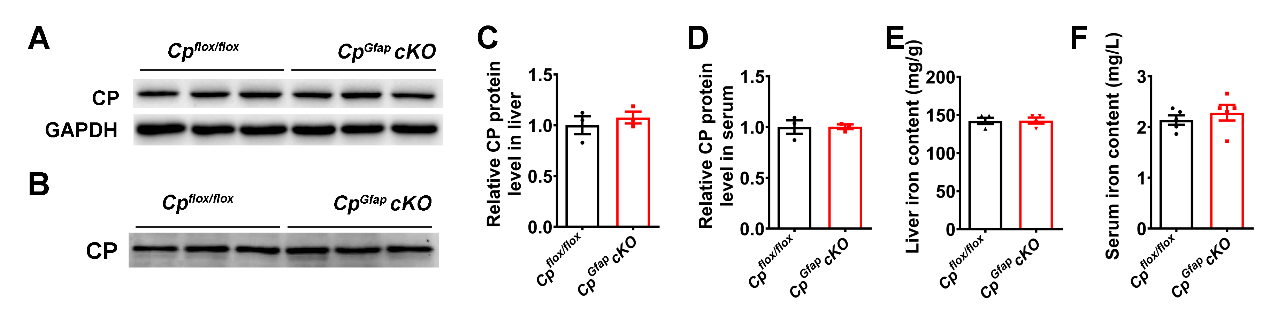


**Supplemental Figure 7. Astrocyte-specific CP ablation did not affect iron contents in liver and serum.**

**A, B** CP protein levels in the liver **(A)** and serum **(B)** of *Cp^flox/flox^* and *Cp^Gfap^cKO* mice were shown by Western blot, and the quantification of the western blot was shown in **C** and **D** (n = 3 per group; Mean ± SEM; Student’s *t*-test). **E, F** The iron contents in the liver **(E)** and serum **(F)** were detected in *Cp^flox/flox^* and *Cp^Gfap^cKO* groups by using kits which can measure the iron contents. There were no differences among these four groups (n = 4 per group in **E** and n = 5 per group in **F**; Mean ± SEM).
