# Supplementary material for: The divergent effects of astrocyte ceruloplasmin on learning and memory function in young and old mice
Source: Cell Death Dis. 2022 Nov 28;13(11):1006. doi: 10.1038/s41419-022-05459-4 (PMC9705310; doi:10.1038/s41419-022-05459-4)

Figure 1G

From left to right: *Cp*$^{fl/fl}$ *1*, *Cp*$^{fl/fl}$ *2*, *Cp*$^{fl/fl}$ 3, *Cp*^{Gfap}*cKO* 1, *Cp*^{Gfap}*cKO* 2, *Cp*^{Gfap}*cKO* 3

PSD95

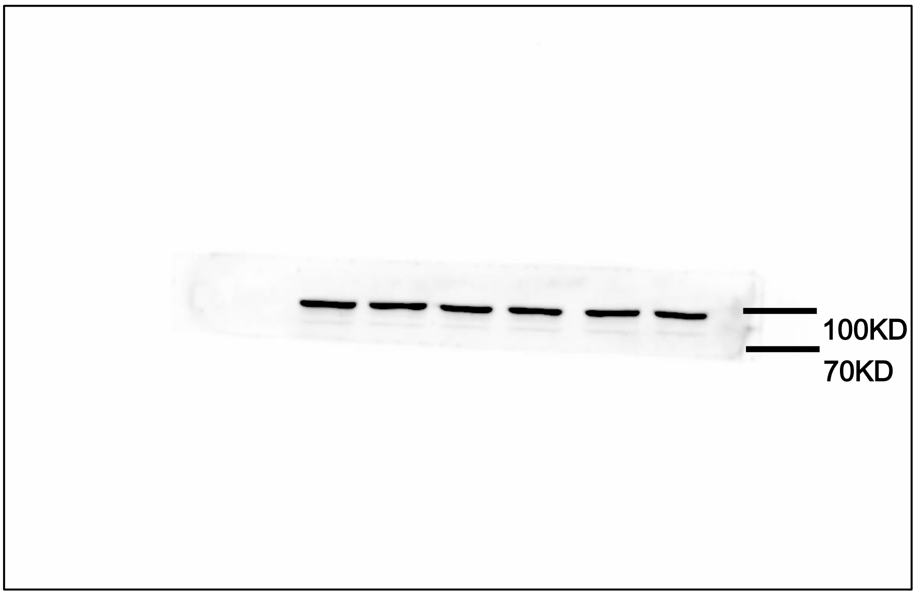

β-actin

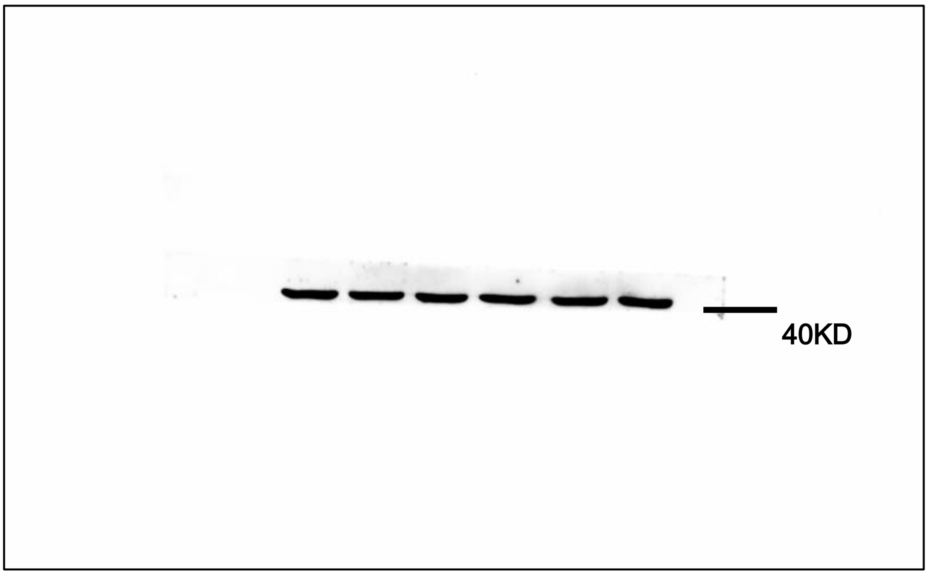

SYN

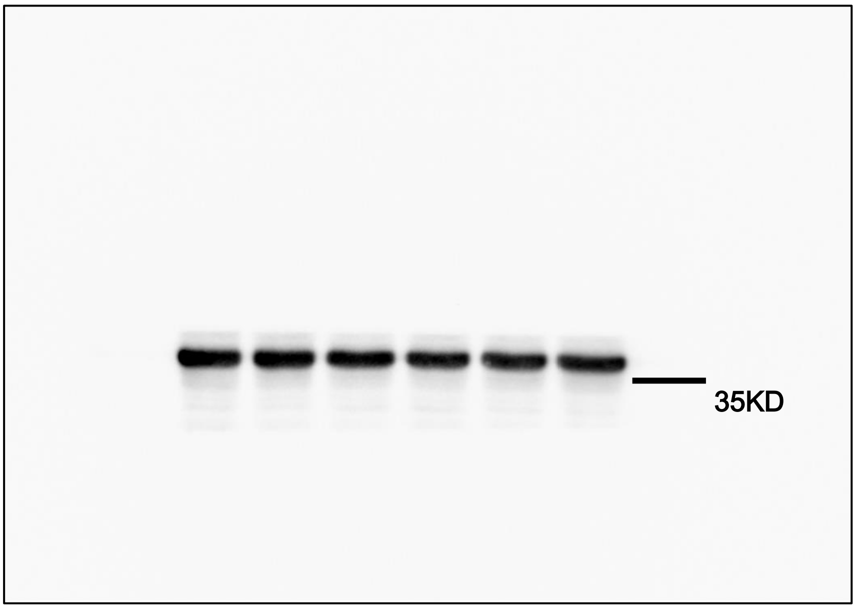

β-actin

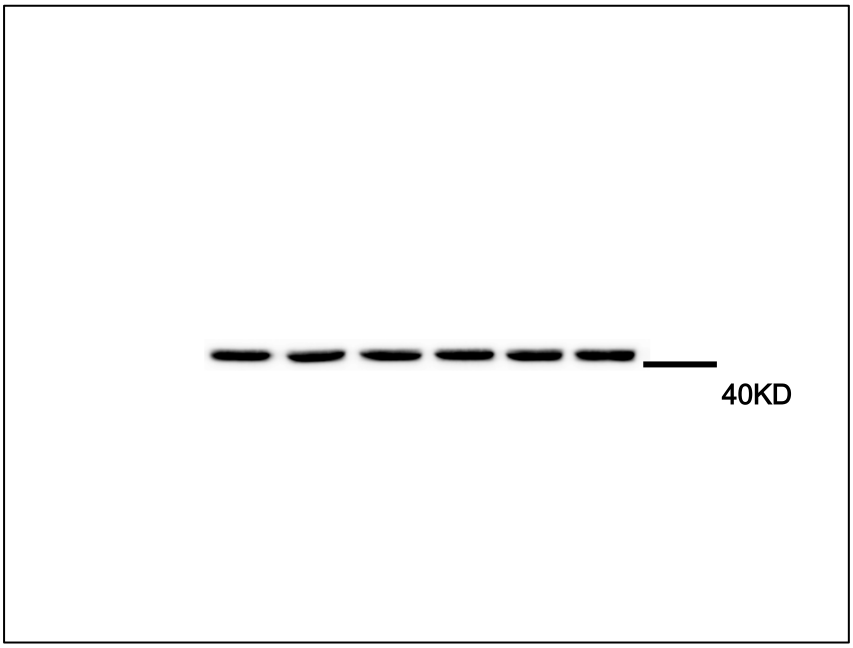

Figure 1I

From left to right: *Cp*$^{fl/fl}$ *1*, *Cp*$^{fl/fl}$ *2*, *Cp*$^{fl/fl}$ 3, *Cp*^{Gfap}*cKO* 1, *Cp*^{Gfap}*cKO* 2, *Cp*^{Gfap}*cKO* 3

PSD95

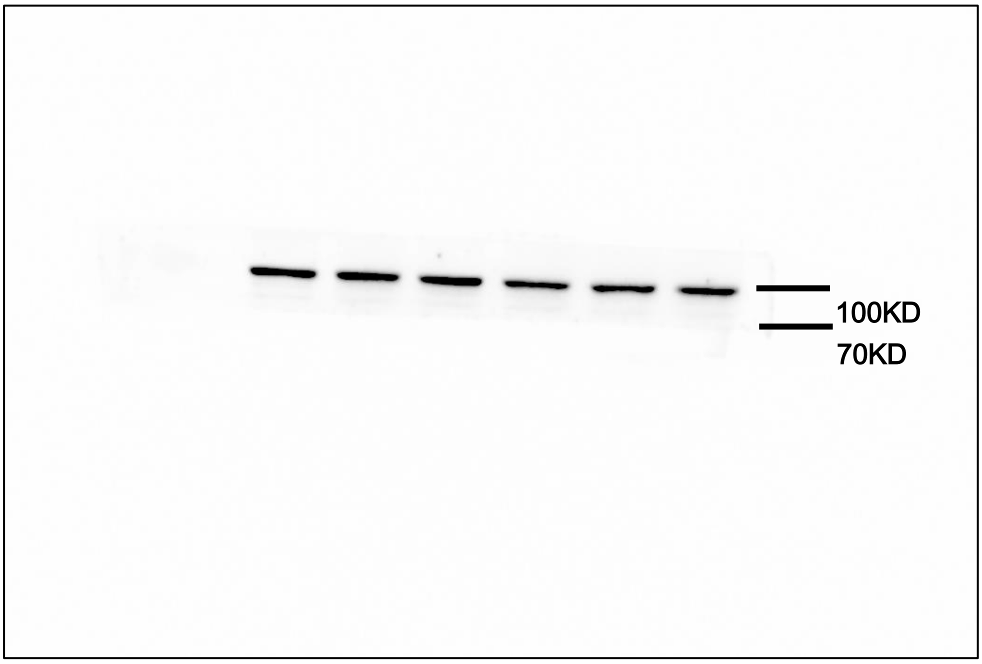

β-actin

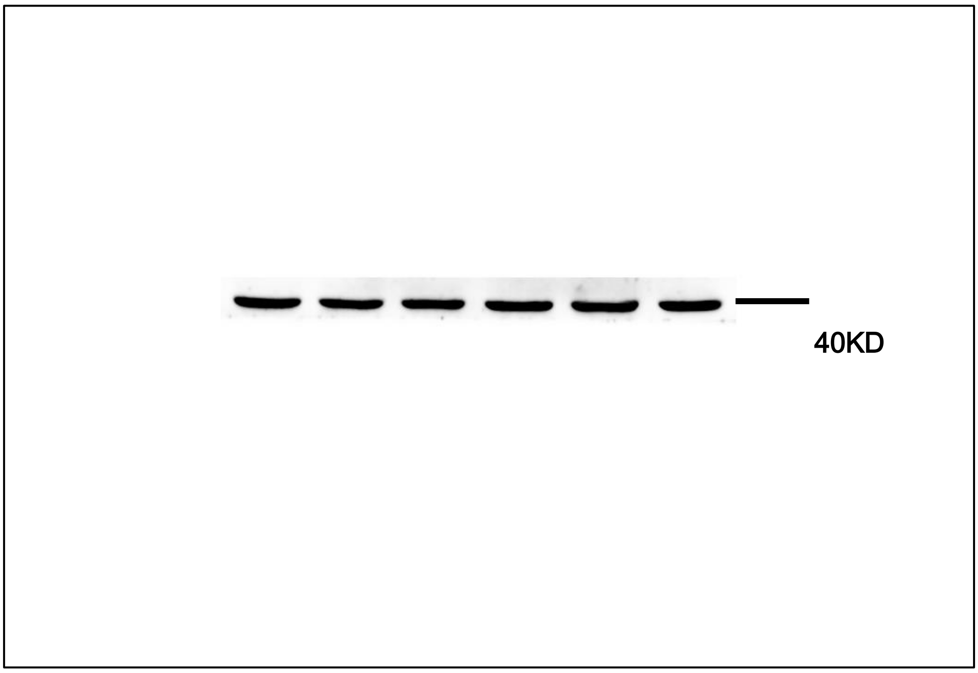

SYN

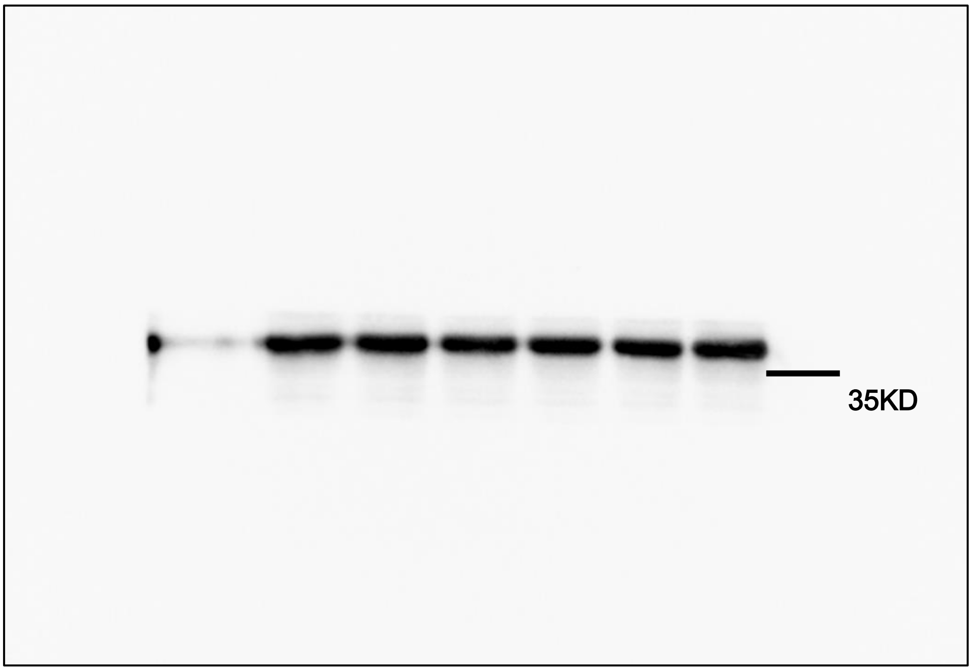

β-actin

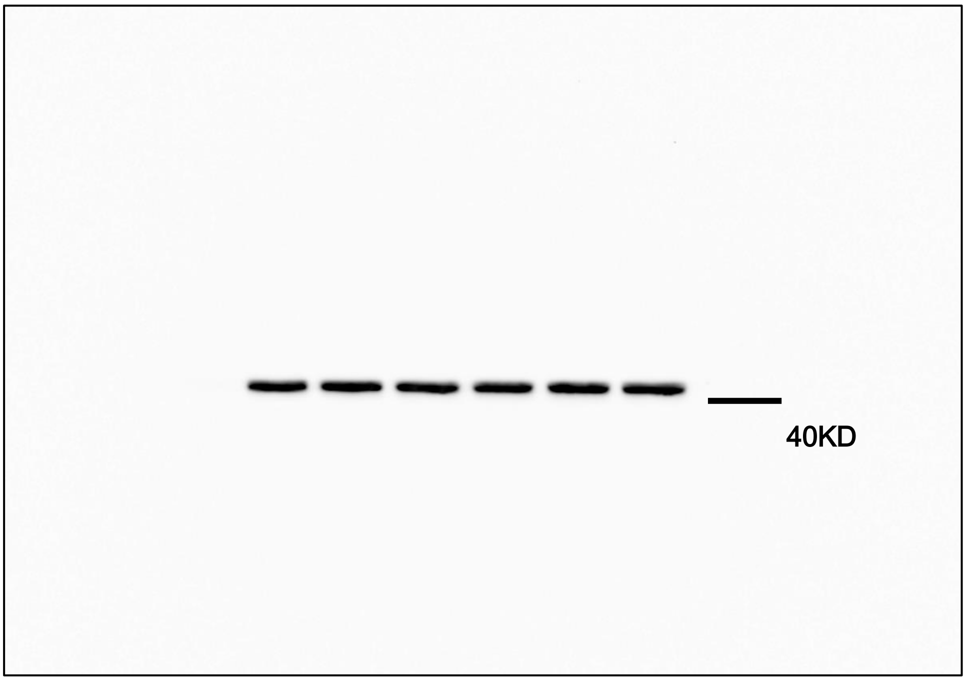

Figure 2A

From left to right: *Cp*$^{fl/fl}$ *1*, *Cp*$^{fl/fl}$ *2*, *Cp*$^{fl/fl}$ 3, *Cp*^{Gfap}*cKO* 1, *Cp*^{Gfap}*cKO* 2, *Cp*^{Gfap}*cKO* 3

GALc

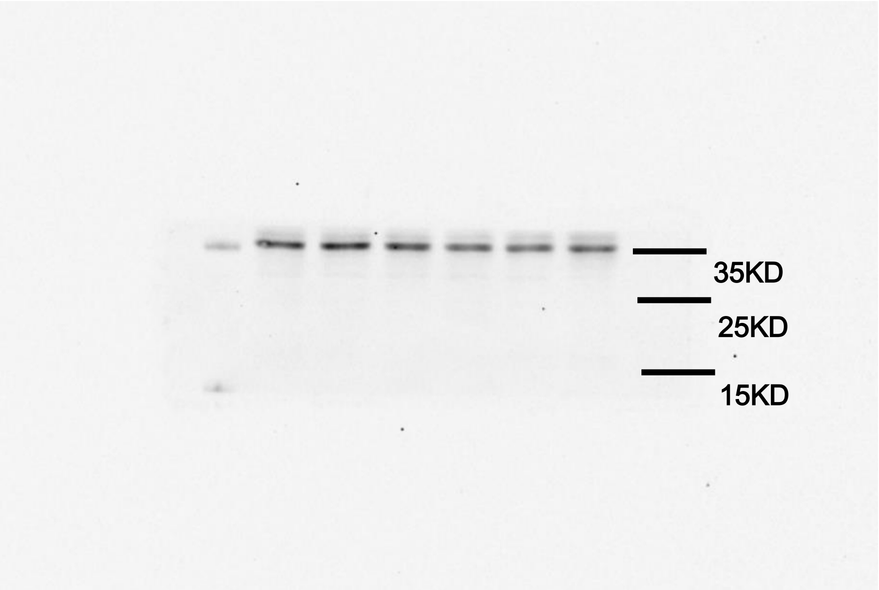

β-actin

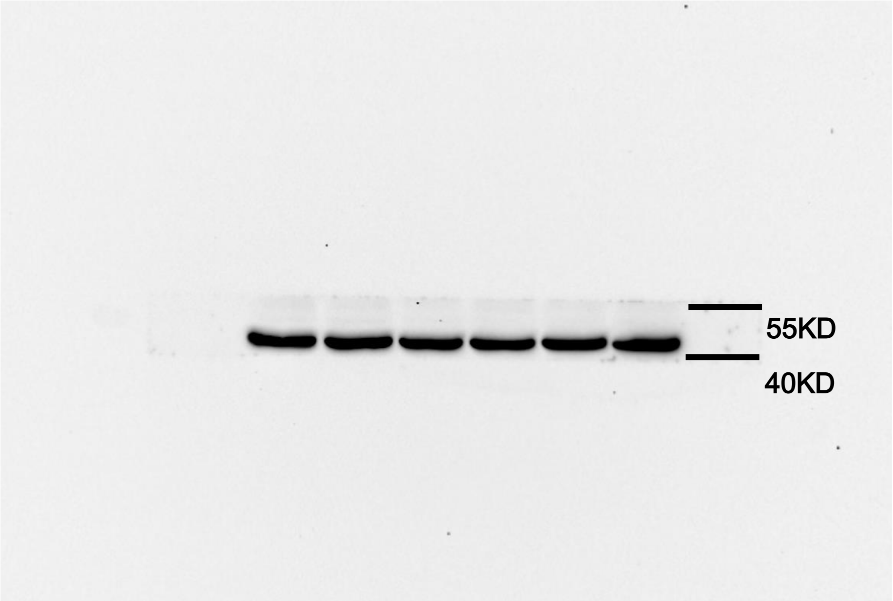

MBP

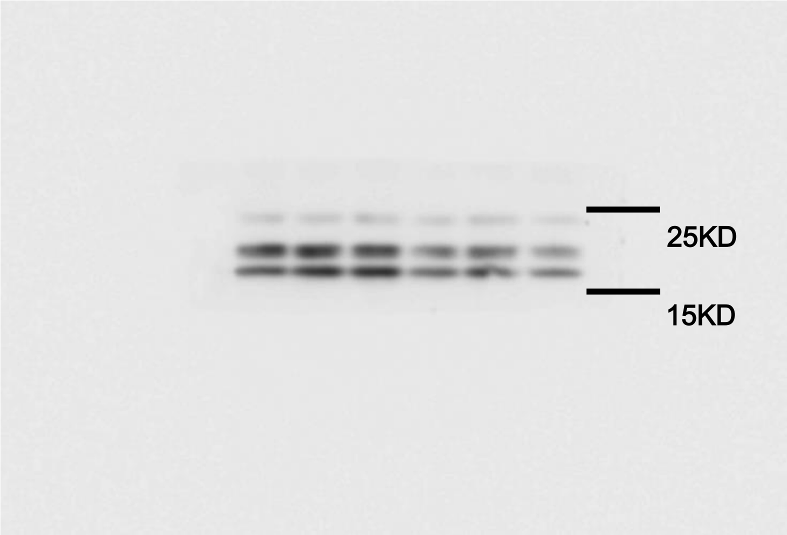

β-actin

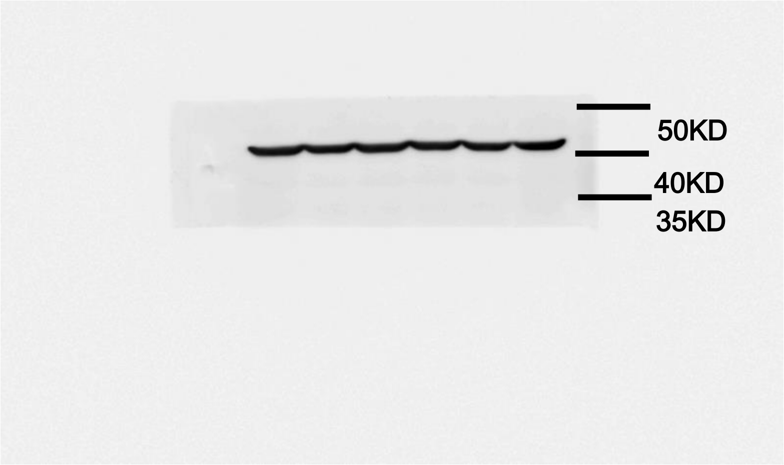

Figure 2C

From left to right: *Cp*$^{fl/fl}$ *1*, *Cp*$^{fl/fl}$ *2*, *Cp*$^{fl/fl}$ 3, *Cp*^{Gfap}*cKO* 1, *Cp*^{Gfap}*cKO* 2, *Cp*^{Gfap}*cKO* 3

GALc

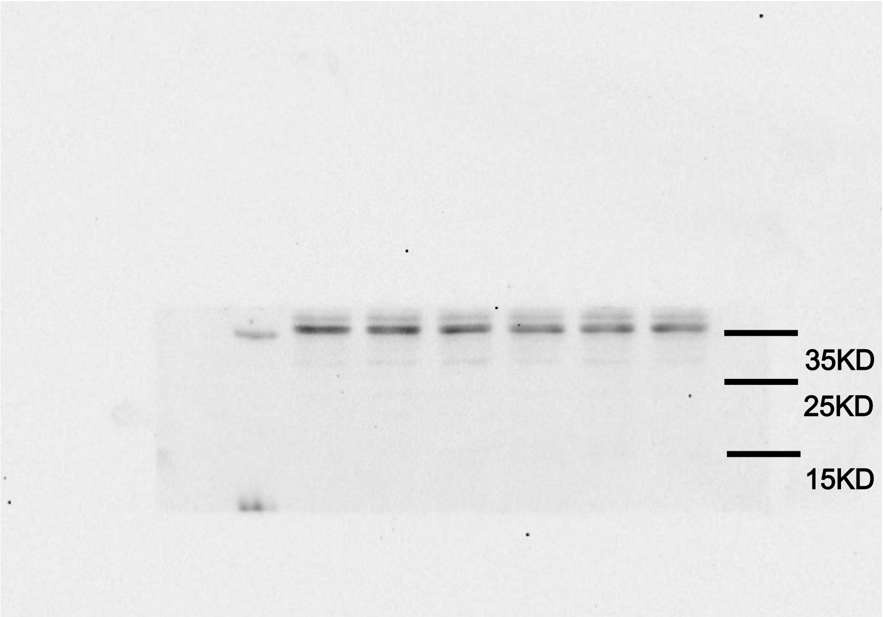

β-actin

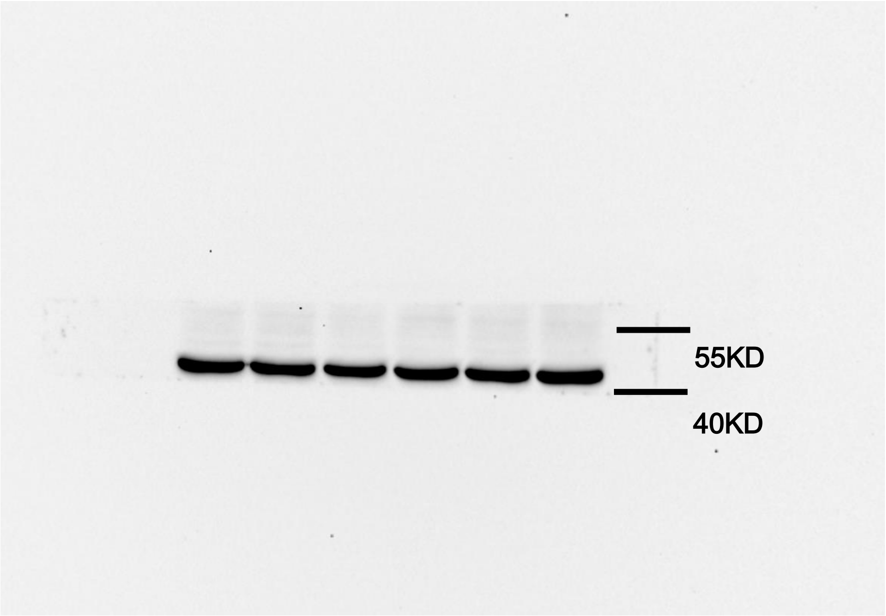

MBP

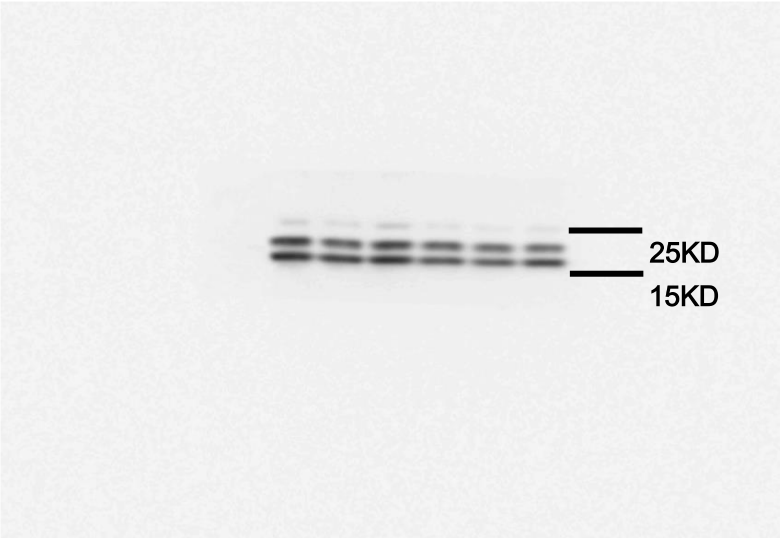

β-actin

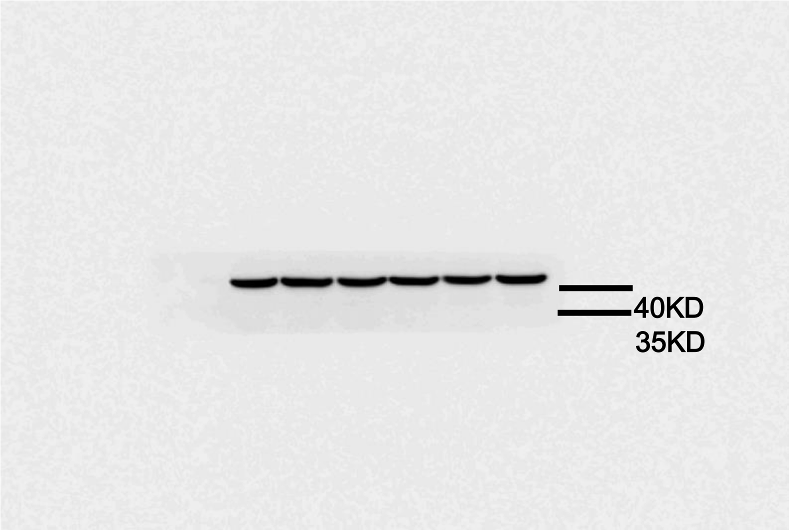

Figure 3A From left to right: *Cp*$^{fl/fl}$ *1*, *Cp*$^{fl/fl}$ *2*, *Cp*$^{fl/fl}$ 3, *Cp*^{Gfap}*cKO* 1, *Cp*^{Gfap}*cKO* 2, *Cp*^{Gfap}*cKO* 3

L-ferritin

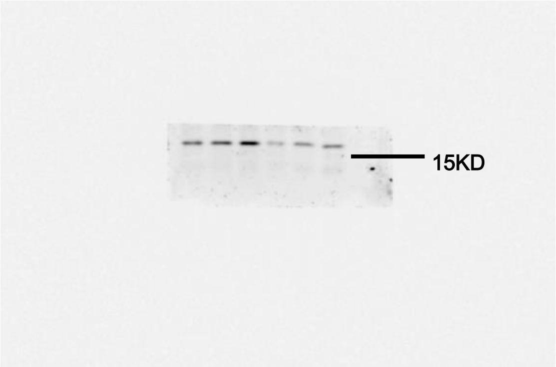

β-actin

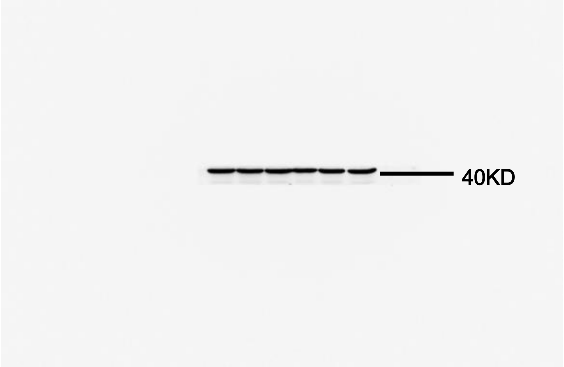

H-ferritin

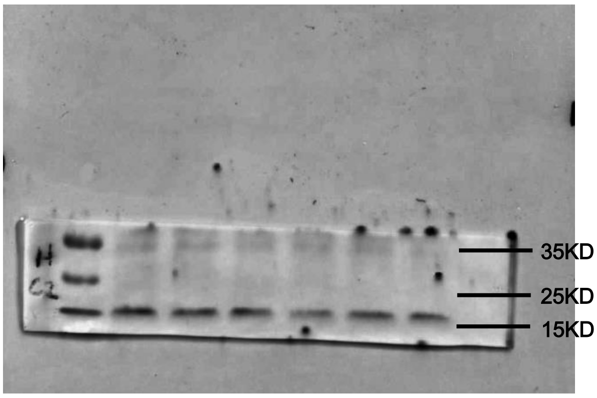

β-actin

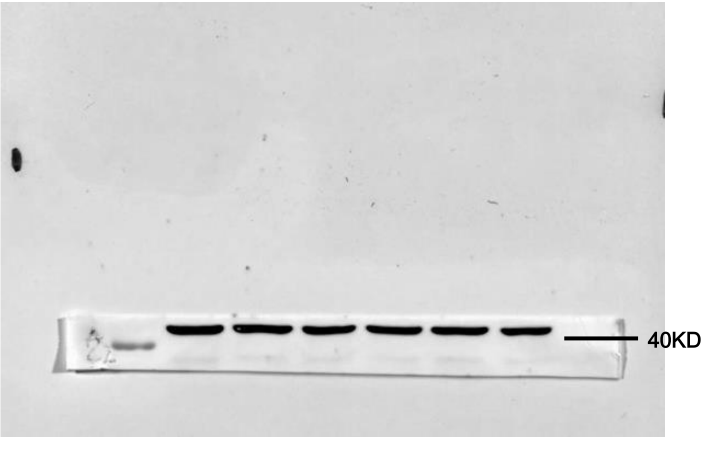

Figure 3C From left to right: $Cp^{fl/fl}$ *1*, $Cp^{fl/fl}$ *2*, $Cp^{fl/fl}$ 3, Cp^{Gfap}*cKO* 1, Cp^{Gfap}*cKO* 2, Cp^{Gfap}*cKO* 3

L-ferritin

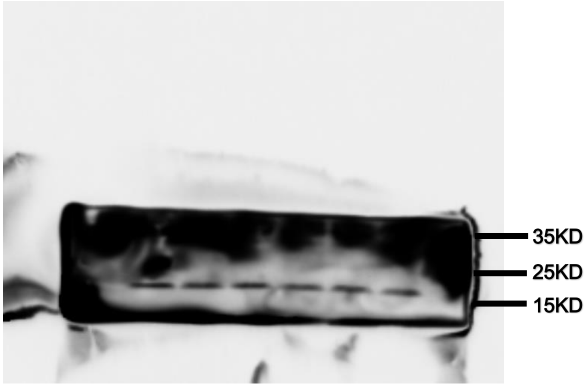

β-actin

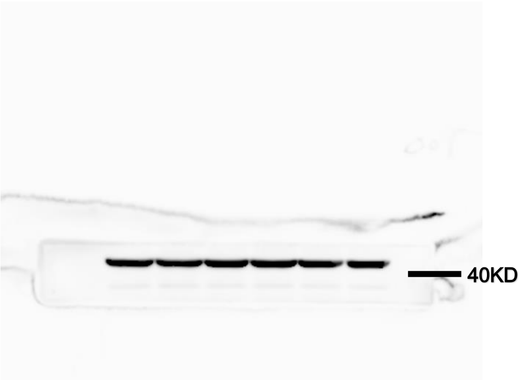

H-ferritin

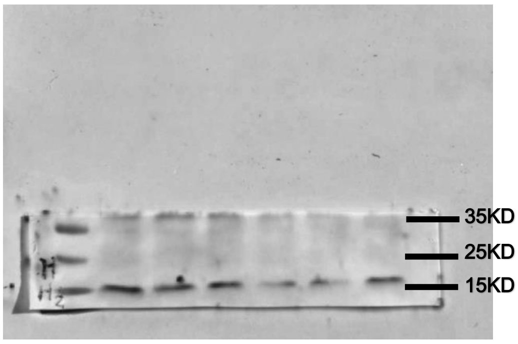

FPN1

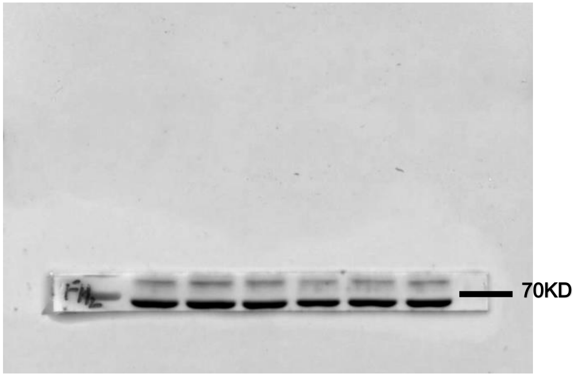

β-actin

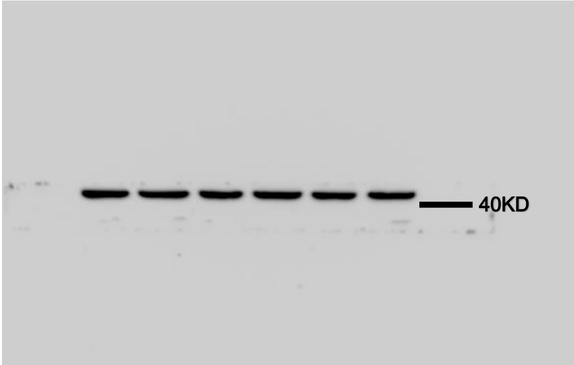

Figure 3E From left to right: *Cp*$^{fl/fl}$ *1*, *Cp*$^{fl/fl}$ *2*, *Cp*$^{fl/fl}$ 3, *Cp*^{Gfap}*cKO* 1, *Cp*^{Gfap}*cKO* 2, *Cp*^{Gfap}*cKO* 3

TfR1

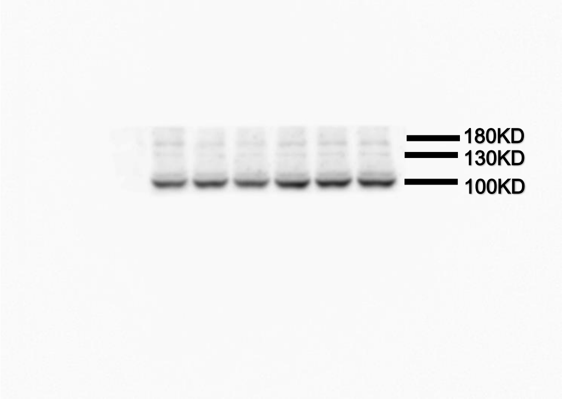

β-actin

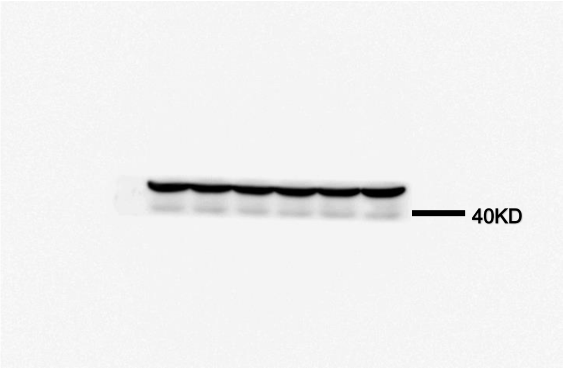

FPN1

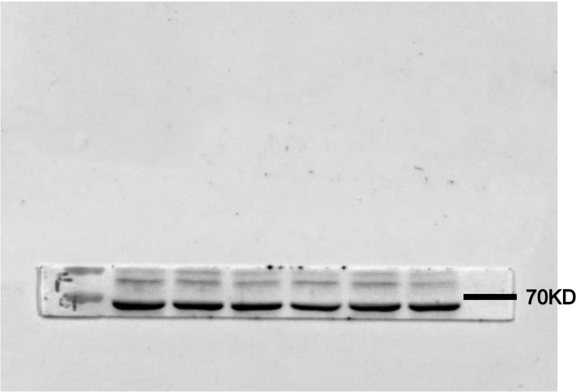

β-actin

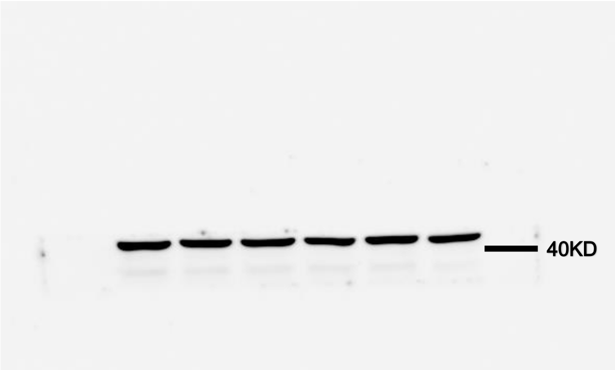

Figure 3G From left to right: *Cp*$^{fl/fl}$ *1*, *Cp*$^{fl/fl}$ *2*, *Cp*$^{fl/fl}$ 3, *Cp*^{Gfap}*cKO* 1, *Cp*^{Gfap}*cKO* 2, *Cp*^{Gfap}*cKO* 3

TfR1

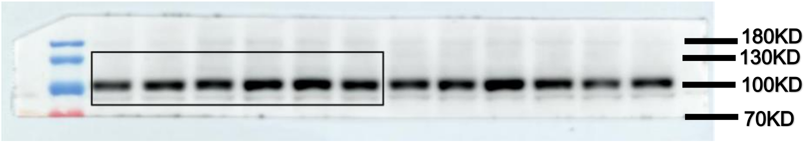

β-actin

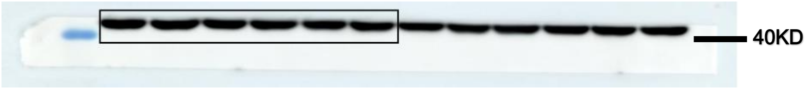

Figure 4B

H-ferritin

From left to right: From left to right: $Cp^{fl/fl}$ *1*, $Cp^{fl/fl}$ *2*, $Cp^{Gfap}cKO$ 1, $Cp^{Gfap}cKO$ 2, $Cp^{fl/fl}$ 3, $Cp^{fl/fl}$ 4, $Cp^{Gfap}cKO$ 3, $Cp^{Gfap}cKO$ 4

H-ferritin

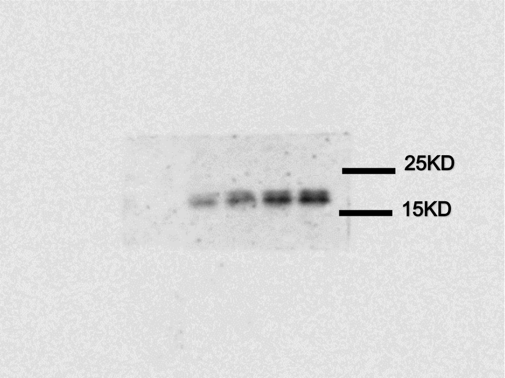

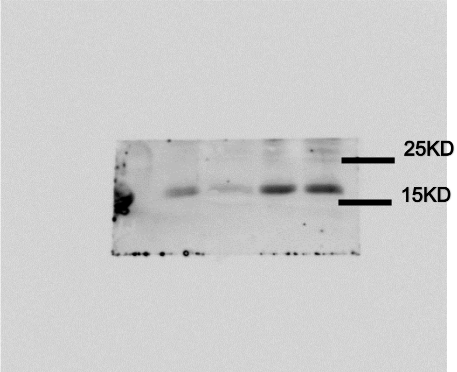

β-actin

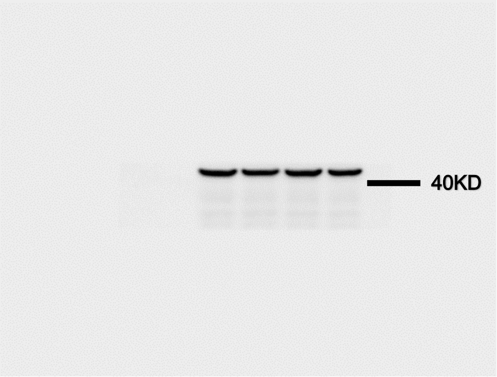

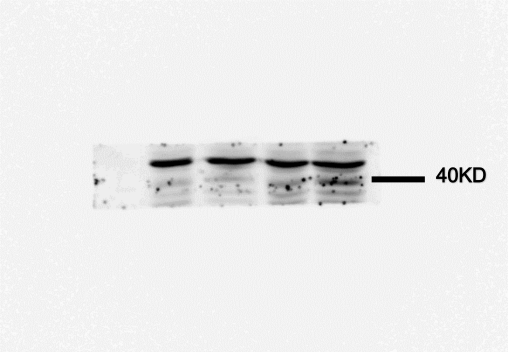

FPN1 and β-actin

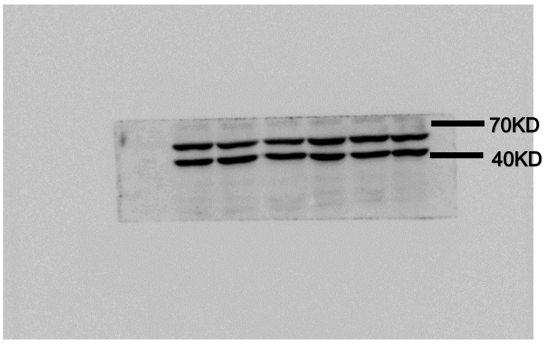

TfR1

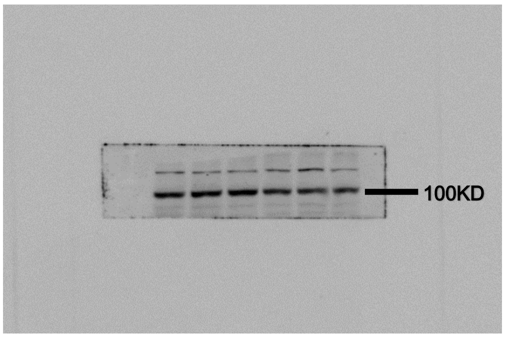

Figure 5G

From left to right: $Cp^{fl/fl}$ *1*, $Cp^{fl/fl}$ *2*, $Cp^{fl/fl}$ 3, Cp^{Gfap}*cKO* 1, Cp^{Gfap}*cKO* 2, Cp^{Gfap}*cKO* 3

Cerebral cortex

HP

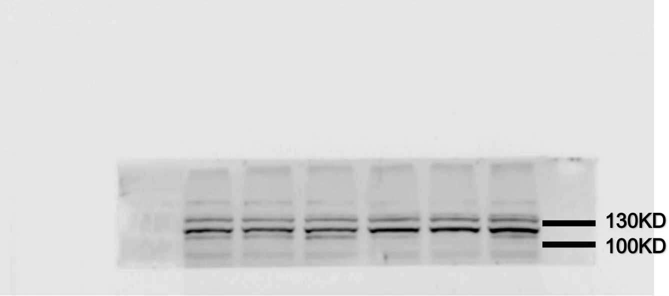

β-actin

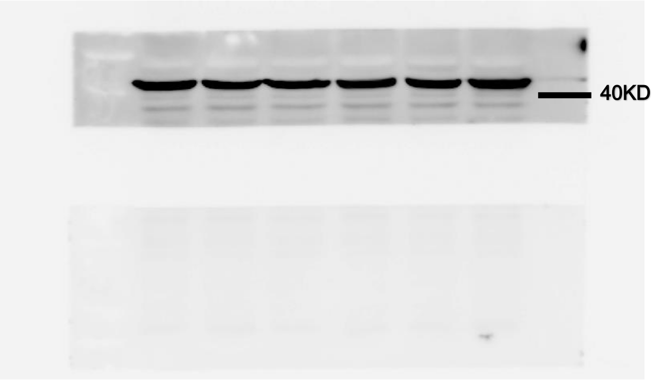

Hippocampus

HP

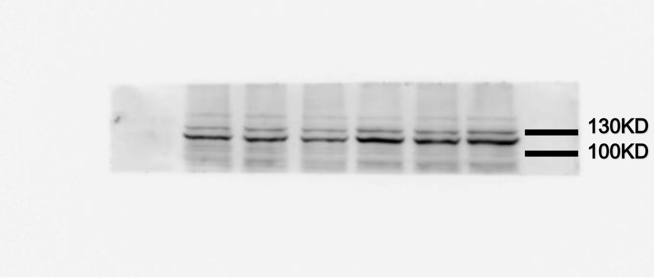

β-actin

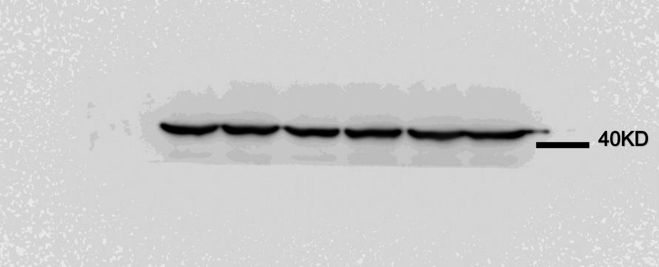

Figure 6F From left to right: 6M *Cp*$^{fl/fl}$ 1, 6M *Cp*$^{fl/fl}$ 2, 6M *Cp*$^{fl/fl}$ 3, 18M *Cp*$^{fl/fl}$ 1, 18M *Cp*$^{fl/fl}$ 2, 18M *Cp*$^{fl/fl}$ 3, 18M *Cp*^{Gfap}*cKO* 1, 18M *Cp*^{Gfap}*cKO* 2, 18M *Cp*^{Gfap}*cKO* 3

L-ferritin

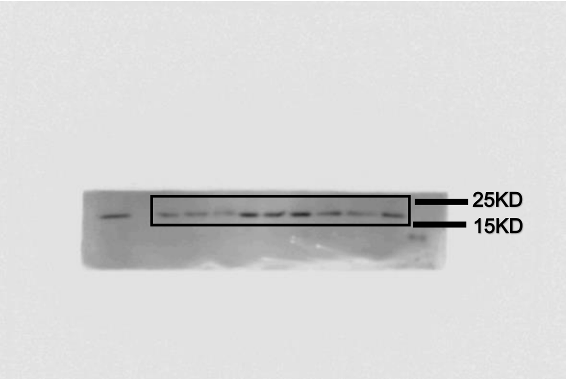

β-actin

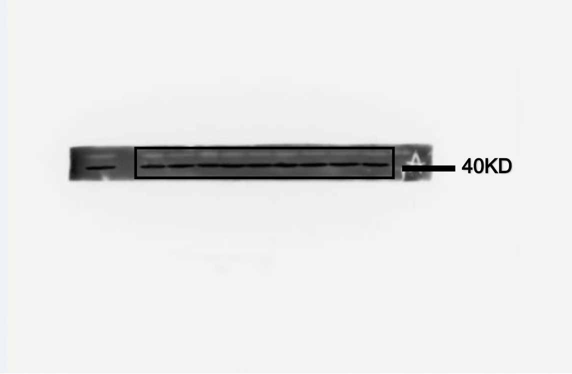

H-ferritin

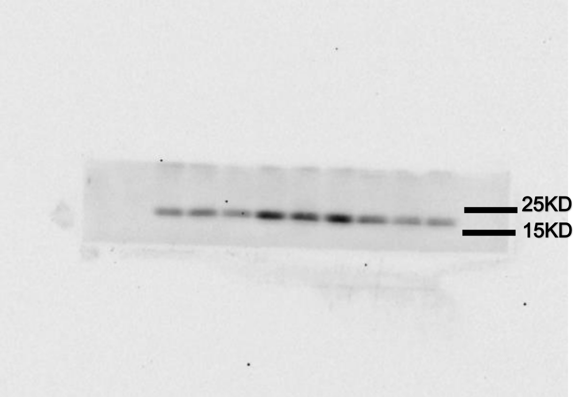

β-actin

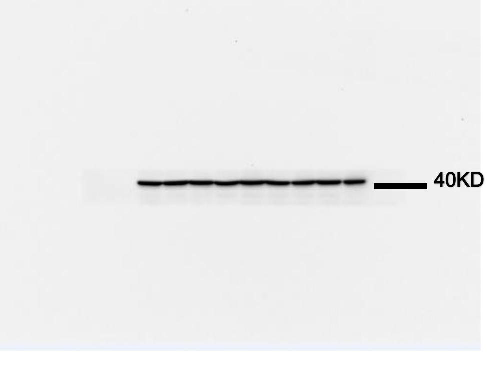

TfR1

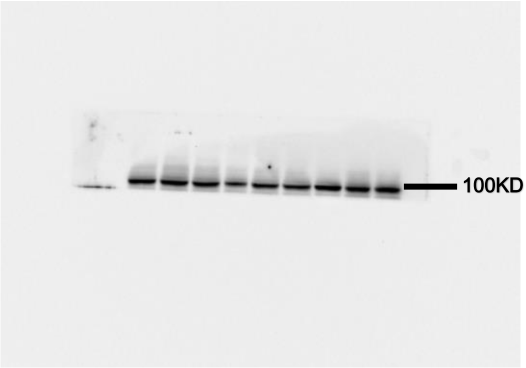

FPN1

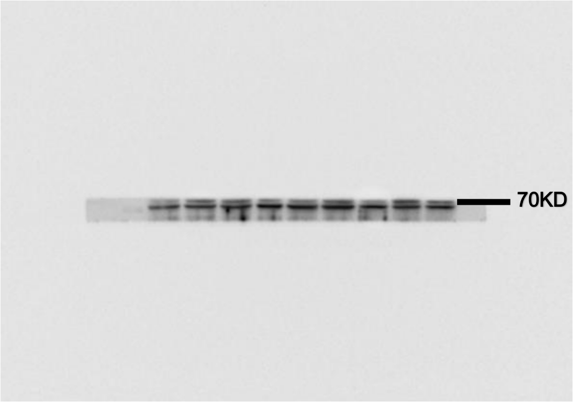

β-actin (the same as TfR1 β-actin)

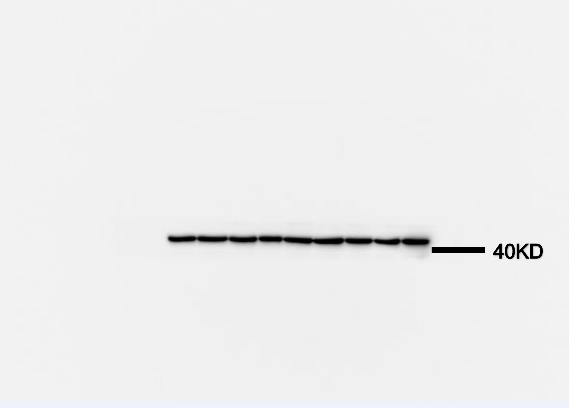

Figure 6G From left to right: 6M $Cp^{fl/fl}$ 1, 6M $Cp^{fl/fl}$ 2, 6M $Cp^{fl/fl}$ 3, 18M $Cp^{fl/fl}$ 1, 18M $Cp^{fl/fl}$ 2, 18M $Cp^{fl/fl}$ 3, 18M $Cp^{Gfap}cKO$ 1, 18M $Cp^{Gfap}cKO$ 2, 18M $Cp^{Gfap}cKO$ 3

L-ferritin

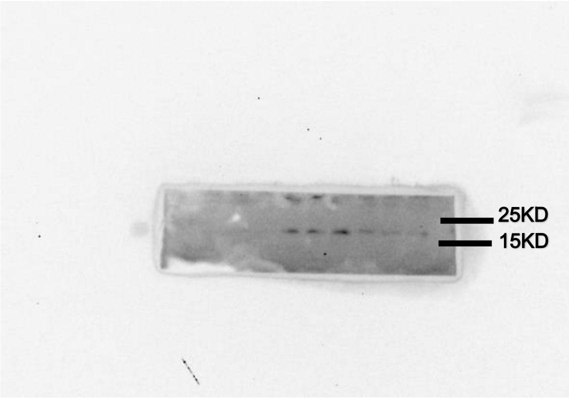

β-actin

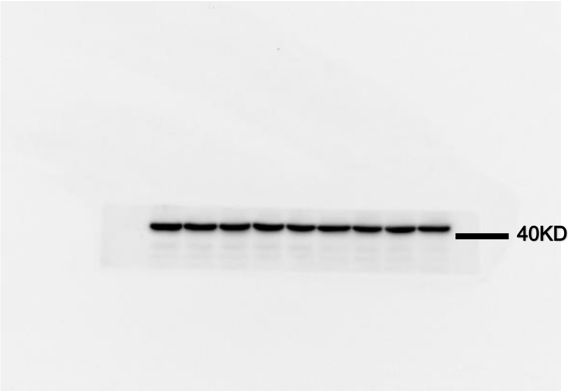

H-ferritin

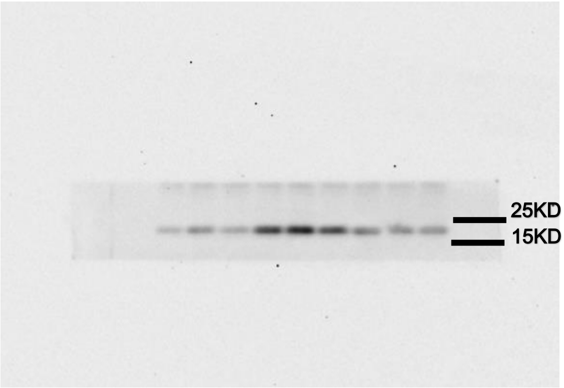

β-actin

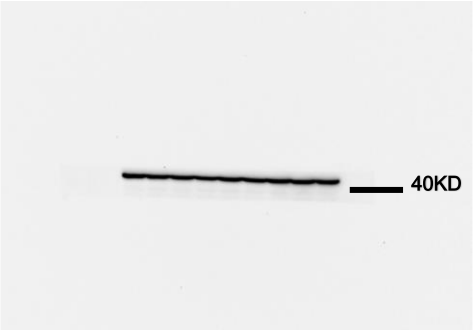

TfR1

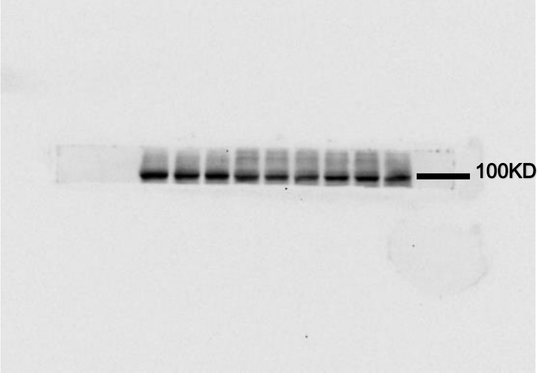

β-actin

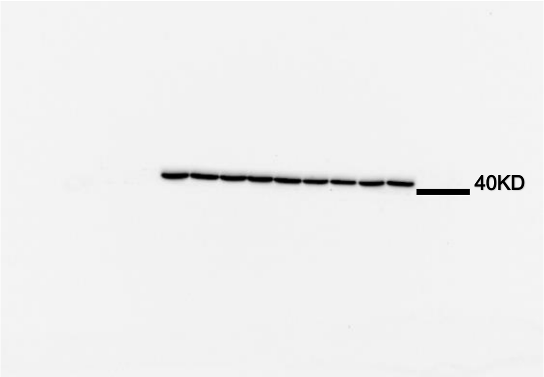

FPN1

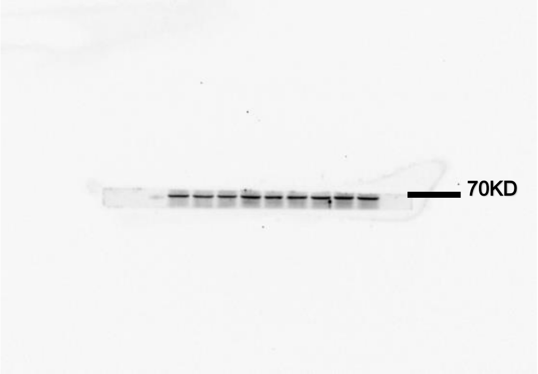

β-actin

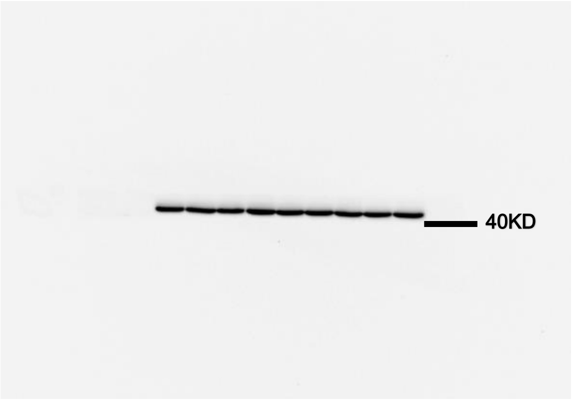

Figure 6S From left to right: 6M *Cp*$^{fl/fl}$ 1, 6M *Cp*$^{fl/fl}$ 2, 6M *Cp*$^{fl/fl}$ 3, 18M *Cp*$^{fl/fl}$ 1, 18M *Cp*$^{fl/fl}$ 2, 18M *Cp*$^{fl/fl}$ 3, 18M *Cp*^{Gfap}*cKO* 1, 18M *Cp*^{Gfap}*cKO* 2, 18M *Cp*^{Gfap}*cKO* 3

HP Cerebral cortex

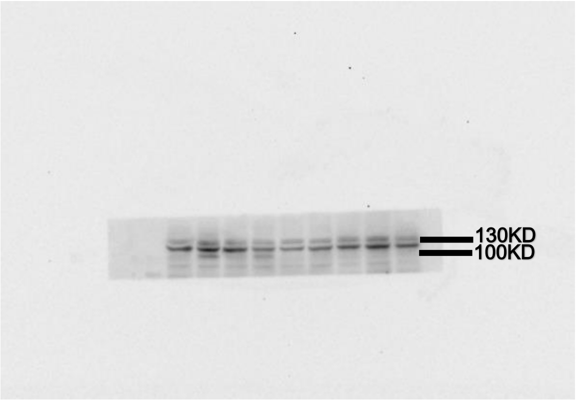

β-actin

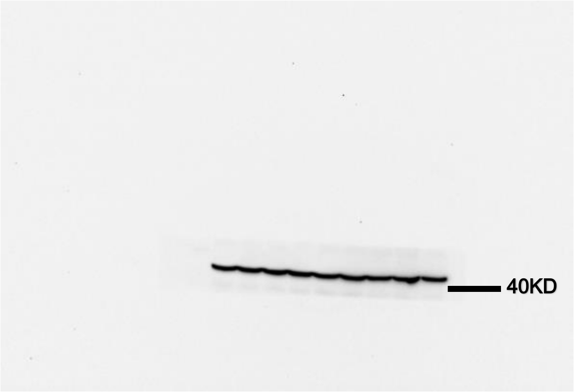

HP Hippocampus

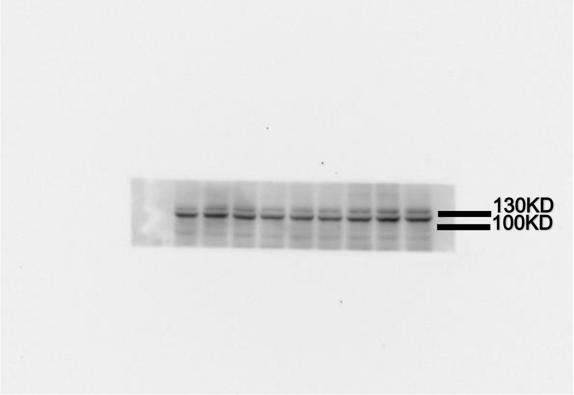

β-actin

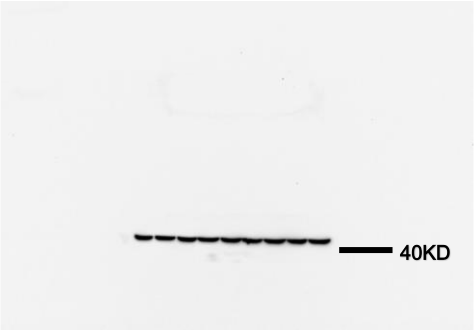

Figure 7B

Cerebral cortex

Bcl-2

From left to right: 6M *Cp*$^{fl/fl}$ 3, 18M *Cp*$^{fl/fl}$ 3, 18M *Cp*^{Gfap}*cKO* 3, 6M *Cp*$^{fl/fl}$ 1, 6M *Cp*$^{fl/fl}$ 2, 18M *Cp*$^{fl/fl}$ 1, 18M *Cp*$^{fl/fl}$ 2, 18M *Cp*^{Gfap}*cKO* 1, 18M *Cp*^{Gfap}*cKO* 2

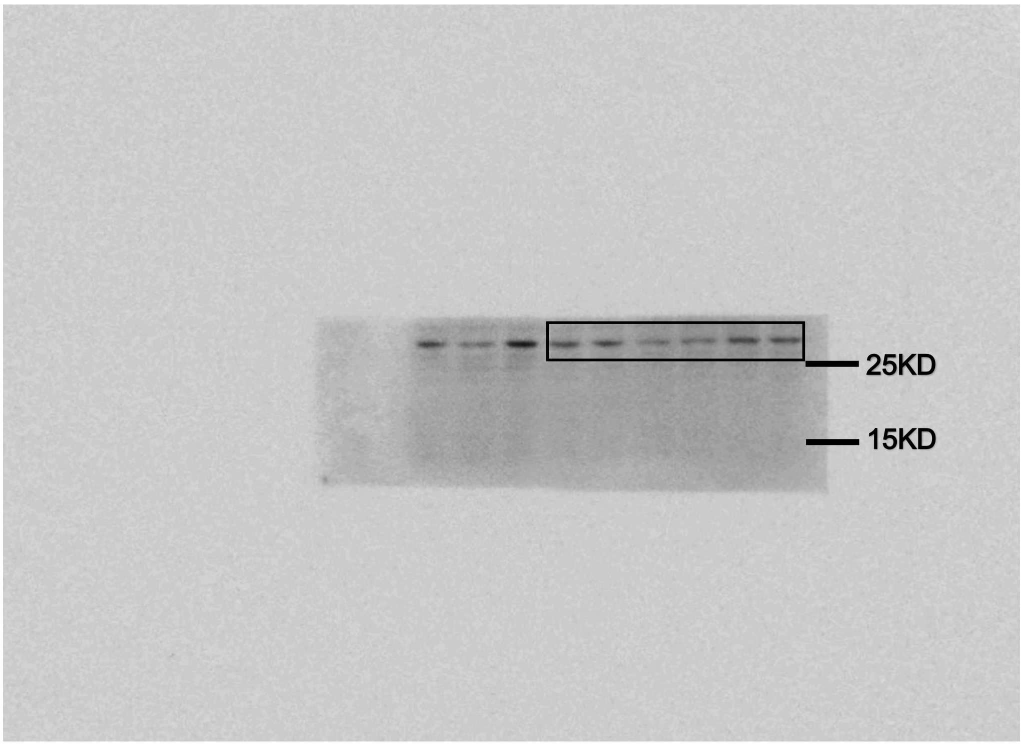

Bax

From left to right: 6M *Cp*$^{fl/fl}$ 3, 18M *Cp*$^{fl/fl}$ 3, 18M *Cp*^{Gfap}*cKO* 3, 6M *Cp*$^{fl/fl}$ 1, 6M *Cp*$^{fl/fl}$ 2, 18M *Cp*$^{fl/fl}$ 1, 18M *Cp*$^{fl/fl}$ 2, 18M *Cp*^{Gfap}*cKO* 1, 18M *Cp*^{Gfap}*cKO* 2

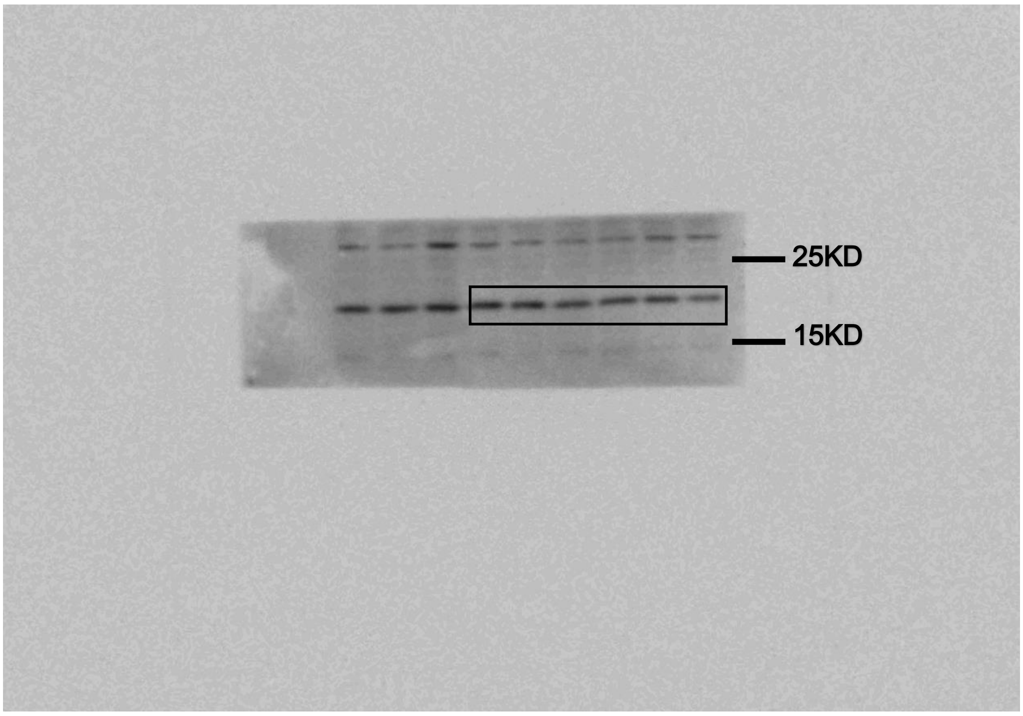

β-actin

From left to right: 6M *Cp*$^{fl/fl}$ 3, 18M *Cp*$^{fl/fl}$ 3, 18M *Cp*^{Gfap}*cKO* 3, 6M *Cp*$^{fl/fl}$ 1, 6M *Cp*$^{fl/fl}$ 2, 18M *Cp*$^{fl/fl}$ 1, 18M *Cp*$^{fl/fl}$ 2, 18M *Cp*^{Gfap}*cKO* 1, 18M *Cp*^{Gfap}*cKO* 2

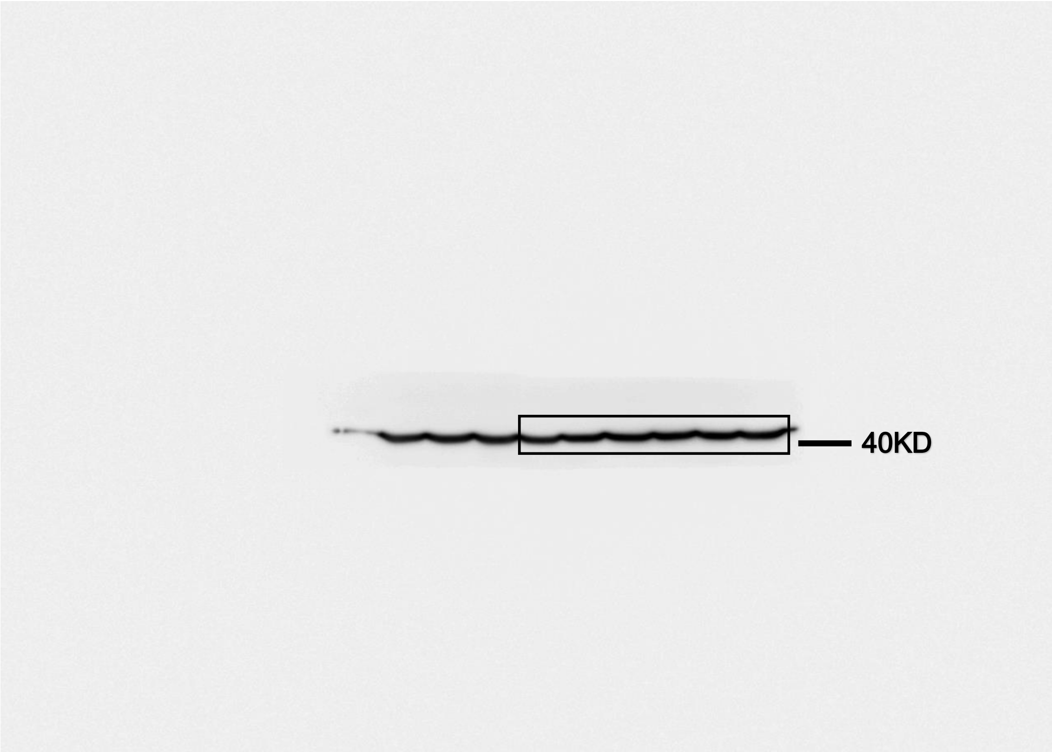

p-Erk

From left to right: 6M *Cp*$^{fl/fl}$ 1, 6M *Cp*$^{fl/fl}$ 2, 18M *Cp*$^{fl/fl}$ 1, 18M *Cp*$^{fl/fl}$ 2, 18M *Cp*^{Gfap}*cKO* 1, 18M *Cp*^{Gfap}*cKO* 2, 6M *Cp*$^{fl/fl}$ 3, 18M *Cp*$^{fl/fl}$ 3, 18M *Cp*^{Gfap}*cKO* 3, 6M *Cp*$^{fl/fl}$ 4, 18M *Cp*$^{fl/fl}$ 4, 18M *Cp*^{Gfap}*cKO* 4

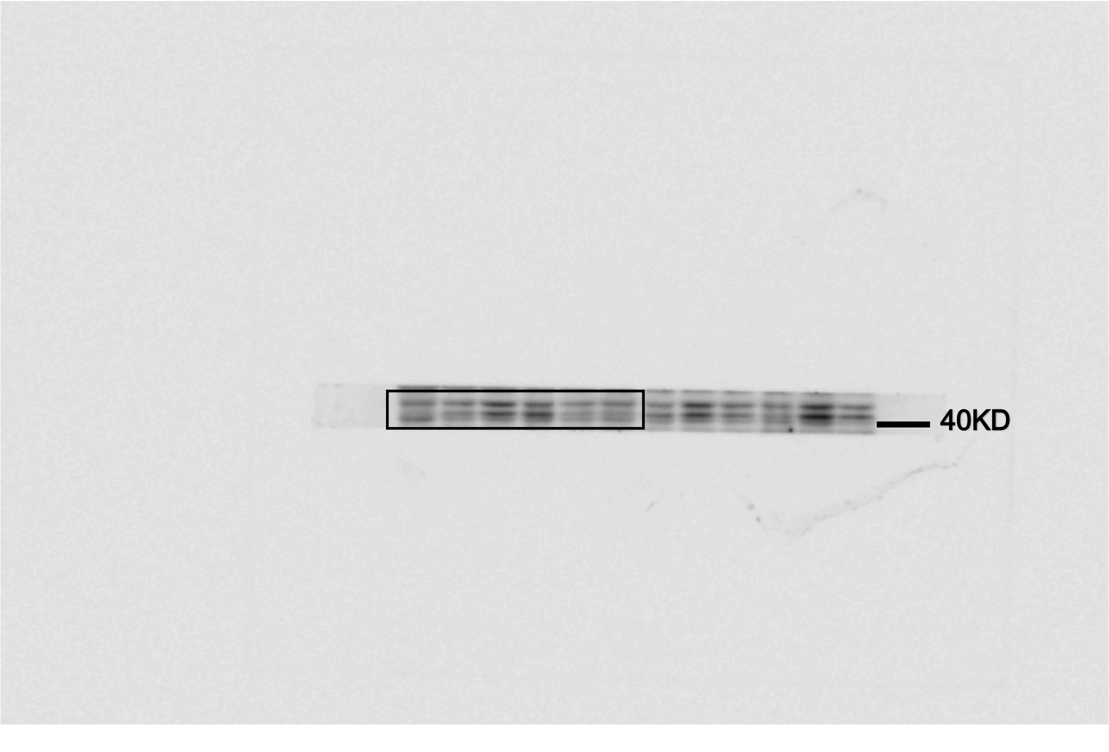

Erk

From left to right: 6M $Cp^{fl/fl}$ 1, 6M $Cp^{fl/fl}$ 2, 18M $Cp^{fl/fl}$ 1, 18M $Cp^{fl/fl}$ 2, 18M $Cp^{Gfap}cKO$ 1, 18M $Cp^{Gfap}cKO$ 2, 6M $Cp^{fl/fl}$ 3, 18M $Cp^{fl/fl}$ 3, 18M $Cp^{Gfap}cKO$ 3, 6M $Cp^{fl/fl}$ 4, 18M $Cp^{fl/fl}$ 4, 18M $Cp^{Gfap}cKO$ 4

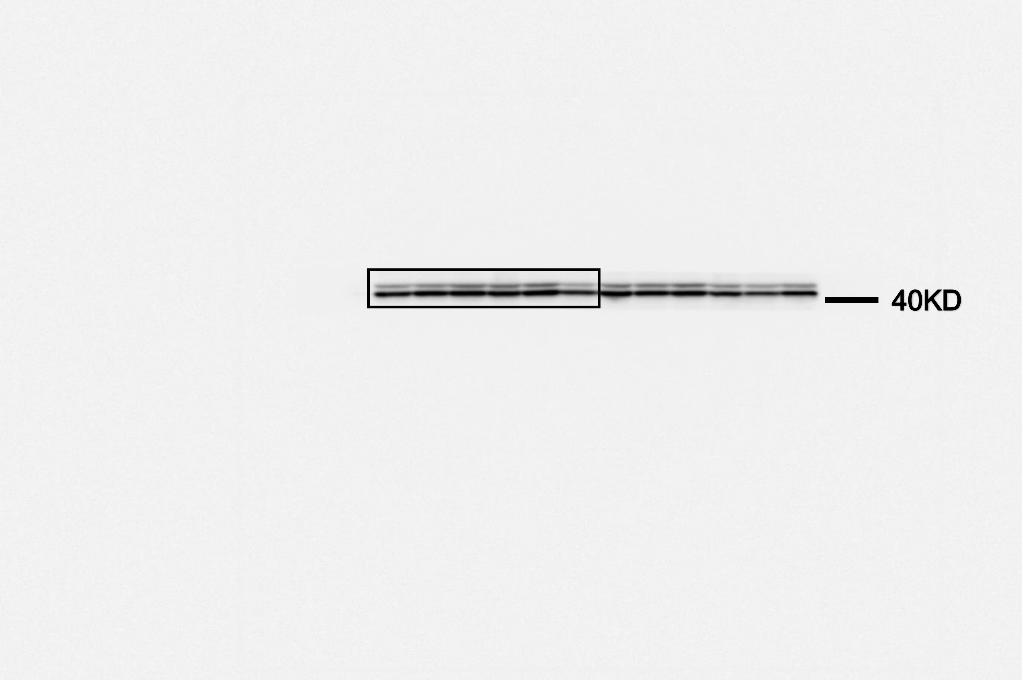

p-p38

From left to right: 6M $Cp^{fl/fl}$ 1, 6M $Cp^{fl/fl}$ 2, 18M $Cp^{fl/fl}$ 1, 18M $Cp^{fl/fl}$ 2, 18M $Cp^{Gfap}cKO$ 1, 18M $Cp^{Gfap}cKO$ 2, 6M $Cp^{fl/fl}$ 3, 18M $Cp^{fl/fl}$ 3, 18M $Cp^{Gfap}cKO$ 3, 6M $Cp^{fl/fl}$ 4, 18M $Cp^{fl/fl}$ 4, 18M $Cp^{Gfap}cKO$ 4

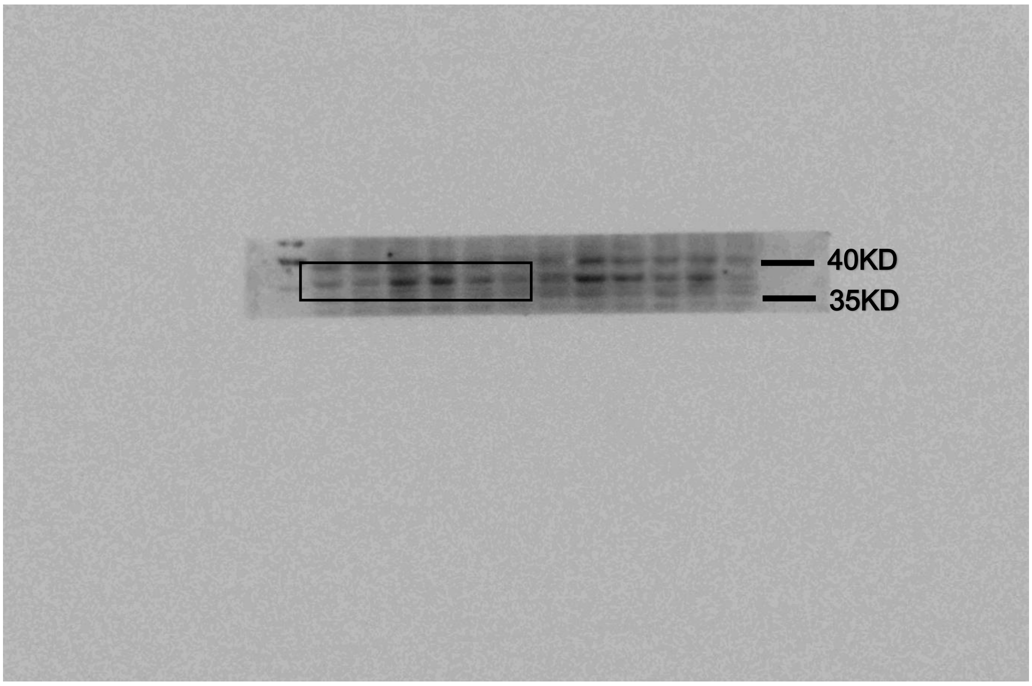

p38

From left to right: 6M $Cp^{fl/fl}$ 1, 6M $Cp^{fl/fl}$ 2, 18M $Cp^{fl/fl}$ 1, 18M $Cp^{fl/fl}$ 2, 18M $Cp^{Gfap}cKO$ 1, 18M $Cp^{Gfap}cKO$ 2, 6M $Cp^{fl/fl}$ 3, 18M $Cp^{fl/fl}$ 3, 18M $Cp^{Gfap}cKO$ 3, 6M $Cp^{fl/fl}$ 4, 18M $Cp^{fl/fl}$ 4, 18M $Cp^{Gfap}cKO$ 4

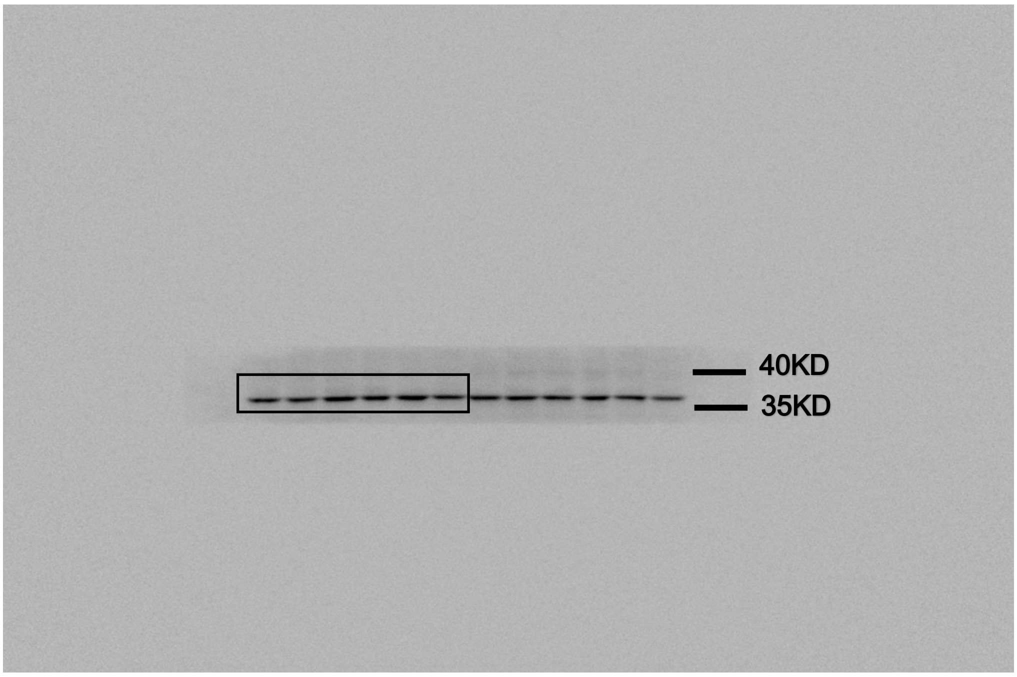

Hippocampus

Bcl-2

From left to right: 6M $Cp^{fl/fl}$ 1, 6M $Cp^{fl/fl}$ 2, 18M $Cp^{fl/fl}$ 1, 18M $Cp^{fl/fl}$ 2, 18M $Cp^{Gfap}cKO$ 1, 18M $Cp^{Gfap}cKO$ 2, 6M $Cp^{fl/fl}$ 3, 18M $Cp^{fl/fl}$ 3, 18M $Cp^{Gfap}cKO$ 3, 6M $Cp^{fl/fl}$ 4, 18M $Cp^{fl/fl}$ 4, 18M $Cp^{Gfap}cKO$ 4

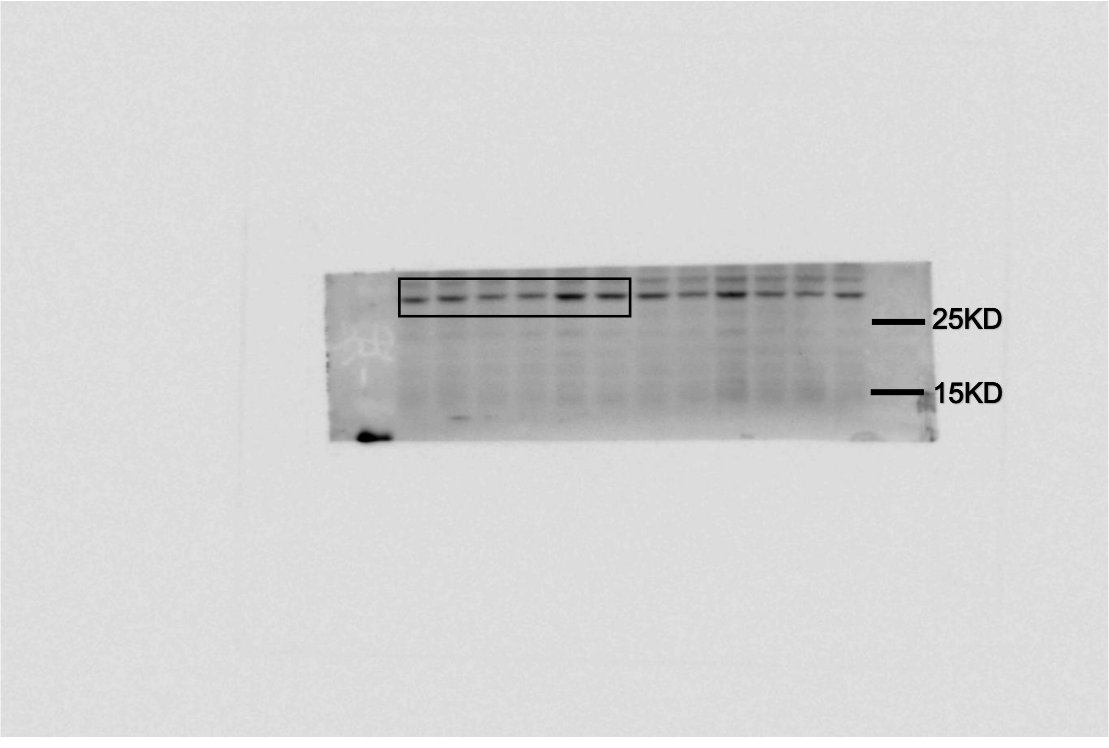

Bax

From left to right: 6M $Cp^{fl/fl}$ 1, 6M $Cp^{fl/fl}$ 2, 18M $Cp^{fl/fl}$ 1, 18M $Cp^{fl/fl}$ 2, 18M $Cp^{Gfap}cKO$ 1, 18M $Cp^{Gfap}cKO$ 2, 6M $Cp^{fl/fl}$ 3, 18M $Cp^{fl/fl}$ 3, 18M $Cp^{Gfap}cKO$ 3, 6M $Cp^{fl/fl}$ 4, 18M $Cp^{fl/fl}$ 4, 18M $Cp^{Gfap}cKO$ 4

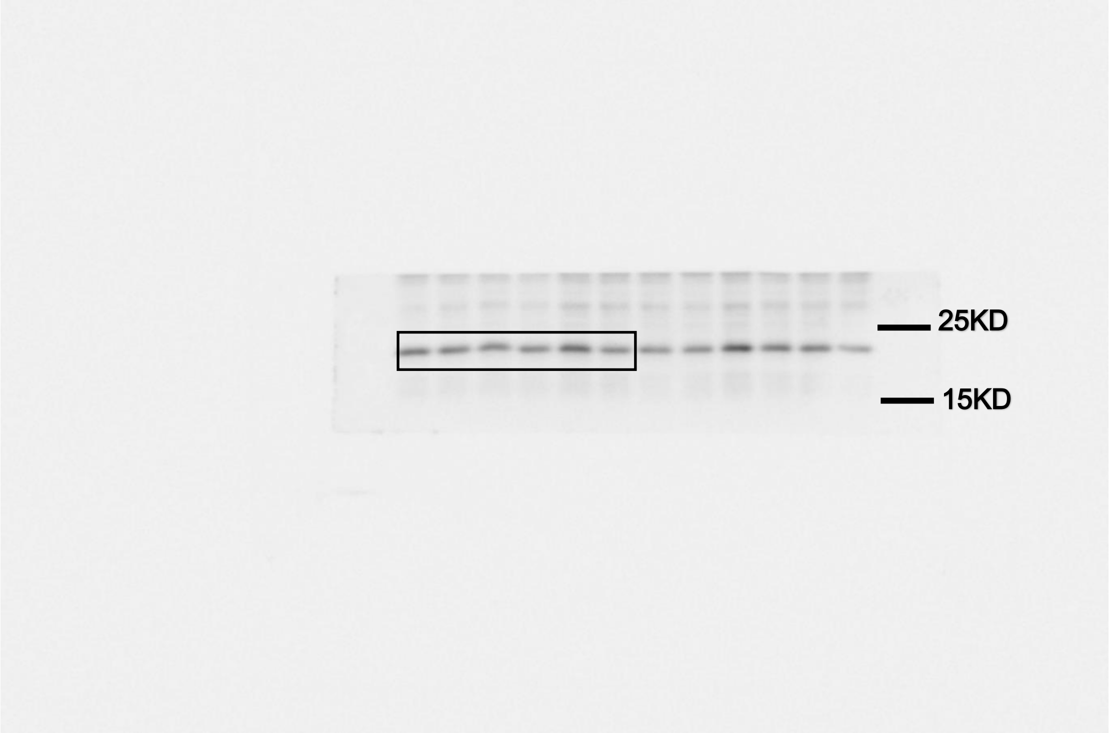

β-actin

From left to right: 6M $Cp^{fl/fl}$ 1, 6M $Cp^{fl/fl}$ 2, 18M $Cp^{fl/fl}$ 1, 18M $Cp^{fl/fl}$ 2, 18M $Cp^{Gfap}cKO$ 1, 18M $Cp^{Gfap}cKO$ 2, 6M $Cp^{fl/fl}$ 3, 18M $Cp^{fl/fl}$ 3, 18M $Cp^{Gfap}cKO$ 3, 6M $Cp^{fl/fl}$ 4, 18M $Cp^{fl/fl}$ 4, 18M $Cp^{Gfap}cKO$ 4

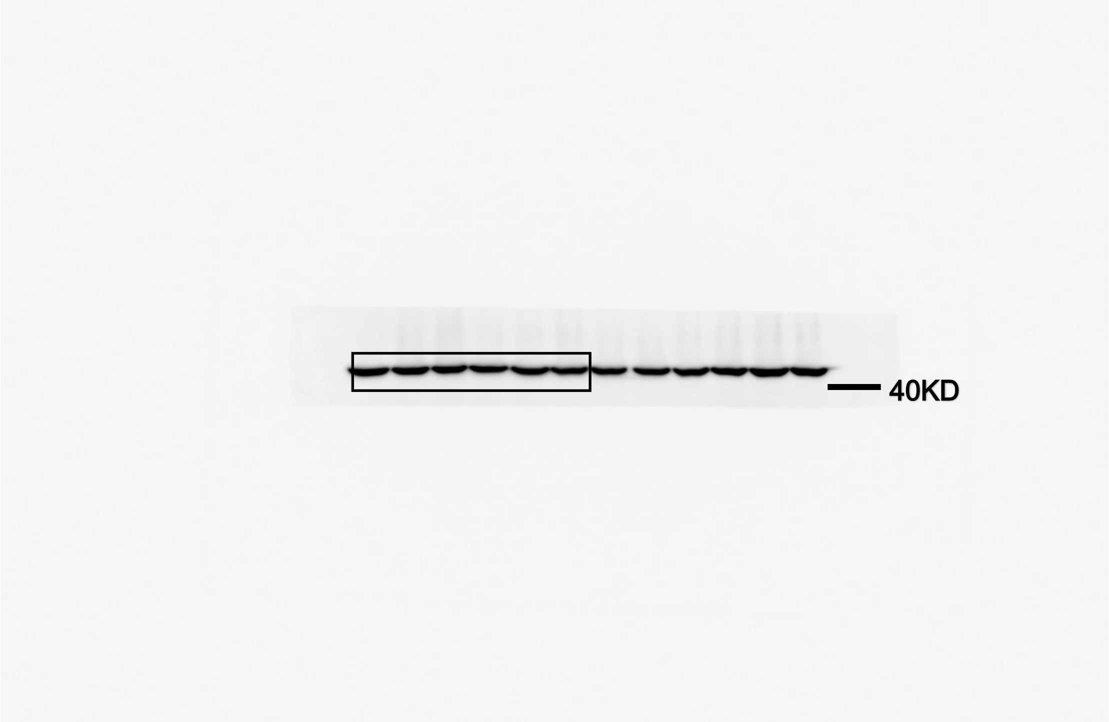

p-Erk

From left to right: 6M *Cp*$^{fl/fl}$ 1, 6M *Cp*$^{fl/fl}$ 2, 18M *Cp*$^{fl/fl}$ 1, 18M *Cp*$^{fl/fl}$ 2, 18M *Cp*^{Gfap}*cKO* 1, 18M *Cp*^{Gfap}*cKO* 2, 6M *Cp*$^{fl/fl}$ 3, 18M *Cp*$^{fl/fl}$ 3, 18M *Cp*^{Gfap}*cKO* 3, 6M *Cp*$^{fl/fl}$ 4, 18M *Cp*$^{fl/fl}$ 4, 18M *Cp*^{Gfap}*cKO* 4

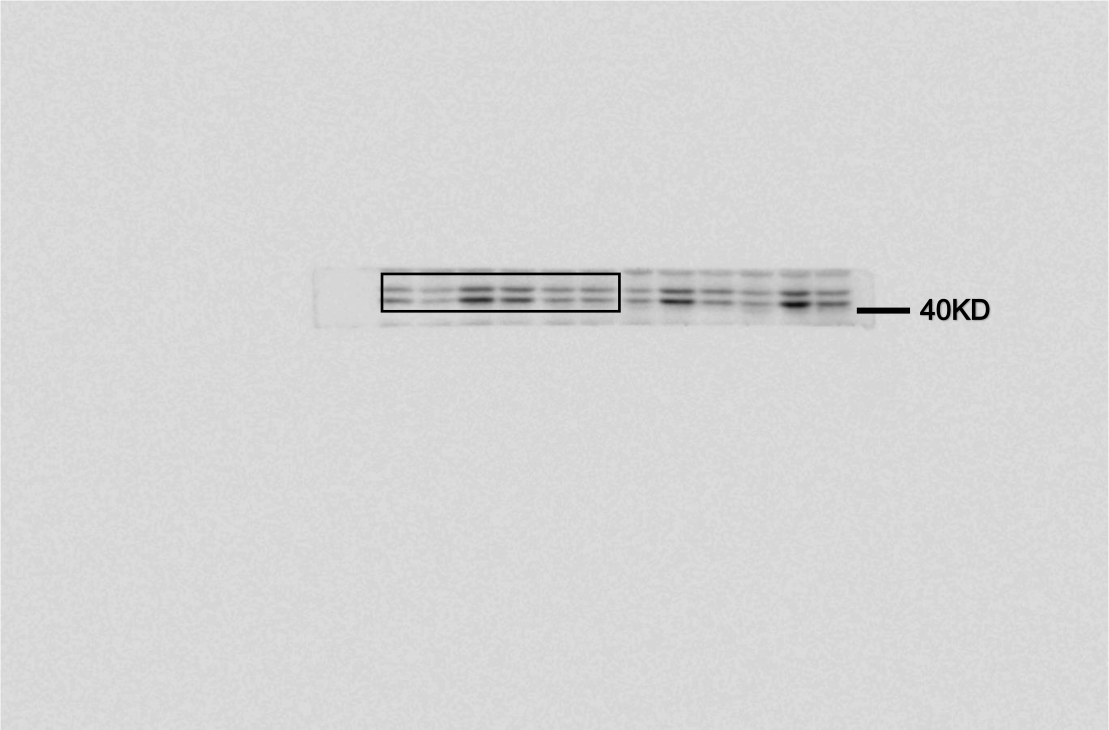

Erk

From left to right: 6M *Cp*$^{fl/fl}$ 1, 6M *Cp*$^{fl/fl}$ 2, 18M *Cp*$^{fl/fl}$ 1, 18M *Cp*$^{fl/fl}$ 2, 18M *Cp*^{Gfap}*cKO* 1, 18M *Cp*^{Gfap}*cKO* 2, 6M *Cp*$^{fl/fl}$ 3, 18M *Cp*$^{fl/fl}$ 3, 18M *Cp*^{Gfap}*cKO* 3, 6M *Cp*$^{fl/fl}$ 4, 18M *Cp*$^{fl/fl}$ 4, 18M *Cp*^{Gfap}*cKO* 4

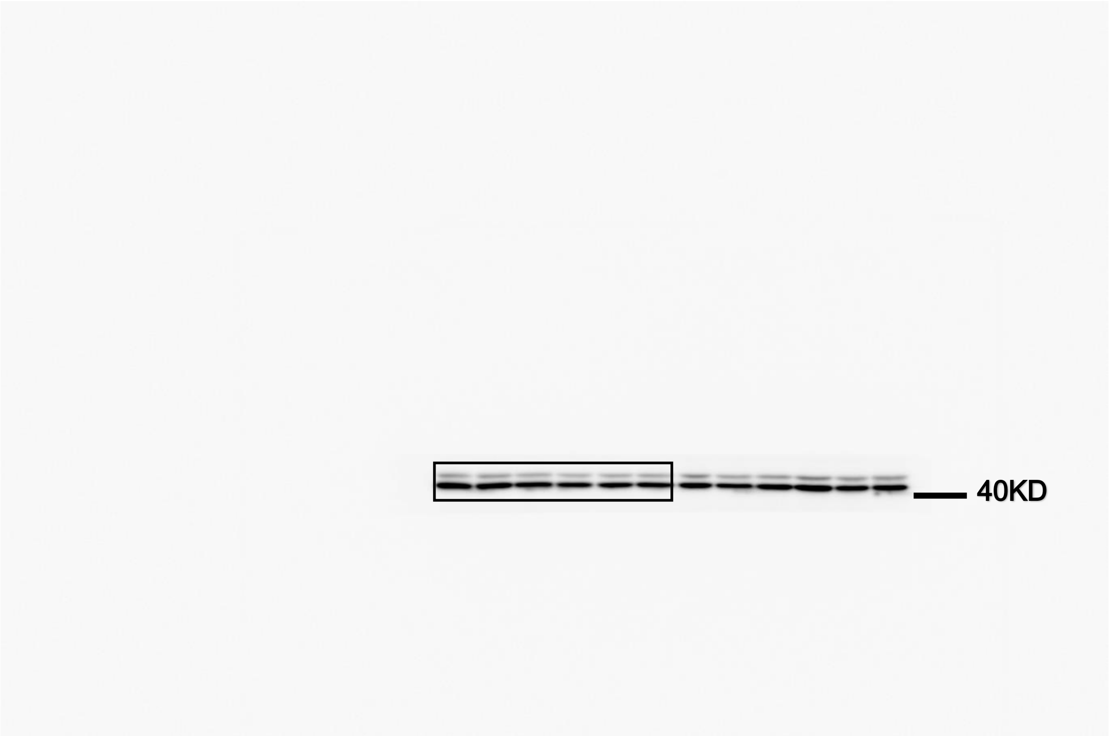

p-p38

From left to right: 6M *Cp*$^{fl/fl}$ 1, 6M *Cp*$^{fl/fl}$ 2, 18M *Cp*$^{fl/fl}$ 1, 18M *Cp*$^{fl/fl}$ 2, 18M *Cp*^{Gfap}*cKO* 1, 18M *Cp*^{Gfap}*cKO* 2, 6M *Cp*$^{fl/fl}$ 3, 18M *Cp*$^{fl/fl}$ 3, 18M *Cp*^{Gfap}*cKO* 3, 6M *Cp*$^{fl/fl}$ 4, 18M *Cp*$^{fl/fl}$ 4, 18M *Cp*^{Gfap}*cKO* 4

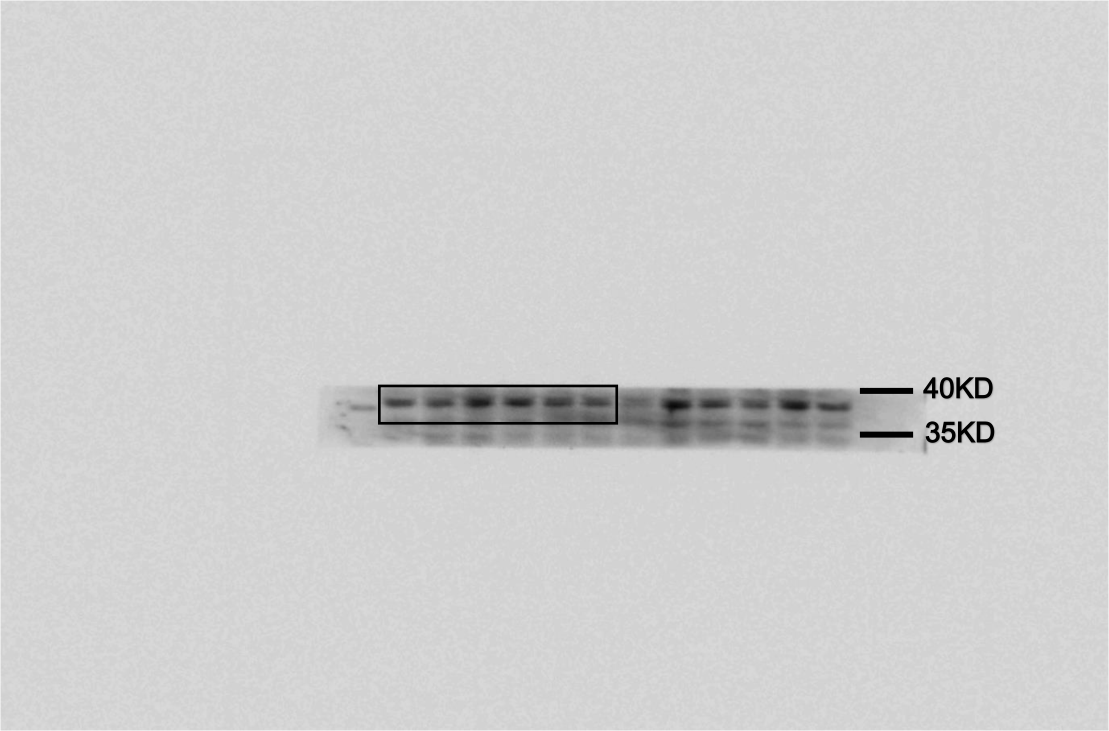

p38

From left to right: 6M *Cp*$^{fl/fl}$ 1, 6M *Cp*$^{fl/fl}$ 2, 18M *Cp*$^{fl/fl}$ 1, 18M *Cp*$^{fl/fl}$ 2, 18M *Cp*^{Gfap}*cKO* 1, 18M *Cp*^{Gfap}*cKO* 2, 6M *Cp*$^{fl/fl}$ 3, 18M *Cp*$^{fl/fl}$ 3, 18M *Cp*^{Gfap}*cKO* 3, 6M *Cp*$^{fl/fl}$ 4, 18M *Cp*$^{fl/fl}$ 4, 18M *Cp*^{Gfap}*cKO* 4

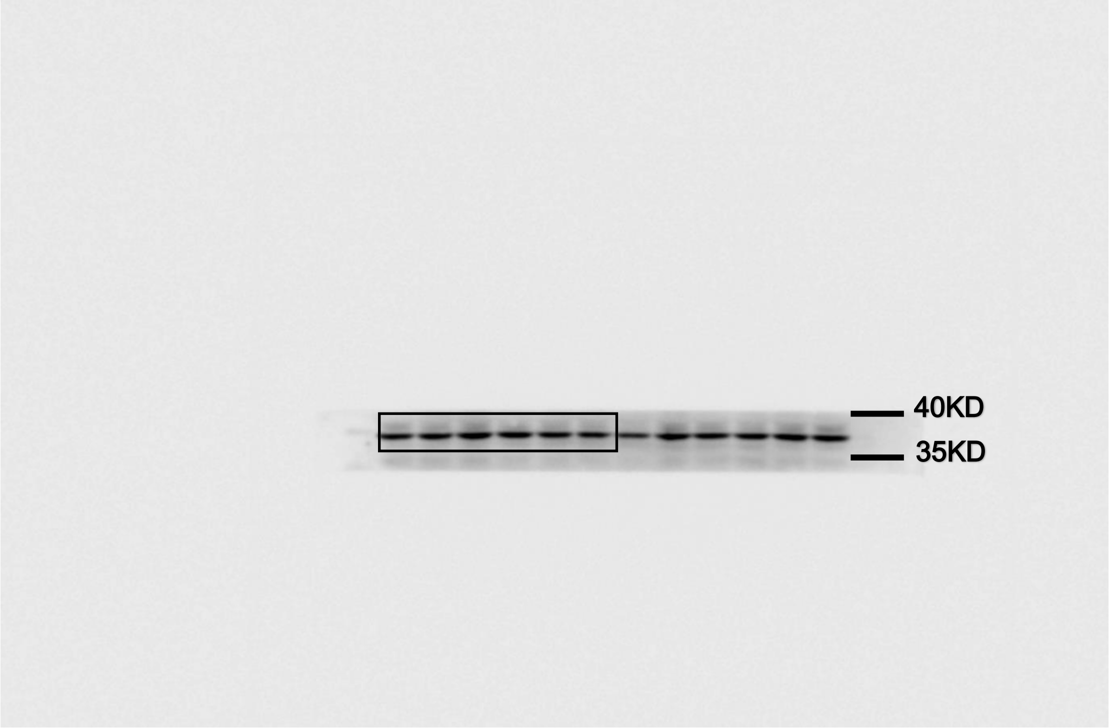

Figure 7G

ACSL4

From left to right: 6M *Cp*$^{fl/fl}$ 1, 6M *Cp*$^{fl/fl}$ 2, 6M *Cp*$^{fl/fl}$ 3, 18M *Cp*$^{fl/fl}$ 1, 18M *Cp*$^{fl/fl}$ 2, 18M *Cp*$^{fl/fl}$ 3, 18M *Cp*^{Gfap}*cKO* 1, 18M *Cp*^{Gfap}*cKO* 2, 18M *Cp*^{Gfap}*cKO* 3

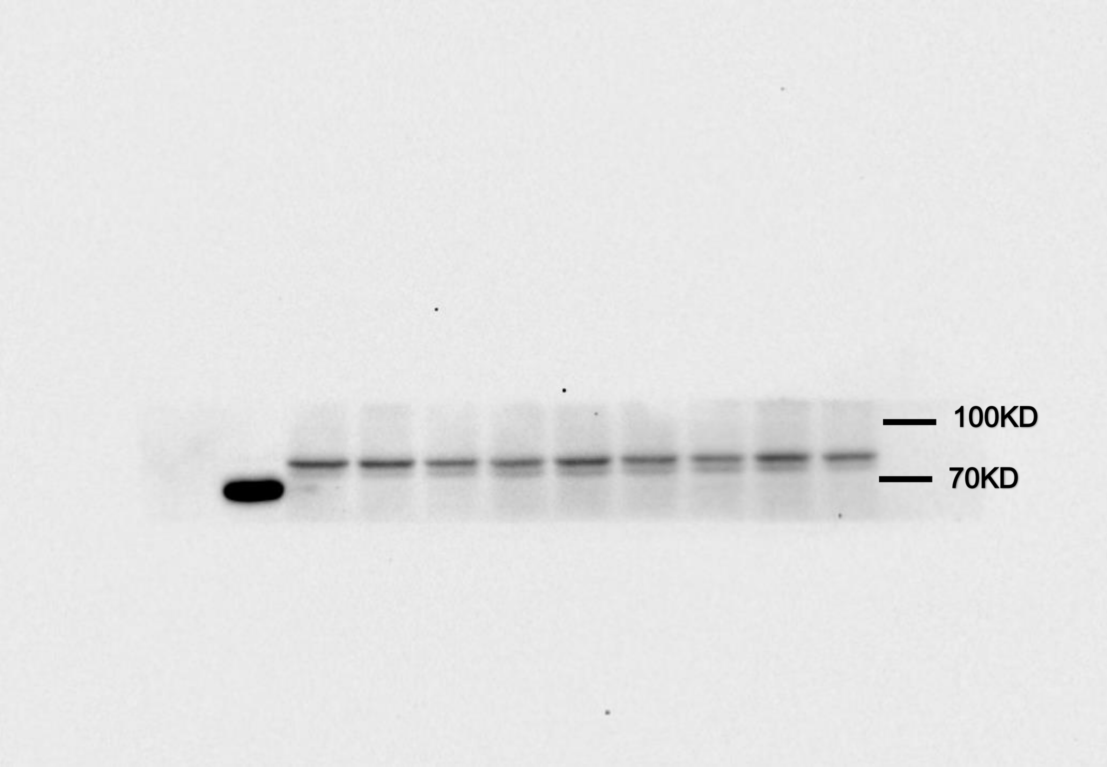

β-actin

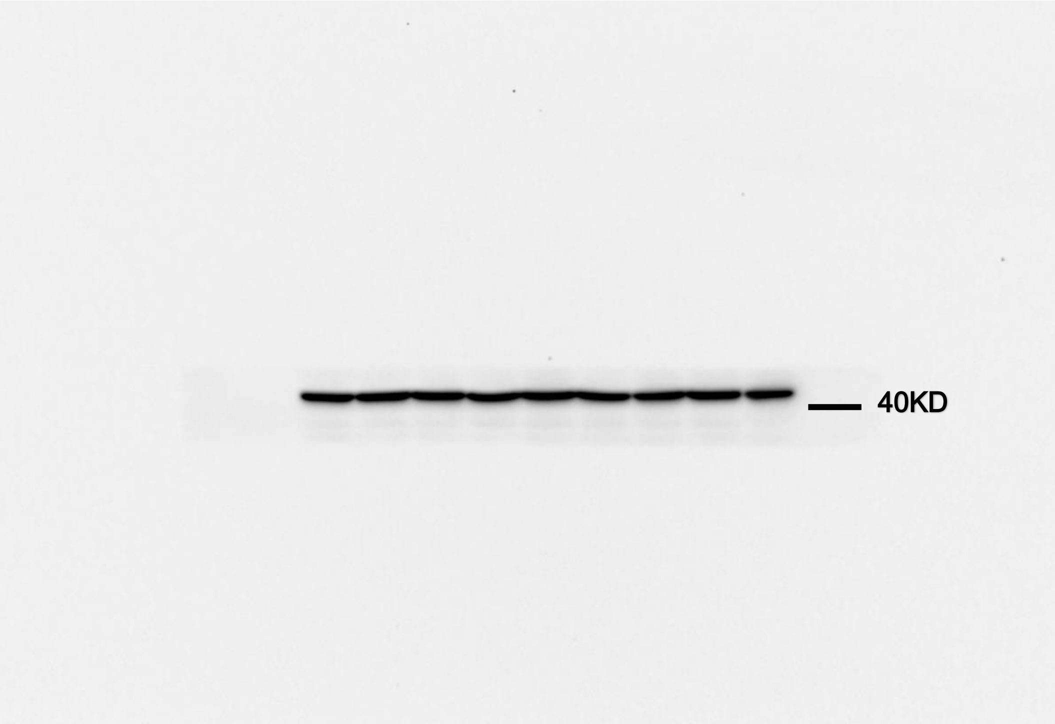

GPX4

From left to right: 6M $Cp^{fl/fl}$ 1, 6M $Cp^{fl/fl}$ 2, 6M $Cp^{fl/fl}$ 3, 18M $Cp^{fl/fl}$ 1, 18M $Cp^{fl/fl}$ 2, 18M $Cp^{fl/fl}$ 3, 18M $Cp^{Gfap}cKO$ 1, 18M $Cp^{Gfap}cKO$ 2, 18M $Cp^{Gfap}cKO$ 3, 6M $Cp^{fl/fl}$ 4, 18M $Cp^{fl/fl}$ 4, 18M $Cp^{Gfap}cKO$ 4

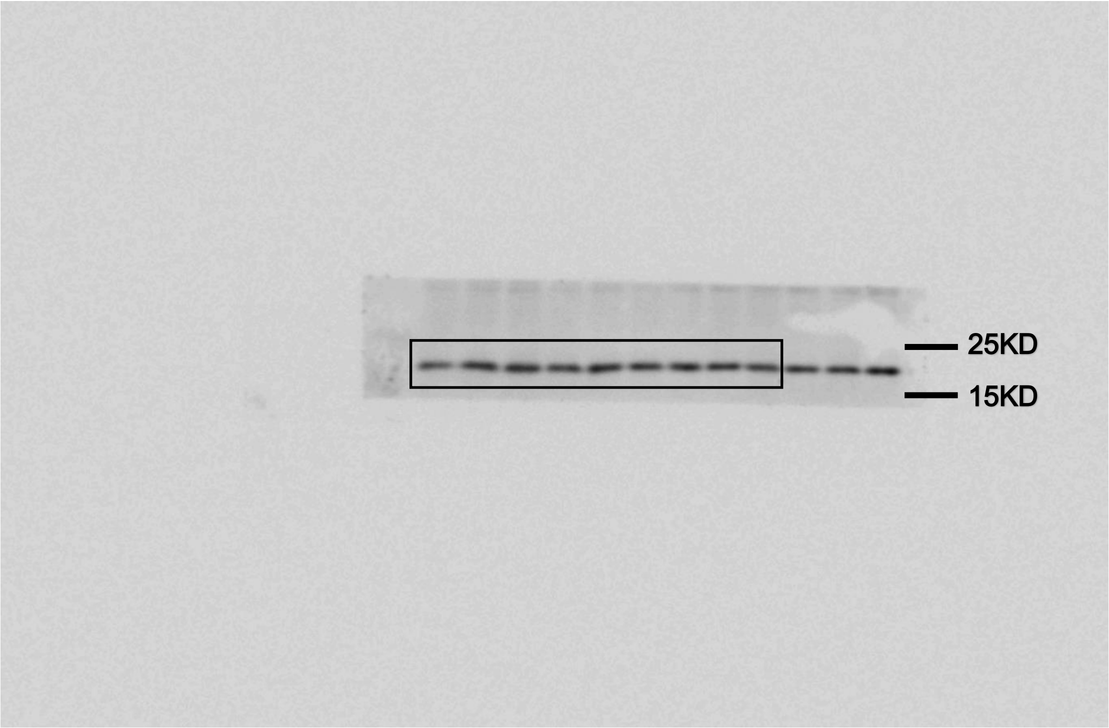

β-actin

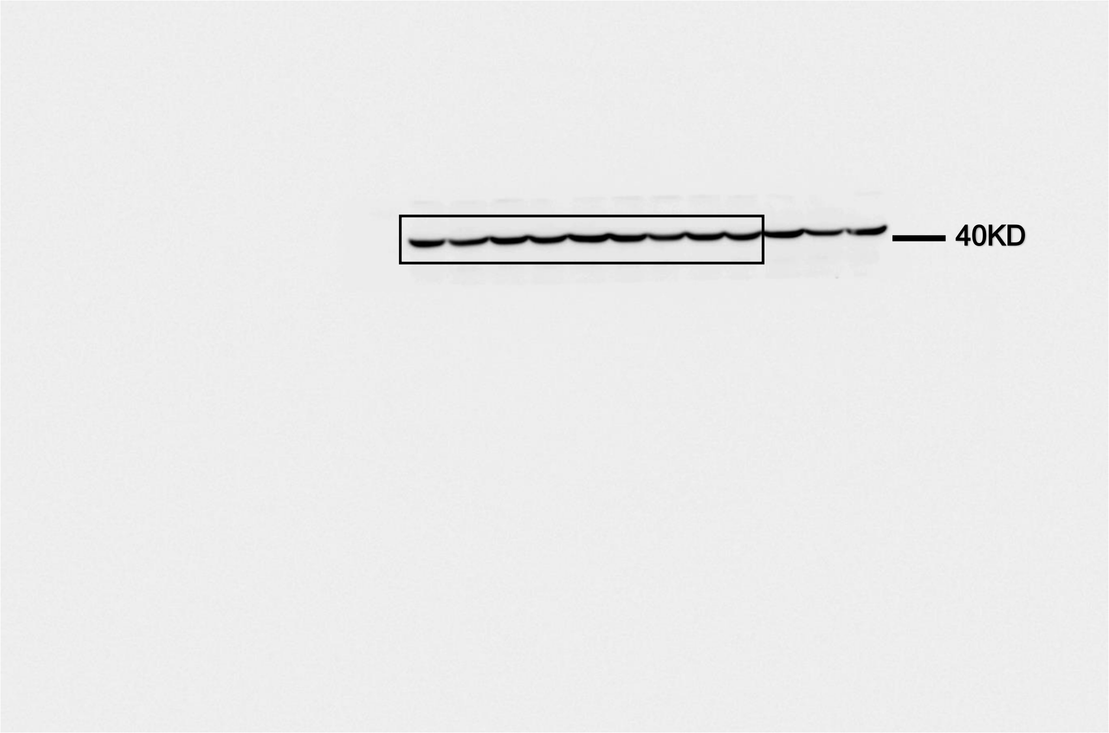

Figure 7I

ACSL4

From left to right: 6M *Cp*$^{fl/fl}$ 1, 6M *Cp*$^{fl/fl}$ 2, 6M *Cp*$^{fl/fl}$ 3, 18M *Cp*$^{fl/fl}$ 1, 18M *Cp*$^{fl/fl}$ 2, 18M *Cp*$^{fl/fl}$ 3, 18M *Cp*^{Gfap}*cKO* 1, 18M *Cp*^{Gfap}*cKO* 2, 18M *Cp*^{Gfap}*cKO* 3

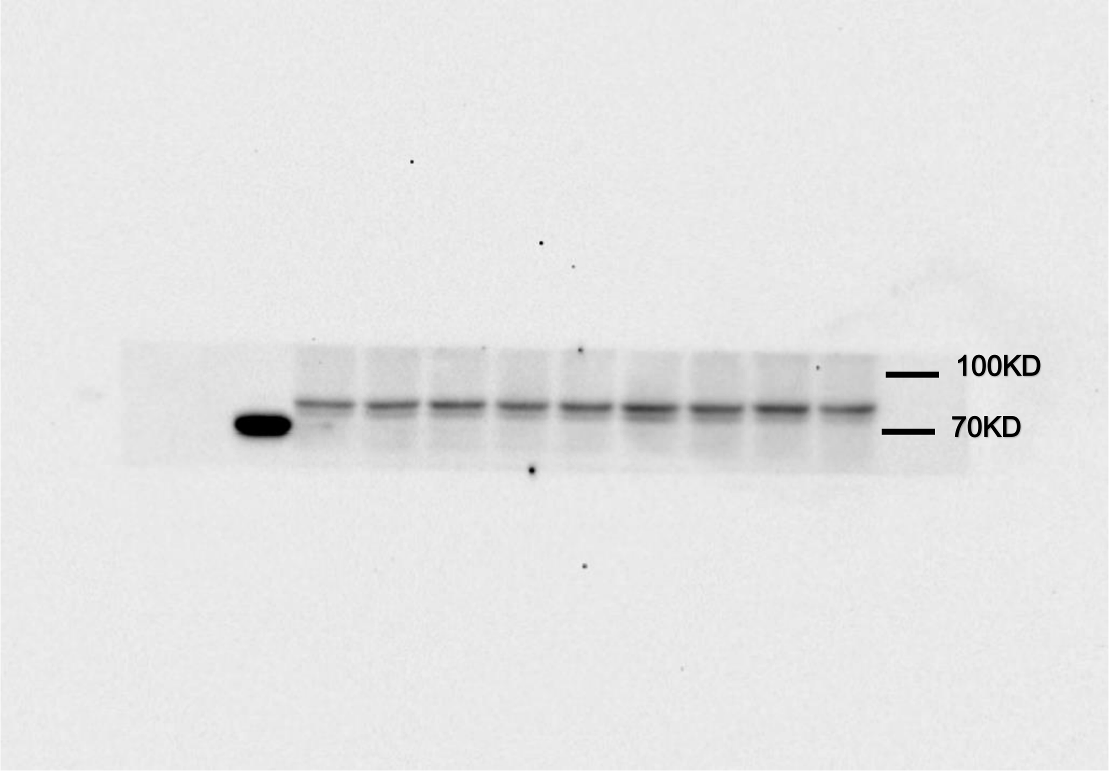

β-actin

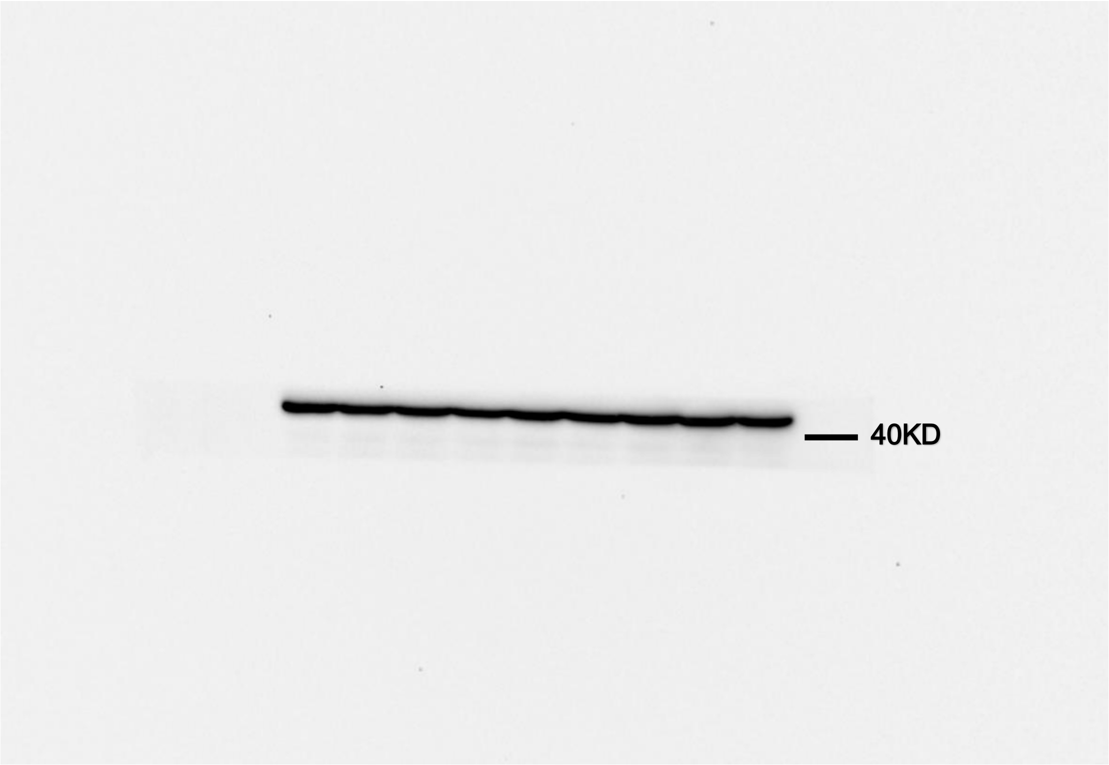

GPX4

From left to right: 6M *Cp*$^{fl/fl}$ 1, 6M *Cp*$^{fl/fl}$ 2, 6M *Cp*$^{fl/fl}$ 3, 18M *Cp*$^{fl/fl}$ 1, 18M *Cp*$^{fl/fl}$ 2, 18M *Cp*$^{fl/fl}$ 3, 18M *Cp*^{Gfap}*cKO* 1, 18M *Cp*^{Gfap}*cKO* 2, 18M *Cp*^{Gfap}*cKO* 3, 6M *Cp*$^{fl/fl}$ 4, 18M *Cp*$^{fl/fl}$ 4, 18M *Cp*^{Gfap}*cKO* 4

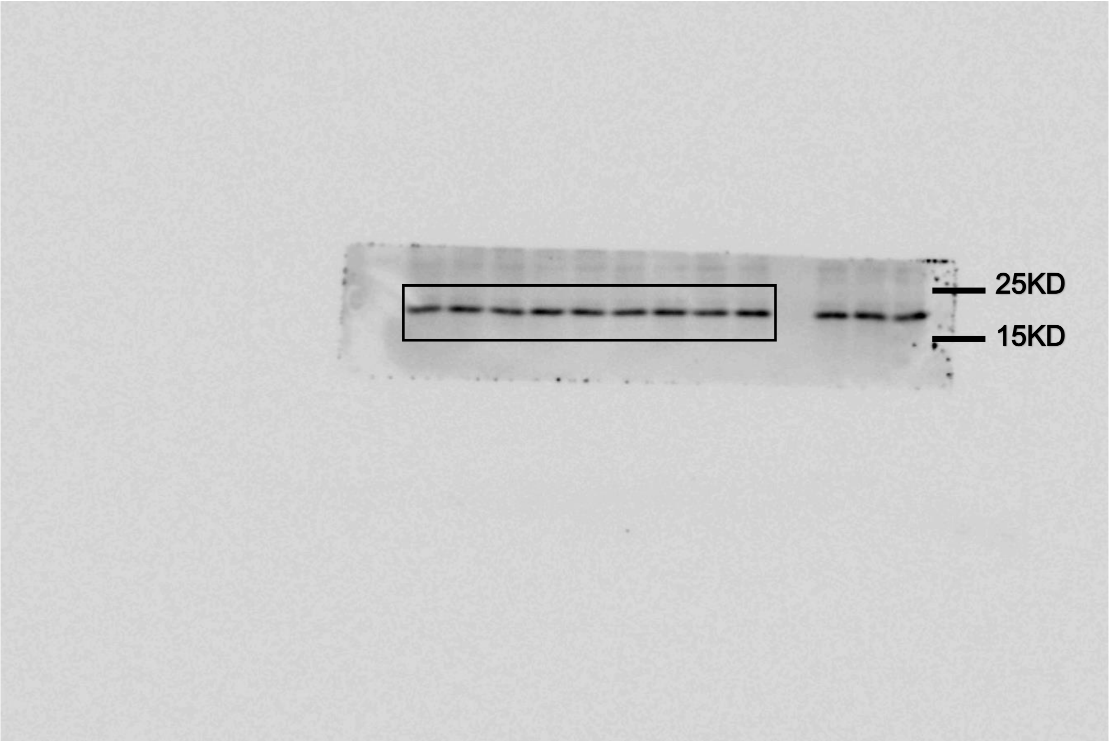

β-actin

From left to right: 6M *Cp*$^{fl/fl}$ 1, 6M *Cp*$^{fl/fl}$ 2, 6M *Cp*$^{fl/fl}$ 3, 18M *Cp*$^{fl/fl}$ 1, 18M *Cp*$^{fl/fl}$ 2, 18M *Cp*$^{fl/fl}$ 3, 18M *Cp*^{Gfap}*cKO* 1, 18M *Cp*^{Gfap}*cKO* 2, 18M *Cp*^{Gfap}*cKO* 3, 6M *Cp*$^{fl/fl}$ 4, 18M *Cp*$^{fl/fl}$ 4, 18M *Cp*^{Gfap}*cKO* 4

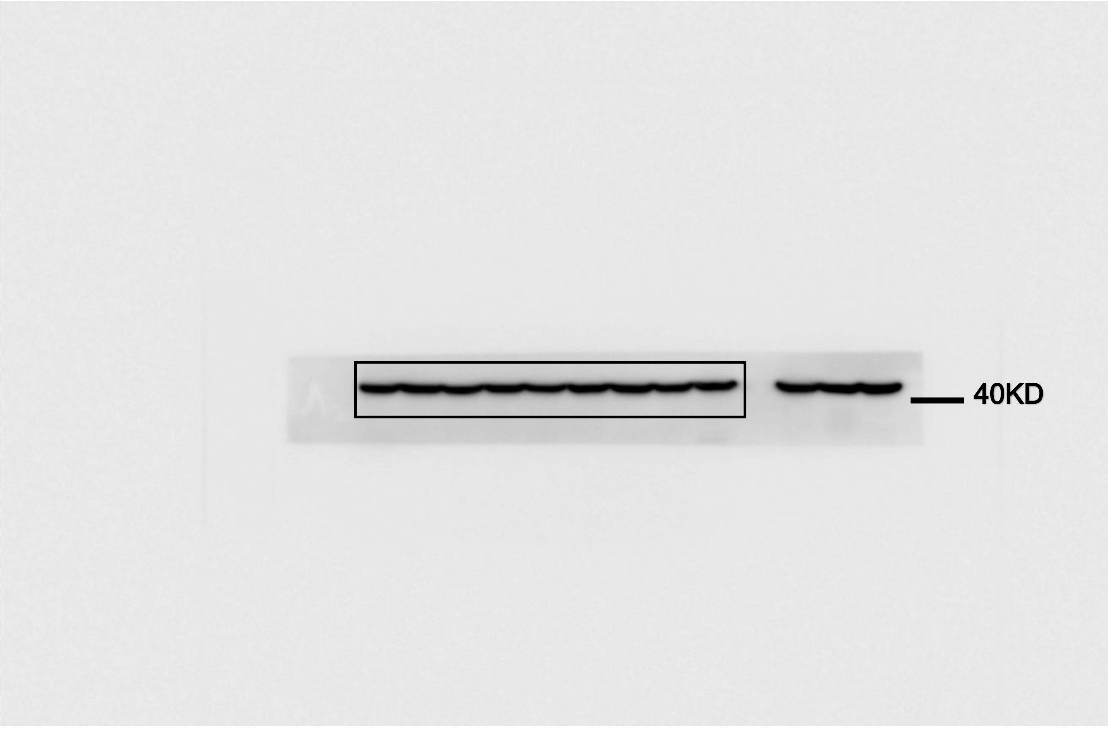

Figure 7T

Cerebral cortex

From left to right: 6M $Cp^{fl/fl}$ 1, 6M $Cp^{fl/fl}$ 2, 18M $Cp^{fl/fl}$ 1, 18M $Cp^{fl/fl}$ 2, 18M $Cp^{Gfap}cKO$ 1, 18M $Cp^{Gfap}cKO$ 2, 6M $Cp^{fl/fl}$ 3, 18M $Cp^{fl/fl}$ 3, 18M $Cp^{Gfap}cKO$ 3, 6M $Cp^{fl/fl}$ 4, 18M $Cp^{fl/fl}$ 4, 18M $Cp^{Gfap}cKO$ 4

p-Tau

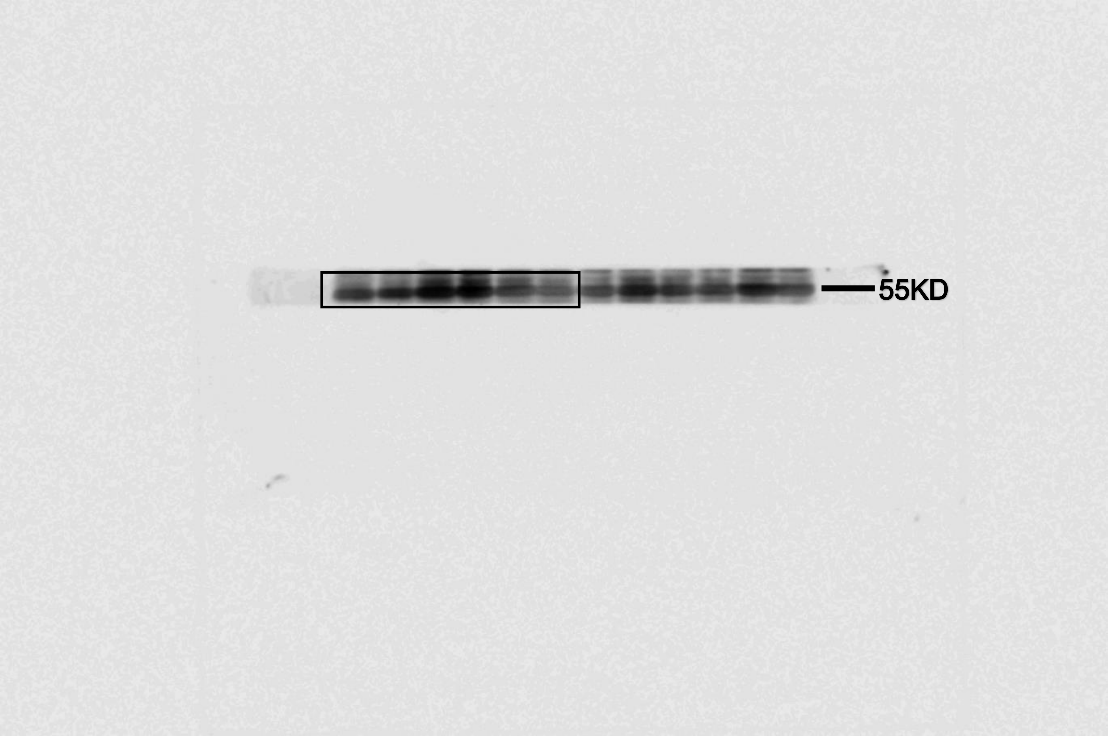

β-actin (The third time ECL from p-Erk/Erk Figure 8B)

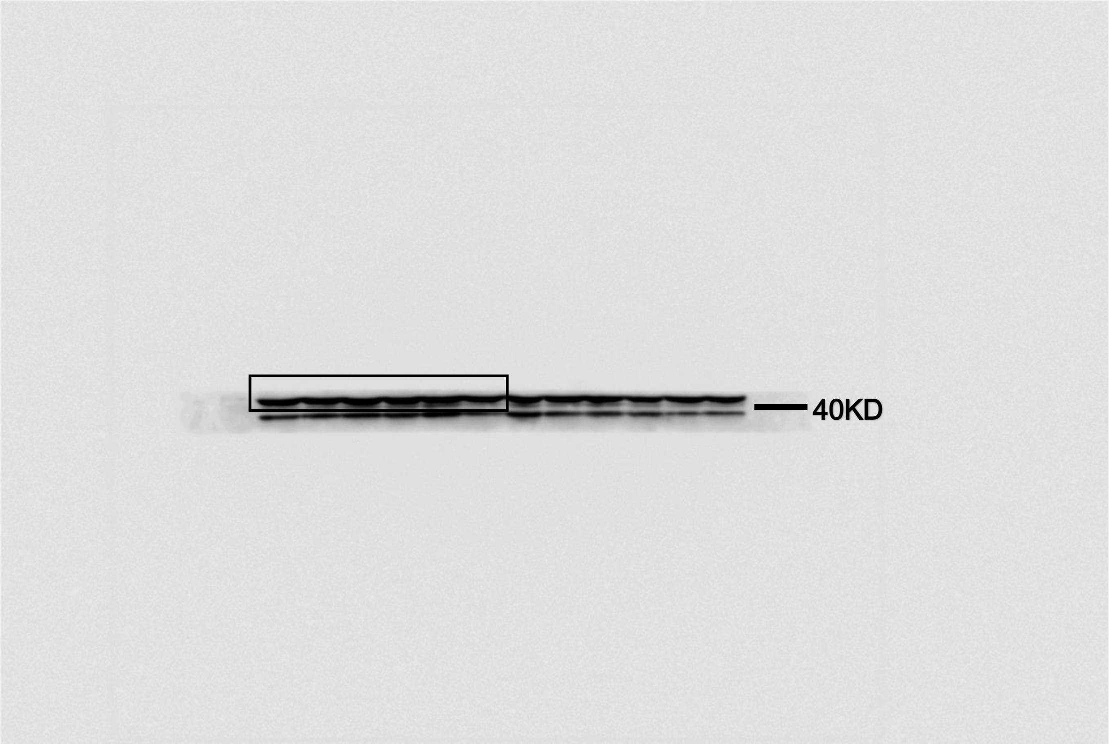

Hippocampus

From left to right: 6M *Cp*$^{fl/fl}$ 1, 6M *Cp*$^{fl/fl}$ 2, 18M *Cp*$^{fl/fl}$ 1, 18M *Cp*$^{fl/fl}$ 2, 18M *Cp*^{Gfap}*cKO* 1, 18M *Cp*^{Gfap}*cKO* 2, 6M *Cp*$^{fl/fl}$ 3, 18M *Cp*$^{fl/fl}$ 3, 18M *Cp*^{Gfap}*cKO* 3, 6M *Cp*$^{fl/fl}$ 4, 18M *Cp*$^{fl/fl}$ 4, 18M *Cp*^{Gfap}*cKO* 4

p-Tau

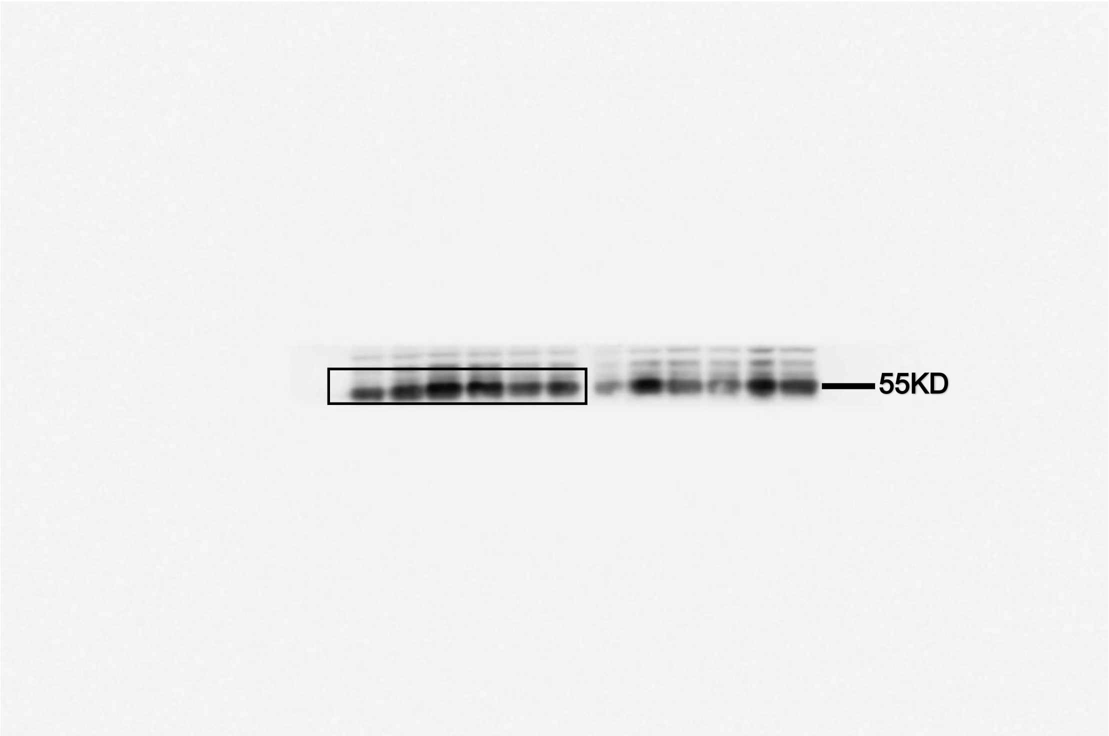

β-actin

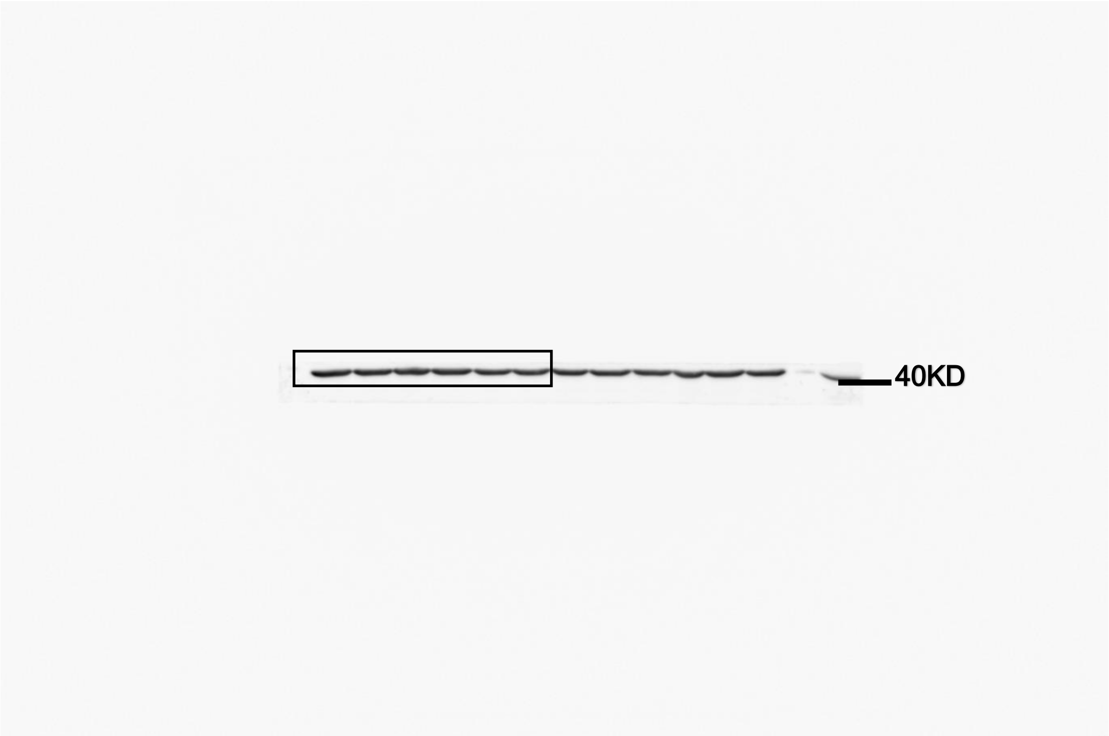

Figure 7W

From left to right: 6M $Cp^{fl/fl}$ 1, 6M $Cp^{fl/fl}$ 2, 6M $Cp^{fl/fl}$ 3, 18M $Cp^{fl/fl}$ 1, 18M $Cp^{fl/fl}$ 2, 18M $Cp^{fl/fl}$ 3, 18M $Cp^{Gfap}cKO$ 1, 18M $Cp^{Gfap}cKO$ 2, 18M $Cp^{Gfap}cKO$ 3

β-amyloid oligomers and sAPP

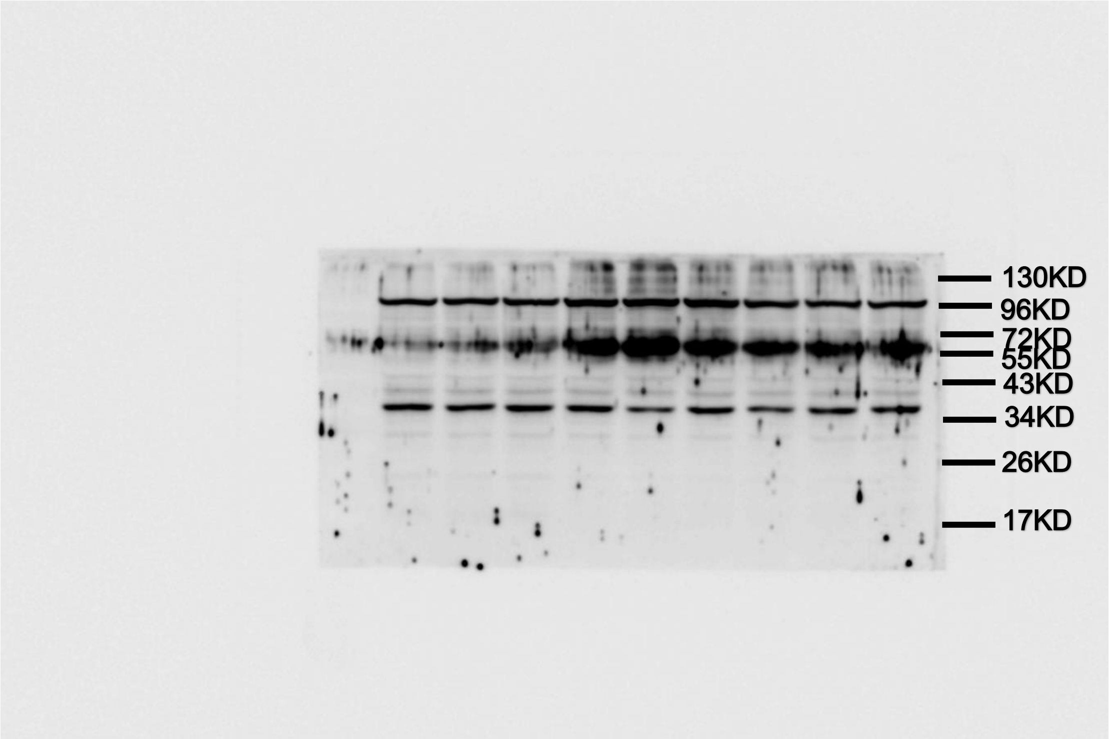

β-actin

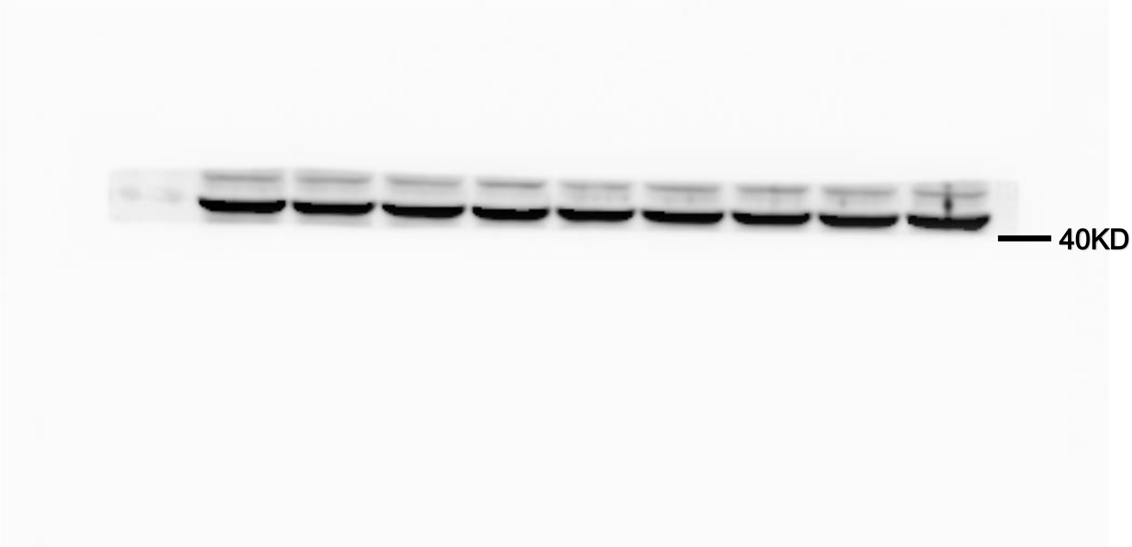

Figure S2A

From left to right: $Cp^{fl/fl}$ *1*, $Cp^{fl/fl}$ *2*, $Cp^{fl/fl}$ 3, $Cp^{Gfap}cKO$ 1, $Cp^{Gfap}cKO$ 2, $Cp^{Gfap}cKO$ 3

CP

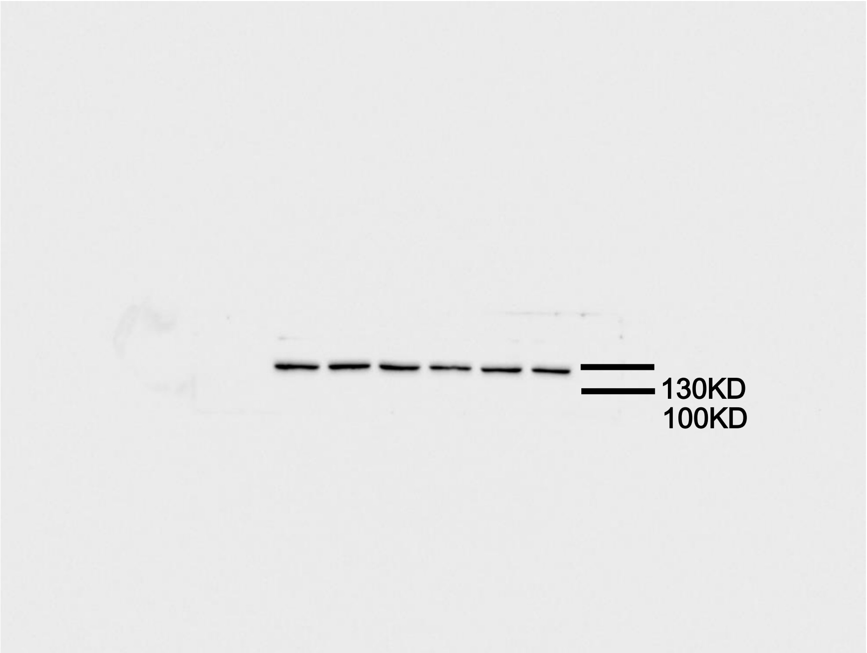

β-actin

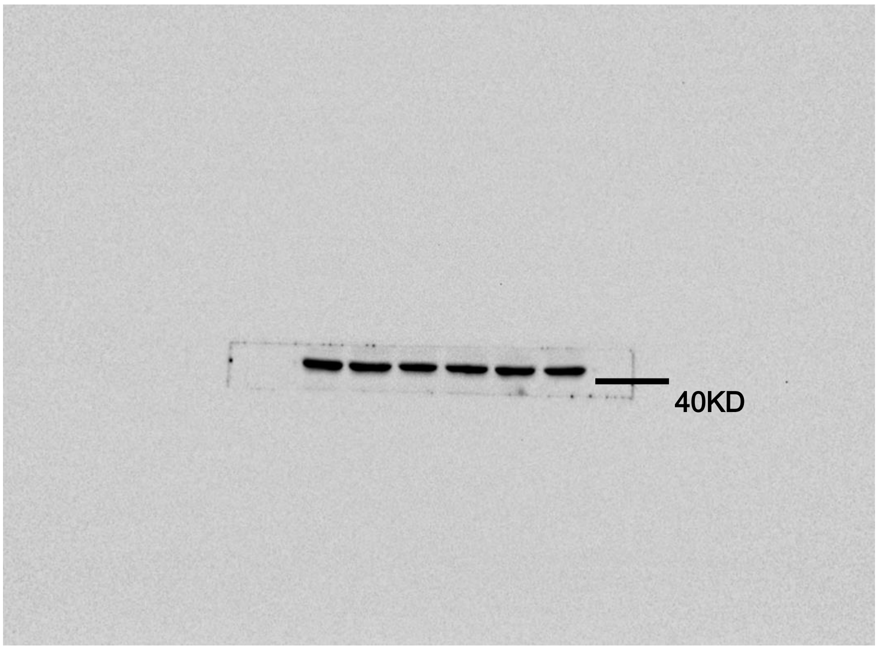

Figure S2B

From left to right: *Cp*$^{fl/fl}$ *1*, *Cp*$^{fl/fl}$ *2*, *Cp*$^{fl/fl}$ 3, *Cp*^{Gfap}*cKO* 1, *Cp*^{Gfap}*cKO* 2, *Cp*^{Gfap}*cKO* 3

CP

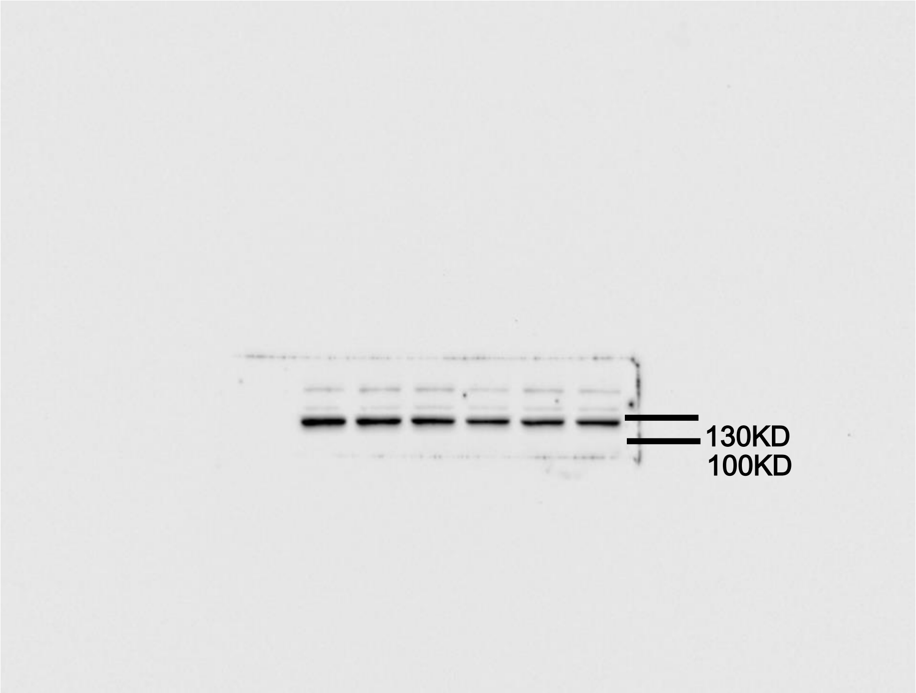

β-actin

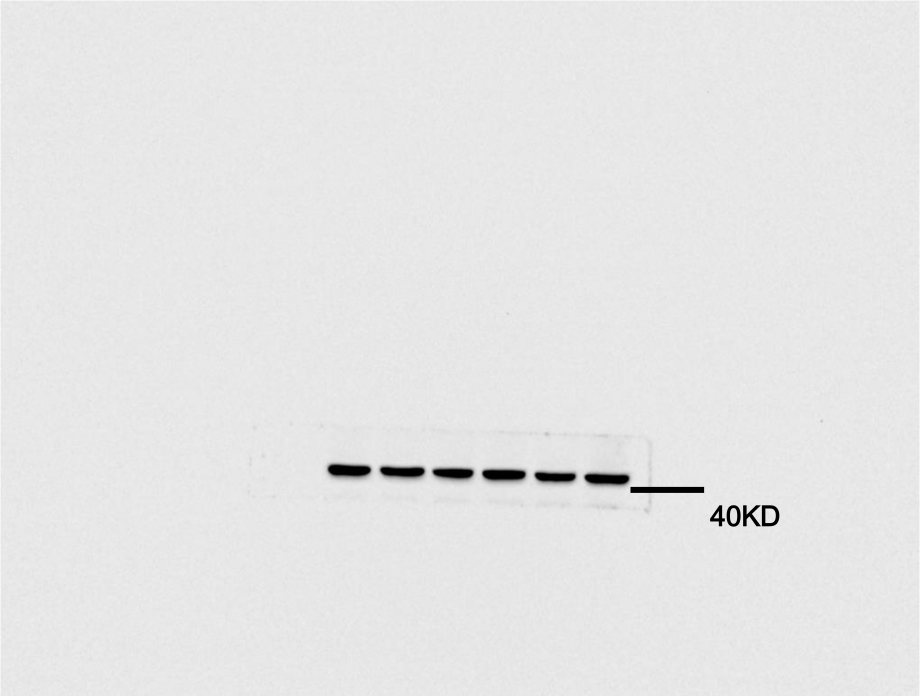

Figure S7A

From left to right: $Cp^{fl/fl}$ *1*, $Cp^{fl/fl}$ *2*, $Cp^{fl/fl}$ 3, Cp^{Gfap}*cKO* 1, Cp^{Gfap}*cKO* 2, Cp^{Gfap}*cKO* 3

CP

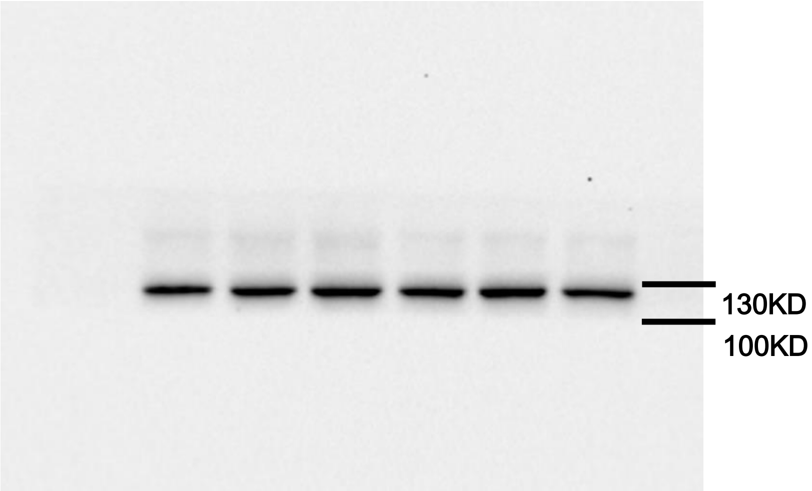

GAPDH

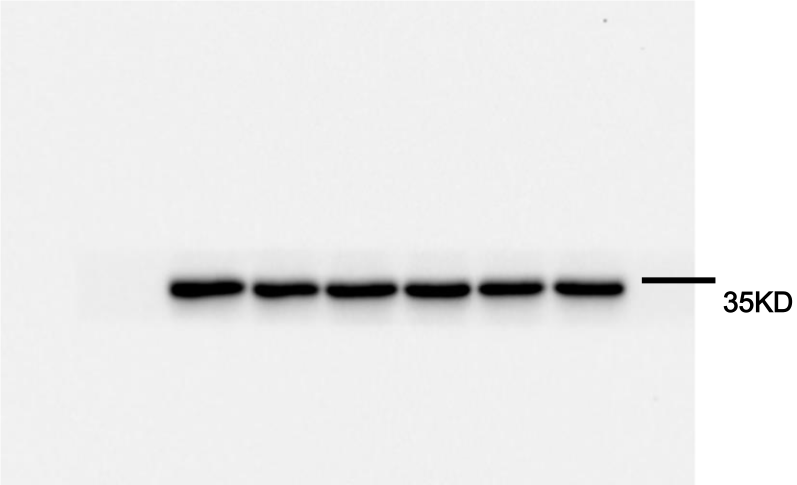

Figure S7B

CP

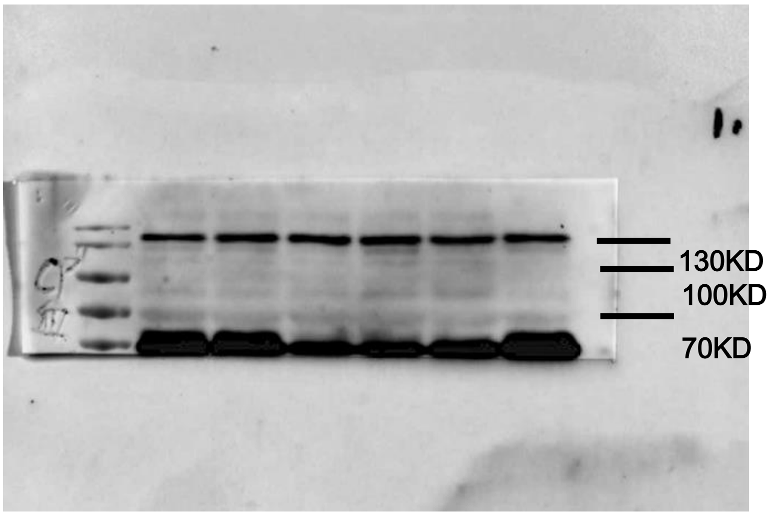

Supplement: Supplementary file 2 — Original Data File [file 41419_2022_5459_MOESM2_ESM.pdf]
